# Effects of electrical and magnetic stimulation on upper extremity function after stroke: A systematic review and network meta‐analysis

**DOI:** 10.1002/pmrj.13356

**Published:** 2025-05-21

**Authors:** Apisara Keesukphan, Monchai Suntipap, Kunlawat Thadanipon, Suparee Boonmanunt, Pawin Numthavaj, Gareth J. McKay, John Attia, Ammarin Thakkinstian

**Affiliations:** ^1^ Department of Clinical Epidemiology and Biostatistics, Faculty of Medicine Ramathibodi Hospital Mahidol University Bangkok Thailand; ^2^ Department of Rehabilitation Medicine Faculty of Medicine Ramathibodi Hospital, Mahidol University Bangkok Thailand; ^3^ Centre for Public Health, School of Medicine, Dentistry and Biomedical Sciences Queen's University Belfast Belfast UK; ^4^ Centre for Clinical Epidemiology and Biostatistics, School of Medicine and Public Health, Faculty of Health and Medicine Hunter Medical Research Institute, University of Newcastle New Lambton New South Wales Australia

## Abstract

**Objectives:**

To pool and rank the efficacy of various stimulation therapies, including repetitive peripheral magnetic stimulation (rPMS), neuromuscular electrical stimulation (NMES), functional electrical stimulation (FES), transcranial magnetic stimulation (TMS), and combinations of these interventions on upper extremity function, activities of daily living (ADL), and spasticity after stroke relative to sham/conventional rehabilitation.

**Literature Survey:**

MEDLINE, Scopus, Physiotherapy Evidence Database, Cochrane Central Register of Controlled Clinical Trials, and Google Scholar were searched from inception to July 2022.

**Methodology:**

Randomized controlled trials comparing any of the interventions mentioned above (rPMS, NMES, FES, TMS, NMES+rPMS, NMES+TMS, FES+TMS, and conventional rehabilitation) on upper extremity function, ADL, or spasticity from five databases were systematically reviewed and collected. Two‐stage network meta‐analysis was applied.

**Synthesis:**

Thirty‐four studies involving 1476 patients reporting upper extremity function with the Fugl‐Meyer Assessment were pooled. NMES combined with rPMS, NMES, NMES combined with TMS, TMS, and FES showed significantly higher improvement than conventional rehabilitation, with pooled mean differences (95% confidence intervals) of 14.69 (9.94–19.45), 9.09 (6.01–12.18), 6.10 (2.51–9.69), 4.07 (0.33–7.81), and 3.61 (0.14–7.07) respectively. NMES combined with rPMS had the highest probability for improving upper extremity function. NMES plus TMS had the highest probability for improving ADL, but none of the interventions showed significant differences in spasticity.

**Conclusions:**

NMES plus rPMS might be the best intervention to improve upper extremity functions, with NMES plus TMS most likely to lead to improved ADL but the quality of the evidence is low.

## INTRODUCTION

Rehabilitation is pivotal for the recuperation of patients after stroke,^1^ of whom approximately 25% to 50% are partially or totally dependent to undertake daily life activities,^2^ especially given deficits in upper extremity motor function.^3^ Currently, various neurorehabilitation interventions have been incorporated into standard rehabilitation practices to enhance neuroplasticity and aid in the recovery of upper extremity function in post‐stroke patients. These interventions include neuromuscular electrical stimulation (NMES), functional electrical stimulation (FES), transcranial magnetic stimulation (TMS), and repetitive peripheral magnetic stimulation (rPMS). TMS is subject to several side effects, such as headache, discomfort,^4^, ^5^ and unintentional seizures,^4^ and electrical stimulation (ES) may cause skin irritation and burns.^6^ Recently, rPMS has grown in popularity given the limitations and side effects associated with ES and TMS. rPMS is a noninvasive method of delivering a rapidly pulsed, high‐intensity magnetic field to the extremities. In poststroke rehabilitation, rPMS aims to improve motor function and neuromodulation in movement.

Several previous systematic reviews and pairwise meta‐analyses have been conducted,^7^, ^8^ but there was a dearth of evidence for the use of rPMS in patients after stroke. More recent studies^9^, ^10^, ^11^, ^12^ have suggested that rPMS may improve upper extremity function, leading to a better recovery than TMS in patients after stroke. To our knowledge, there has not been a previous network meta‐analysis comparing the efficacy of single or multiple interventions, including rPMS, TMS, or ES, relative to each other or sham/conventional rehabilitation for improving upper extremity function in poststroke patients. Therefore, this network meta‐analysis was conducted with the following objectives: first, to pool and rank the efficacy of various stimulation therapies on upper extremity function relative to conventional rehabilitation; and second, to pool and rank the efficacy of the aforementioned interventions on activities of daily living (ADL) and spasticity in poststroke patients.

## METHODS

This systematic review and network meta‐analysis was undertaken and reported according to the Preferred Reporting Items for Systematic Reviews and Meta‐analysis (PRISMA) extension for network meta‐analysis.^13^ Its protocol was prospectively registered in International Prospective Register of Systematic Reviews (CRD42022351051).

### Search strategy

We searched electronic databases, including MEDLINE via PubMed, Scopus, the Physiotherapy Evidence Database, and the Cochrane Central Register of Controlled Clinical Trials, from their inception to July 2022. The search terms were constructed based on relevant concepts as follows: (1) patients—stroke; (2) interventions/comparators—rPMS, TMS, NMES, FES; (3) outcomes— upper extremity function, ADL, spasticity; and (4) study type—randomized controlled trial (RCT), as shown in Appendix 1 in Data S1.

### Selection of studies

RCTs were considered eligible based on the following inclusion criteria: (1) conducted in adult patients aged 18 years or older with a poststroke condition or any patients with neurological disorder >50% attributable to stroke; (2) compared any regimen of interventions (ie, rPMS, TMS, ES, or their combinations) with any control intervention (ie, sham procedure, placebo, conventional rehabilitation, or a combination thereof); and (3) assessed at least one of the following outcomes: upper extremity functions, ADL, and spasticity.

Studies were excluded if they had insufficient data for pooling or were published in languages that reviewers could not translate.

### Interventions

The interventions included rPMS, TMS, and ES, or combinations thereof. rPMS was applied to the paretic upper limb using any regimen. The intensity was at least minimal muscle contraction with a frequency of 1–50 Hz and a duration of 1 minute. TMS was applied through the affected or unaffected cerebral hemisphere with an intensity of at least 50% of the motor threshold, focusing on upper extremity muscles and a frequency of 1–50 Hz. Two types of ES were considered: NMES used electrical current to produce minimal contraction of paretic muscles whereas FES used electrical stimulation during voluntary movement for functional purposes.

### Outcomes of interest

The primary outcome of interest was the upper extremity function after receiving the intervention. The measurement time was categorized as short term (at the end of the course of intervention) and long term (1–3 months after the course of intervention). Most studies used the Fugl‐Meyer Assessment^14^ (FMA), which measures the upper extremity's voluntary movement, reflex activity, grasp, and coordination. The total score ranges from 0 to 66 points; with higher score indicating better upper extremity performance.

The secondary outcomes of interest included ADL and spasticity. For ADL, most studies used the Barthel Index (BI)^15^ which ranges from 0 to 100 points, with a higher score indicative of more independence in ADL. The BI measures independence for 10 items of daily activities (feeding, moving from a wheelchair to bed and returning, using the toilet, getting on and off the toilet, bathing oneself, walking on a level surface, ascending and descending stairs, dressing, and controlling bowels and bladder). Each item is categorized to three options: unable to perform the task (score = 0), needing assistance (score = 0, 5, or 10 according to the item), and being fully independent (score = 5, 10, or 15 according to the item). For spasticity, the Modified Ashworth Scale (MAS)^16^ was used. MAS can range from 0 (no increase in muscle tone), 1 (slight increase in muscle tone, manifested by catch and release), 1+ (slight increase in muscle tone, manifested by catch, followed by minimal resistance), 2 (more marked increase in muscle tone through most of the range of motion), 3 (considerable increase in muscle tone, passive movement difficult), and 4 (affected part rigid in flexion or extension). A higher score reflects more spasticity.

### Data extraction and risk of bias assessment

Data extraction was performed by two reviewers (A.K., M.S.) and covered six domains: general information, study characteristics, participant characteristics, interventions, outcomes, and data for pooling, see details in Appendices 2 and 3 in Data S1. The quality of the studies was also independently assessed by the same reviewers using the Revised Cochrane Risk‐of‐Bias Tool for Randomized Trials (RoB 2.0).^17^ Five domains were assessed including randomization process, protocol deviations, missing outcome data, measurement of the outcome, and selection of the reported results. Each study was judged as low risk, high risk, or having some concerns. Any disagreements between reviewers were resolved by consensus (A.K., M.S., K.T., P.N., and A.T.).

### Statistical analysis

Pairwise meta‐analysis was performed on each intervention pair that was present in at least three studies. The unstandardized mean difference (USMD) from each study was estimated and pooled across the studies using a random‐effects model if heterogeneity was present, otherwise a fixed‐effect model was used. Cochrane's *Q* test and the *I* ^2^ statistic were used for assessing heterogeneity. Heterogeneity was considered present if the *p* value of Cochrane's *Q* test <.1 or *I* ^2^ ≥ 25%. Covariables were explored as a source of heterogeneity by fitting each covariable in a meta‐regression model; if a covariate reduced *τ* ^2^ (tau squared) by at least 50%, subgroup analysis on that covariable was performed.

Network meta‐analysis with the consistency model was performed using a two‐stage approach as follows. First, USMDs and their variance–covariance were estimated for each study using the sham procedure/conventional rehabilitation as the reference group. Second, multivariate random‐effects meta‐analysis was applied to pool USMDs across the studies. The transitivity assumption was checked by reviewing characteristics of the studies. The consistency assumption was checked using the design‐by‐treatment interaction inconsistency model.^18^, ^19^ Inconsistency was considered present when the *p* value of the global test was <.05.^20^ If this assumption was violated, a loop‐specific approach was applied to identify a specific loop of network meta‐analysis that caused inconsistency.^21^ Characteristics of the studies within the loop were explored and sensitivity analysis performed by excluding studies with different characteristics to achieve global consistency; if not achieved, the inconsistency model with design‐by‐treatment interaction, which adjusted the effects of the treatments from different study designs, was used to estimate the relative treatment effects. The surface under the cumulative ranking curves (SUCRA) was used to rank the best treatment with highest upper extremity functions or ADL score, and lowest spasticity score. Subgroup analysis was performed according to duration of stroke (acute‐subacute vs. chronic stroke) and stroke severity (low vs. high baseline functional FMA) in the outcomes with sufficient data. Publication bias was assessed using a comparison‐adjusted funnel plot and Egger's test. All analyses were conducted using Stata version 17.0 (StataCorp. 2022. Stata Statistical Software: Release 17. StataCorp LLC, College Station, TX).

The confidence in the network meta‐analysis findings was assessed using the web application Confidence in Network Meta‐Analysis (CINeMA). Six domains were considered based on the Grading of Recommendations Assessment, Development, and Evaluation (GRADE) approach, that is, within‐study bias, reporting bias, indirectness, imprecision, heterogeneity, and incoherence.^22^, ^23^

First, within‐study bias refers to bias in a study's design or conducting study that can systematically distort the estimated relative treatment effect, causing it to deviate from the true effect. This bias could be assessed using the RoB tool as mentioned. Second, reporting bias occurs when there is a systematic omission or distortion of study results, often due to publication bias (omitting non‐significant or “negative” studies), time‐lag bias (delaying publication of studies with unfavorable results), or outcome reporting bias (excluding unfavorable results from study reports). Third, the indirectness refers to the degree to which the included studies directly address the research question. This can arise when the study populations, interventions, outcomes, or study settings are not representative of the settings, populations, or outcomes for which inferences are being drawn. For example, we downgraded studies that included neurological patients rather than solely stroke patients. Fourth, imprecision of the effect size is assessed based on the equivalence zone. An estimate is considered imprecise if lower and upper limits of the 95% confidence interval (CI) fall outside the equivalence zone from no benefit to risk effect, and the other limit exceeds the equivalence zone; or both limits fall within the range of no benefit to risk. Fifth, heterogeneity, measured by tau,^2^ reflects the variation in effect sizes across included studies. This variation is accounted for when calculating the prediction interval. Heterogeneity is significant if the prediction interval includes values that lead to different conclusions compared to those drawn from the 95% CI alone. Sixth, incoherence is the disagreement of effect sizes estimated from direct and indirect comparisons. Each domain was graded as no concern, some concern, and major concern. Finally, each comparison was summarized across domains and graded level of confidence of the GRADE approach as very low, low, moderate, and high.^22^, ^23^

## RESULTS

### Characteristics of included studies

**FIGURE 1:**
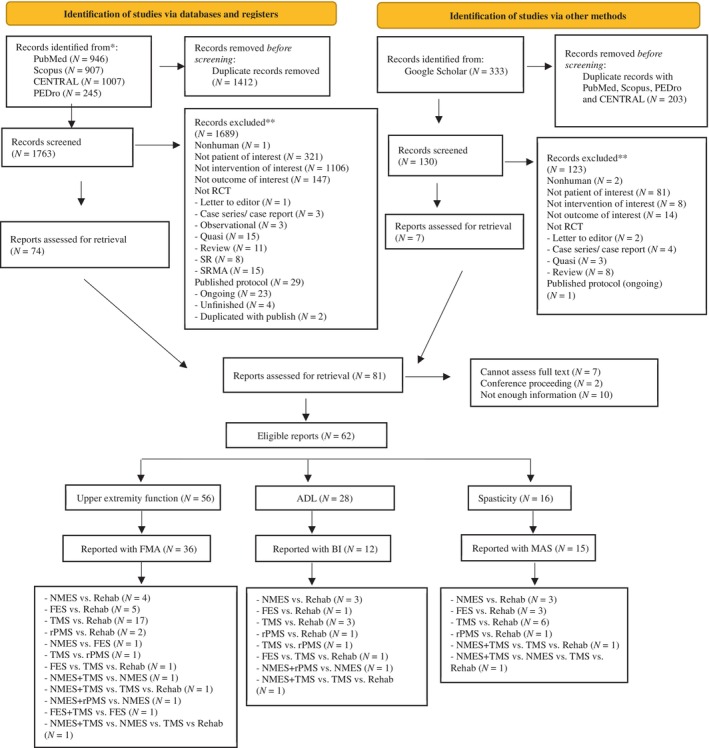
Preferred reporting items for systematic reviews and meta‐analysis (PRISMA) flow diagram of study selection. ADL, activities of daily living; BI, Barthel Index; CENTRAL, Cochrane Central Register of Controlled Clinical Trials; FES, functional electrical stimulation; FMA, Fugl‐Meyer Assessment; MAS, Modified Ashworth Scale; NMES, neuromuscular electrical stimulation; PEDro, Physiotherapy Evidence Database; RCT, randomized controlled trial; Rehab, conventional rehabilitation; rPMS, repetitive electrical magnetic stimulation; SR, systematic review; SRMA, systematic review and meta‐analysis; TMS, transcranial magnetic stimulation.

Of 2096 identified studies, 81 full papers were screened, leaving 62 eligible and included in this review. Reasons for exclusions are shown in the PRISMA flow chart (Figure 1). Characteristics of the eligible studies are summarized in Table 1. There were five single interventions (ie, NMES, FES, TMS, rPMS, and conventional rehabilitation) and three combination interventions (ie, NMES+TMS, NMES+rPMS, and FES + TMS); see more details in Table 1 and Appendix 4 in Data S1. Percentage of males ranged from 3.6 to 93.8, and mean age from 45.5 to 74.5 years. Most studies (98%) included patients with ischemic stroke as the majority of their participants. The percentage of right hemiparesis with left dominant hemisphere lesion ranged from 26.7 to 77.8.

**TABLE 1:** Characteristics of included studies.

References	Year	n	Mean age (year)	Male (%)	Mean duration of disease (months)	Ischemic stroke (%)	Left hemiparesis (%)	Intervention	Duration of intervention (days)	Upper extremity function‐measurement	ADL‐measurement	Spasticity‐measurement
Ke et al.^11^	2022	26	57	53.8	0.54	0	46.2	rPMS vs. Rehab	10	FMA	‐	‐
Jiang et al.^10^	2022	44	55.3	61.6	0.46	72.7	38.6	NMES + rPMS vs. NMES	14	FMA	BI	‐
El Nahas. et al.^62^	2022	64	46.0	75	46.35	NA	NA	rPMS vs. Rehab	8	‐	‐	MAS
Krewer et al.^41^	2014	40	54.5	60.3	7.15	NA	52.4	rPMS vs. Rehab	10	FMA	BI	mTS
Luk et al.^44^	2022	37	66.2	58.3	3.30	87.5	58.3	TMS vs. Rehab	10	FMA, ARAT, BBT	‐	‐
Haghighi et al.^33^	2021	24	52.2	50	3.10	45	55	TMS vs. Rehab	10	FMA, BBT	‐	‐
Gottlieb et al.^31^	2021	20	63.1	42.9	1.41	89.3	25	FES + TMS vs. FES	10	FMA	‐	MAS
Sharma et al.^56^	2020	96	53.8	72.9	0.16	100	NA	TMS vs. Rehab	14	FMA	mBI	MAS
Kim et al.^40^	2020	77	62.1	61.65	0.49	100	50.7	TMS vs. Rehab	14	FMA, BBT	mBI	MAS
Chen et al.^27^	2019	22	52.8		6.00	22.5	68.2	TMS vs. Rehab	10	FMA, ARAT	‐	‐
Tretriluxana et al.^87^	2018	16	52.2	3.6	3.57	NA	NA	TMS vs. Rehab	1	WMFT‐time	‐	‐
Harvey et al.^34^	2018	199	58.7	65.3	7.71	78.9	53	TMS vs. Rehab	18	FMA, ARAT, WMFT‐time	‐	‐
Wang et al.^54^	2017	42	60.8	69.0	2.30	69.0	40.5	TMS vs. Rehab	24	FMA, WMFT	‐	‐
Ozjeskin et al.^63^	2017	21	60.3	61.9	17.81	NA	52.4	TMS vs. Rehab	10	‐	‐	MAS
Meng et al.^58^	2017	20	65	85	NA	100	NA	TMS vs. Rehab	14	‐	BI	‐
Guan et al.^32^	2017	42	58.5	71.43	0.23	100	54.8	TMS vs. Rehab	10	FMA	BI	‐
Askin et al.^24^	2017	40	57.8	72.5	26.4	100	57.5	TMS vs. Rehab	10	FMA, BBT	FIM	MAS
Hosomi et al.^35^	2016	39	62.8	59.2	1.84	61.62	61.5	TMS vs. Rehab	10	FMA	FIM	‐
Ackerley et al.^88^	2016	18	66	66.7	19.00	100	33.3	TMS vs. Rehab	10	ARAT	‐	‐
Matsuura et al.^45^	2015	20	73.4	55	0.32	100	50	TMS vs. Rehab	5	FMA	‐	‐
Vaziri et al.^53^	2014	12	56.7	NA	23.50	NA	NA	TMS vs. Rehab	10	FMA	BI	‐
Rose et al.^47^	2014	21	64.6	68.4	61.66	NA	47.4	TMS vs. Rehab	16	FMA, WMFT, WMFT‐time	‐	MAS
Galvao et al.^30^	2014	20	61	65	53.35	85	50	TMS vs. Rehab	10	FMA	FIM	MAS
Hsu et al.^36^	2013	12	59.6	66.7	0.70	100	NA	TMS vs. Rehab	10	FMA, ARAT	‐	‐
Higgins et al.^89^	2013	11	66.2	66.7	108.33	NA	22.2	TMS vs. Rehab	8	BBT, WMFT, WMFT‐time	‐	‐
DiLazzaro et al.^90^	2013	12	58.5	58.3	32.40	NA	NA	TMS vs. Rehab	10	ARAT, JTT	‐	‐
Seniow et al.^49^	2012	40	63.4	65	1.31	87.5	42.5	TMS vs. Rehab	15	FMA, WMFT, WMFT‐time	‐	‐
Conforto et al.^64^	2012	30	55.8	61.7	0.91	100	50	TMS vs. Rehab	10	‐	‐	MAS
Sohn et al.^65^	2010	13	54.2	61.5	53.88	NA	46.2	TMS vs. Rehab	10	Motor function test	‐	MAS
Malcolm et al.^91^	2007	19	67	42.1	45.60	NA	47.4	TMS vs. Rehab	10	BBT, WMFT‐time	‐	‐
Kim et al.^92^	2021	33	NA	NA	6.00	NA	NA	TMS vs. Rehab	20	Motor function test	mBI	‐
Fletcher‐Smith et al.^57^	2019	40	71	50	0.06	NA	55	NMES vs. Rehab	60	ARAT	BI	‐
Dorsch et al.^93^	2014	33	67.6	54.6	0.52	NA	42.6	NMES vs. Rehab	20	Motor assessment scale	‐	‐
Sahin et al.^94^	2012	42	59.8	57.1	30.05	NA	NA	NMES vs. Rehab	20	‐	FIM	‐
Rosewillium et al.^61^	2012	90	72.6	50.2	1.38	NA	49	NMES vs. Rehab	30	ARAT	BI	‐
Lin et al.^42^	2011	37	64.5	59.5	1.39	67.6	59.5	NMES vs. Rehab	15	FMA	mBI	MAS
Church et al.^95^	2006	176	74.5	50.6	0.33	93.6	63.6	NMES vs. Rehab	12	ARAT	‐	‐
Mann et al.^96^	2005	22	69.4	42.8	7.03	95.2	54.6	NMES vs. Rehab	84	ARAT	‐	‐
Kimberley et al.^97^	2004	16	60.1	68.8	33.44	NA	50	NMES vs. Rehab	10	BBT, JTT	‐	‐
Powell et al.^60^	1999	60	67.7	46.7	0.77	NA	63.3	NMES vs. Rehab	56	ARAT	BI	MAS
Francisco et al.^29^	1998	16	65.5	44.4	0.59	100	62.5	NMES vs. Rehab	29.4	FMA	FIM	‐
Chae et al.^25^	1998	28	59.7	46.4	0.52	89.3	53.6	NMES vs. Rehab	15	FMA	FIM	‐
Niu et al.^46^	2022	20	60.1	93.8	4.07	100	NA	NMES vs. Rehab	5	FMA	‐	‐
Kirac‐Unal et al.^98^	2018	27	65.6	51.9	0.46	100	52.6	FES vs. Rehab	20	ARAT	FIM	‐
Karaahmet et al.^38^	2019	21	56.9	62	1.37	85.6	52.1	FES vs. Rehab	20	FMA	FIM	‐
Nakipoglu‐Yuzer et al.^59^	2017	30	58.9	56.7	0.16	80	60	FES vs. Rehab	20	UEFT	BI	‐
Jonsdottir et al.^37^	2017	68	68.0	42.6	3.71	80.9	NA	FES vs. Rehab	25	FMA, ARAT	‐	‐
Thorsen et al.^99^	2013	11	48.8		8.55			FES vs. Rehab	25	ARAT	‐	‐
Karakus et al.^100^	2013	28	59.0	53.6	3.29	85.7	53.6	FES vs. Rehab	10	Total motricity index	‐	‐
Mohamed‐Faisal et al.^101^	2012	30	NA	NA	0.92	NA	NA	FES vs. Rehab	24	ARAT, BBT	‐	‐
Mangold et al.^66^	2009	23	59.6	73.9	1.61	82.5	69.6	FES vs. Rehab	12	‐	eBI	MAS
Chan et al.^26^	2009	20	45.5	55	15.10	NA	NA	FES vs. Rehab	15	FMA	FIM	MAS
Shin et al.^102^	2008	14	57.6	85.7	19.15	NA	50	FES vs. Rehab	50	BBT	‐	‐
Jian et al.^43^	2005	48	59.4	70.8	NA	68.8	NA	NMES vs. Rehab	36	FMA	‐	‐
Schick et al.^48^	2022	12	65	66.7	3.45	100	41.7	FES vs. Rehab	15	FMA, BBT	‐	‐
Chen et al.^9^	2020	40	50.8	77.1	1.50	NA	51.4	FES vs. NMES	10	FMA	BI	‐
Khan et al.^39^	2019	60	63.5	65	0.55	100	30	rPMS vs. TMS	12	FMA	BI	
Tosun et al.^52^	2022	25	58.5	56	1.69	100	48	TMS vs. FES vs. Rehab	10	Total motricity index	BI	MAS
Du et al.^28^	2022	240	58.3	51.7	47.73	74.6	56.7	NMES + TMS vs. TMS vs. Rehab	20	FMA	mBI	MAS
Tarri et al.^50^	2022	24	50.1	66.7	2.32	NA	NA	NMES + TMS vs. NMES vs. TMS vs. Rehab	5	FMA	‐	‐
Tilkici et al.^51^	2017	40	62.9	45	10.50	77.5	50	NMES + TMS vs. NMES	15	FMA	FIM	MAS
Blesneag et al.^55^	2015	16	69	62.5	0.33	NA	NA	NMES vs. Rehab	10	FMA	‐	‐

Abbreviations: ARAT, Action Research Arm Test; BBT, Box and Block Test of manual dexterity; BI, Barthel Index; eBI, Extended Barthel Index; FES, functional electrical stimulation; FIM, Functional Independence Measure; FMA, Fugl‐Meyer Assessment; JTT, Jebsen Taylor Hand Function Test; MAS, Modified Ashworth Scale; mBI, Modified Barthel index, mTS, Modified Tardieu scale; NA, not applicable; NMES, neuromuscular electrical stimulation; Rehab, conventional rehabilitation; rPMS, repetitive electrical magnetic stimulation; TMS, transcranial magnetic stimulation; UEFT, upper extremity function test; WMFT, Wolf Motor Function Test.

### Risk of bias

For the overall risk of bias, 41 studies (66.1%) were judged to have some concerns, primarily related to bias in the randomization process, whereas 10 studies (16.1%) were judged to have a low risk of bias and 11 (17.7%) studies were judged to have high risk of bias in the randomization process; see Appendix 5 in Data S1.

### Upper extremity function

Thirty‐four studies^9^, ^10^, ^11^, ^24^, ^25^, ^26^, ^27^, ^28^, ^29^, ^30^, ^31^, ^32^, ^33^, ^34^, ^35^, ^36^, ^37^, ^38^, ^39^, ^40^, ^41^, ^42^, ^43^, ^44^, ^45^, ^46^, ^47^, ^48^, ^49^, ^50^, ^51^, ^52^, ^53^, ^54^ reported FMA at the end of the intervention course, and another 10 studies^25^, ^30^, ^35^, ^36^, ^40^, ^44^, ^49^, ^54^, ^55^, ^56^ reported FMA at 1–3 months post intervention. Pairwise meta‐analysis was performed on three comparisons with FMA measured at the end of intervention course from at least three studies, that is, NMES (*N* = 5), FES (*N* = 6), and TMS (*N* = 19) versus conventional rehabilitation. NMES, FES, and TMS significantly improved FMA compared with conventional rehabilitation with USMDs of 7.28 (95% CI, 2.68–11.88), 5.37 (95% CI, 1.25–9.49), and 2.97 (95% CI, 0.30–5.64), respectively, with moderate to high heterogeneity detected (Appendix 6 in Data S1). The source of heterogeneity was explored identifying baseline FMA, duration of intervention, TMS type, and side of lesion as potential causes; see details in Appendix 6 in Data S1. TMS significantly improved FMA during the 1–3‐month follow‐up period when compared to conventional rehabilitation, with an USMD of 3.55 (95% CI, 1.04–6.95; *I* ^2^ = 16.23%).

**FIGURE 2:**
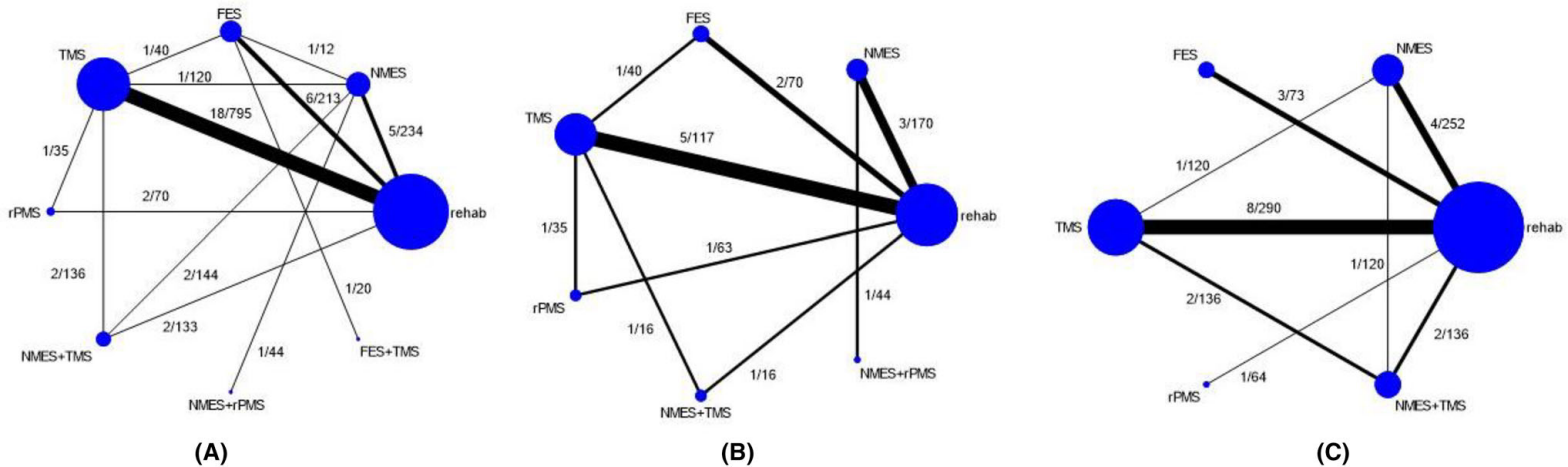
Network maps of the (A) Fugl‐Meyer Assessment, (B) Barthel Index, and (C) Modified Ashworth Scale outcomes assessed at the end of intervention course, with nodes and edges weighted by the number of studies and included patients, respectively. FES, functional electrical stimulation; NMES, neuromuscular electrical stimulation; rPMS, repetitive electrical magnetic stimulation; TMS, transcranial magnetic stimulation.

**FIGURE 3:**
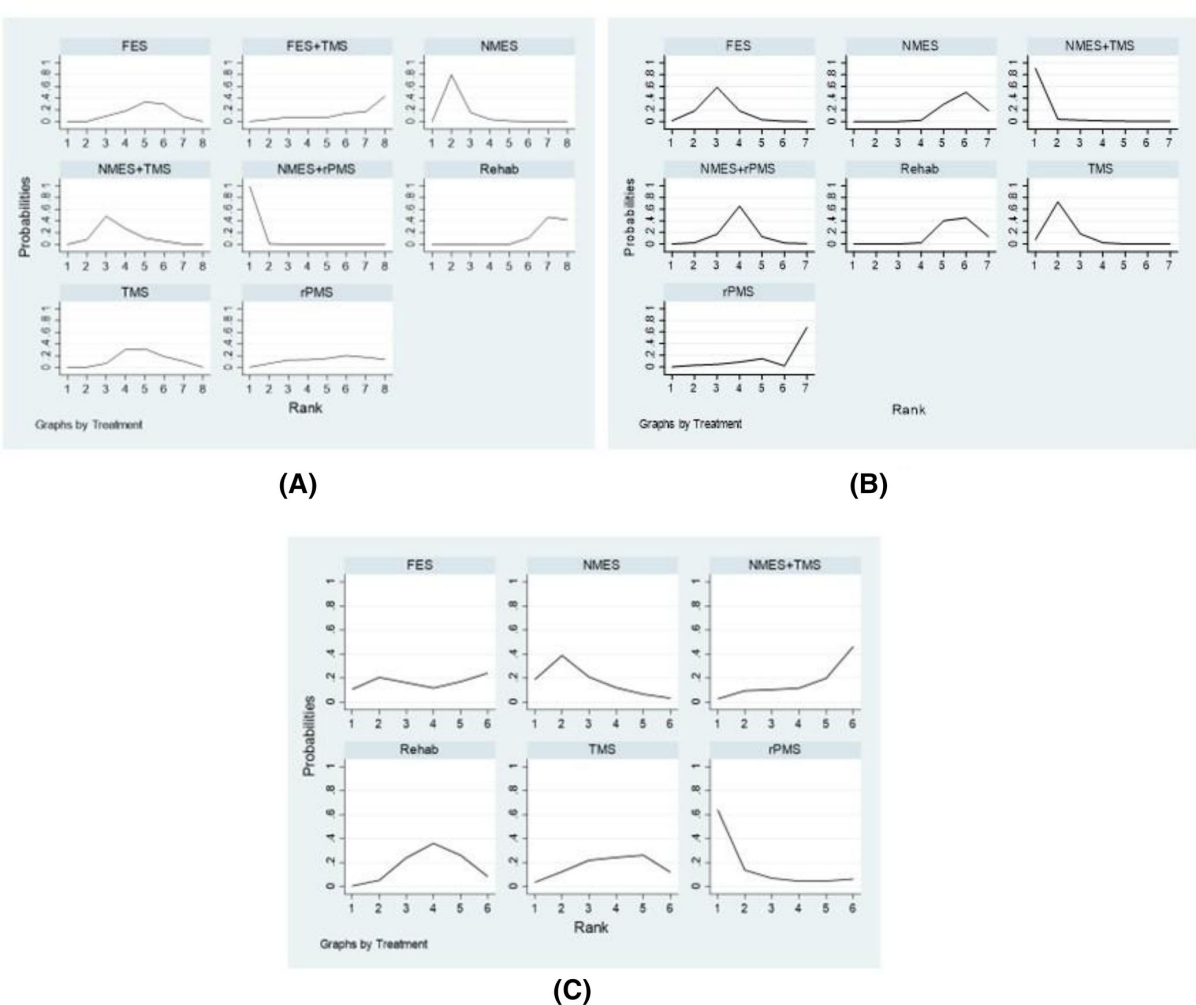
Ranking curves showing the probability of being the best intervention in terms of the (A) Fugl‐Mayer Assessment, (B) Barthel index, and (C) Modified Ashworth Scale outcomes at the end of intervention. FES, functional electrical stimulation; NMES, neuromuscular electrical stimulation; rPMS, repetitive electrical magnetic stimulation; TMS, transcranial magnetic stimulation.

Data from 34 studies involving 1476 patients were included in a network meta‐analysis using the inconsistency model. The consistency assumption checking is detailed in Appendix 8 in Data S1. For the FMA outcome measured at the end of intervention course (*N* = 34), the network meta‐analysis map consisted of eight interventions (ie, NMES, FES, TMS, rPMS, NMES+TMS, NMES+rPMS, FES+TMS, and conventional rehabilitation) with 13 pairwise comparisons, see Figure 2A. Five interventions, that is, NMES+rPMS, NMES, NMES+TMS, TMS, and FES, showed significantly improved FMA when compared to conventional rehabilitation, with USMDs of 14.69 (95% CI, 9.94–19.45), 9.09 (95% CI, 6.01–12.18), 6.10 (95% CI, 2.51–9.69), 4.07 (95% CI, 0.33–7.81), and 3.61 (95% CI, 0.14–7.07), respectively; see Table 2. The interventions with the top four ranked by SUCRA for increasing FMA were NMES+rPMS (SUCRA = 99.8), NMES (81.9), NMES+TMS (63.1), and TMS (43.5), respectively; see Table 2 and Figure 3A.

**TABLE 2:** Multiple treatment comparisons of interventions on upper extremity function measured by FMA at the end of intervention courses.

Reference treatment	Unstandardized mean difference							
	Rehab	FES+TMS	NMES+rPMS	NMES+TMS	rPMS	TMS	FES	NMES
Rehab	10, 0.0	0.41 (−9.48 to 10.29)	14.69 (9.94 to 19.45)	6.10 (2.51 to 9.69)	3.14 (−4.54 to 10.81)	4.07 (0.33 to 7.81)	3.61 (0.14 to 7.07)	9.09 (6.01 to 12.18)
FES+TMS	−0.41 (−10.29 to 9.48)	22.4, 0.3	14.29 (3.32 to 25.25)	5.69 (−4.82 to 16.21)	2.73 (−9.78 to 15.24)	3.66 (−6.91 to 14.23)	3.20 (−6.06 to 12.46)	8.69 (−1.66 to 19.04)
NMES+rPMS	−14.69 (−19.45 to −9.94)	−14.29 (−25.25 to −3.32)	99.8, 98.7	−8.59 (−14.55 to −2.63)	−11.56 (−20.58 to −2.53)	−10.62 (−16.67 to −4.58)	−11.09 (−16.96 to −5.22)	−5.60 (−9.22 to −1.98)
NMES+TMS	−6.10 (−9.69 to −2.51)	−5.69 (−16.21 to 4.82)	8.59 (2.63 to 14.55)	63.1, 0.1	−2.96 (−11.44 to 5.51)	−2.03 (−5.75 to 1.69)	−2.49 (−7.49 to 2.50)	2.99 (−1.74 to 7.73)
rPMS	−3.14 (−10.81 to 4.54)	−2.73 (−15.24 to 9.78)	11.56 (2.53 to 20.58)	2.96 (−5.51 to 11.44)	37.9, 0.7	0.93 (−7.06 to 9.47)	0.47 (−7.95 to 8.89)	5.96 (−2.31 to 14.22)
TMS	−4.07 (−7.81 to −0.33)	−3.66 (−14.23 to 6.91)	10.62 (4.58 to 16.67)	2.03 (−1.69 to 5.75)	−0.93 (−9.47 to 7.06)	43.5, 0.0	−0.46 (−5.56 to 4.63)	5.02 (0.18 to 9.87)
FES	−3.61 (−7.07 to −0.14)	−3.20 (−12.46 to 6.06)	11.09 (5.22 to 16.96)	2.49 (−2.50 to 7.49)	−0.47 (−8.89 to 7.95)	0.46 (−4.63 to 5.56)	41.5 to 0.0	5.49 (0.87 to 10.11)
NMES	−9.09 (−12.18 to −6.01)	−8.69 (−19.04 to 1.66)	5.60 (1.98 to 9.22)	−2.99 (−7.73 to 1.74)	−5.96 (−14.22 to 2.31)	−5.02 (−9.87 to −0.18)	−5.49 (−10.11 to −0.87)	81.9, 0.2

*Note*: Results in the off‐diagonal cells are the mean difference and 95% confidence intervals of the FMA from the network meta‐analysis. Each diagonal cell contains the surface under the cumulative ranking and probability of being the best treatment of each intervention.

Abbreviations: FES, functional electrical stimulation; FMA, Fugl‐Meyer Assessment; NMES, neuromuscular electrical stimulation; Rehab, conventional rehabilitation; rPMS, repetitive electrical magnetic stimulation; TMS, transcranial magnetic stimulation.

Subgroup analyses were performed by stroke subtypes (see Appendix 7A–E in Data S1) and TMS frequency low (≤1 Hz) and high (≥5 Hz) (see Appendix 7A,F–G in Data S1). For stroke subtypes, NMES+rPMS was top rank in improving FMA scores in acute/subacute and severe stroke (baseline FMA < 25) with USMDs of 11.93 (95% CI, 8.27–15.59) and 14.63 (95% CI, 8.87–20.40), respectively (Appendix 7B,D in Data S1). In chronic stroke, NMES was ranked first with a significant USMD of 12.00 (95% CI, 8.22–15.77) relative to conventional rehabilitation, whereas none of the interventions significantly improved FMA in the subgroup with less severe stroke (baseline FMA ≥25); see Appendix 7C,E in Data S1. High‐frequency TMS (≥5 Hz) showed a trend toward greater FMA improvement than low‐frequency TMS (≤1 Hz), with USMDs of 10.6 (95% CI, 4.7–16.5) and 4.07 (95% CI, −1.12 to 9.26), respectively (Appendix 7F,G in Data S1).

The CINeMA framework assigned very low to low confidence ratings to all pairwise comparisons. This was primarily due to incoherence major concerns and some additional concerns regarding within‐study bias, imprecision, and heterogeneity (Appendix 9 in Data S1).

### Activities of daily living

Twelve studies^9^, ^10^, ^32^, ^39^, ^41^, ^52^, ^53^, ^57^, ^58^, ^59^, ^60^, ^61^ reported BI at the end of intervention course. Two comparisons were available for pairwise meta‐analysis: NMES and TMS versus conventional rehabilitation. Only TMS showed significantly improved BI compared to conventional rehabilitation, with an USMD of 7.94 (95% CI, 0.44–15.44; *I* ^2^ = 79.11%), see Appendix 6 in Data S1.

Data from 12 RCTs involving 503 patients were pooled applying network meta‐analysis with an inconsistency model, which included nine pairwise comparisons among seven interventions, as shown in Figure 2B. The consistency assumption checking is detailed in Appendix 8 in Data S1. BI showed significant improvement in three interventions relative to conventional rehabilitation, that is, NMES+TMS, TMS, and FES, with USMDs of 30.89 (95% CI, 5.18–56.62), 12.25 (95% CI, 6.01–18.49), and 8.34 (95% CI, 2.37–14.31), respectively, see Table 3. In the ranking by SUCRA, the top four interventions were NMES+TMS (SUCRA = 96.2), TMS (80.8), FES (66.0), and NMES+rPMS (50.6), respectively, as shown in Table 3 and Figure 3B.

**TABLE 3:** Multiple treatment comparisons of interventions on ADL measured by BI at the end of intervention courses.

Reference treatment	Unstandardized mean difference						
	Rehab	NMES+rPMS	NMES+TMS	rPMS	TMS	FES	NMES
Rehab	22.0, 0.0	4.79 (−0.30 to 9.87)	30.89 (5.18 to 56.62)	−4.67 (−22.01 to 12.67)	12.25 (6.01 to 18.49)	8.34 (2.37 to 14.31)	−0.21 (−1.85 to 14.42)
NMES+rPMS	−4.76 (−9.87 to 0.30)	50.6, 0.1	26.11 (−0.10 to 52.33)	−9.46 (−27.52 to 8.61)	7.46 (−0.58 to 15.51)	3.55 (−4.29 to 11.40)	−5.00 (−9.82 to −0.18)
NMES+TMS	−30.89 (−56.62 to −5.18)	−26.11 (−52.33 to 0.10)	96.2, 90.5	−35.57 (−66.59 to −4.55)	−18.65 (−45.12 to 7.81)	−22.56 (−48.96 to 3.84)	−31.11 (−56.89 to −5.34)
rPMS	4.67 (−12.67 to 22.01)	9.46 (−8.61 to 27.52)	35.57 (4.55 to 66.59)	15.0, 0.2	16.92 (−1.51 to 35.34)	13.01 (−5.33 to 31.35)	4.46 (−12.96 to 21.87)
TMS	−12.25 (−18.49 to −6.01)	−7.46 (−15.51 to 0.58)	18.65 (−7.81 to 45.12)	−16.92 (−35.34 to 1.51)	80.8, 7.7	−3.91 (−12.54 to 4.73)	−12.46 (−18.91 to −6.02)
FES	−8.34 (−14.31 to −2.37)	−3.55 (−11.40 to 4.29)	22.56 (−3.84 to 48.96)	−13.01 (−31.35 to 5.33)	3.91 (−4.73 to 12.54)	66.0, 1.5	−8.55 (−14.75 to −2.36)
NMES	0.21 (−14.42 to 1.85)	5.00 (0.18 to 9.82)	31.11 (5.34 to 56.89)	−4.46 (−21.87 to 12.96)	12.46 (6.02 to 18.91)	8.55 (2.36 to 14.75)	19.3, 0.0

*Note*: Results in the off‐diagonal cells are the mean difference and 95% confidence intervals of the BI from the network meta‐analysis. Each diagonal cell contains the surface under the cumulative ranking and probability of being the best treatment of each intervention.

Abbreviations: ADL, activities of daily living; BI, Barthel Index; FES, functional electrical stimulation; NMES, neuromuscular electrical stimulation; Rehab, conventional rehabilitation; rPMS, repetitive electrical magnetic stimulation; TMS, transcranial magnetic stimulation.

Sensitivity analysis in the subgroup of studies with acute/subacute stroke showed that NMES+TMS was also in the top rank, with an USMD of 30.9 (95% CI, 5.17–56.62) relative to conventional rehabilitation. rPMS and TMS came in the second and third ranks in improving BI, with significant USMDs of 21.65 (95% CI, 7.17–36.11) and 12.24 (95% CI, 6.01–18.47), respectively, see Appendix 10A,B in Data S1.

The major confidence rating of each pairwise comparison assessed with the CINeMA framework was very low due to major concerns predominantly in the incoherence domain and some concerns in the within‐study bias, imprecision, and heterogeneity domains, see Appendix 9 in Data S1.

### Spasticity

Fifteen studies^24^, ^26^, ^28^, ^30^, ^31^, ^42^, ^51^, ^52^, ^59^, ^60^, ^62^, ^63^, ^64^, ^65^, ^66^ reported MAS at the end of the intervention course, and three studies^30^, ^63^, ^64^ reported at 1–3 months thereafter.

Pairwise meta‐analysis was performed on the end‐of‐intervention MAS in three comparisons, that is, NMES, FES, and TMS versus conventional rehabilitation; only the TMS versus conventional rehabilitation comparison was able to be performed on the 1–3‐month postintervention MAS. The pooled USMDs were nonsignificant in all comparisons, see Appendix 6 in Data S1.

Fifteen studies involving 683 patients assessed with MAS at the end of intervention course were pooled by applying a consistency network meta‐analysis model, including eight pairwise comparisons among six interventions; see Figure 2. All interventions showed no significant differences in spasticity compared to conventional rehabilitation, as shown in Table 4. All subgroup analyses also showed no significant differences; see Appendix 11A–D in Data S1.

**TABLE 4:** Multiple treatment comparisons of interventions on spasticity measured by MAS at the end of intervention courses.

Reference treatment	Unstandardized mean difference					
	Rehab	NMES+TMS	rPMS	TMS	FES	NMES
Rehab	38.5, 0.5	0.14 (−0.45,0.73)	−0.51 (−1.40,0.38)	−0.02 (−0.36,0.33)	−0.03 (−0.67,0.61)	−0.20 (−0.64,0.24)
NMES+TMS	−0.14 (−0.73,0.45)	25.9, 3.0	−0.65 (−1.72,0.42)	−0.16 (−0.76,0.44)	−0.17 (−1.04,0.70)	−0.34 (−1.00,0.32)
rPMS	0.51 (−0.38,1.40)	0.65 (−0.42,1.72)	80.2, 62.0	0.49 (−0.46,1.45)	0.48 (−0.62,1.58)	0.31 (−0.68,1.30)
TMS	0.02 (−0.33,0.36)	0.16 (−0.44,0.76)	−0.49 (−1.45,0.46)	42.4 to 4.0	−0.01 (−0.74,0.72)	−0.19 (−0.70,0.33)
FES	0.03 (−0.61,0.67)	0.17 (−0.70,1.04)	−0.48 (−1.58,0.62)	0.01 (−0.72,0.74)	45.0,11.3	−0.17 (−0.95,0.61)
NMES	0.20 (−0.24,0.64)	0.34 (−0.32,1.00)	−0.31 (−1.30,0.68)	0.19 (−0.33,0.70)	0.17 (−0.61,0.95)	67.9, 19.2

*Note*: Results in the off‐diagonal cells are the mean difference and 95% confidence intervals of the MAS from the network meta‐analysis. Each diagonal cell contains the surface under the cumulative ranking and probability of being the best treatment of each intervention.

Abbreviations: FES, functional electrical stimulation; MAS, Modified Ashworth Scale; NMES, neuromuscular electrical stimulation; Rehab, conventional rehabilitation; rPMS, repetitive electrical magnetic stimulation; TMS, transcranial magnetic stimulation.

The overall confidence rating was low to very low due to major concerns predominantly in the imprecision domain and some concerns mainly in the within‐study bias, imprecision, and heterogeneity domains, see Appendix 9 in Data S1.

### Publication bias

The comparison‐adjusted funnel plots for all outcomes are shown in Appendix 12 in Data S1. The comparison‐adjusted funnel plot was considered asymmetric for all outcomes. This might be caused by heterogeneity in the TMS versus conventional rehabilitation comparison in all three outcomes and NMES versus conventional rehabilitation comparisons in the FMA and MAS outcomes.

## DISCUSSION

Multiple interventions are currently applied for poststroke rehabilitation to improve upper extremity functions, ADL, and spasticity. For the upper extremity outcome, our pairwise meta‐analysis showed benefit of NMES, FES, and TMS when compared to conventional rehabilitation. Our results support the previous systematic reviews and meta‐analyses, which found these interventions (NMES,^67^, ^68^ FES,^69^ and TMS^70^, ^71^, ^72^, ^73^, ^74^, ^75^) could improve upper extremity functions in patients after stroke. Although there were insufficient data to draw conclusions on the use of single intervention rPMS, rPMS combined with NMES may be the best intervention for improving FMA, especially in acute/subacute stroke and in patients with severe stroke with low baseline FMA. This suggests timely rehabilitation influences the benefit of intervention in acute/subacute stroke patients, compared to chronic stroke patients.^76^ In addition, high‐frequency TMS (≥5 Hz) trended toward greater FMA improvement than low‐frequency (≤1 Hz).

Nevertheless, rPMS machines are quite expensive and may not be available in small hospitals. NMES is a less expensive intervention than rPMS and was ranked second, which could improve upper extremity function. A rPMS or TMS machine can cost US $15,000–75,000, whereas the cost of a NMES or FES machine is approximately $100–3500. Furthermore, an interesting finding from our results is that NMES alone was more efficacious than NMES+TMS in improving FMA; this may be due to the varied types of TMS used. Hiragami et al.^77^ reported that a score change in FMA of 12.4 points was clinically meaningful in stroke patients. Our results found that only the patients who received NMES+rPMS reached this minimal clinically important difference.

For the ADL outcome, our pairwise meta‐analysis showed benefits for TMS relative to conventional rehabilitation, which was similar to the findings from a previous systematic review and meta‐analysis.^72^ However, our results showed no benefit from NMES in improving ADL, contrary to a recent systematic review and meta‐analysis.^78^ The difference may be due to discrepancies in the number of studies and method of calculation. In our review, we used the USMD of BI, but the previous review^78^ used the SMDs of many scores. Findings from network meta‐analysis indicated that NMES+TMS might be the best combined intervention, whereas TMS was the best single intervention. The subgroup analysis in acute/subacute stroke patients also showed that NMES+TMS was the best combined intervention, but the best single intervention was rPMS. Thus, rPMS may be beneficial in the acute/subacute stroke group. rPMS can recruit peripheral afferents, potentially influencing cerebral activation and neuroplasticity that may help improve motor control in stroke patients.^79^, ^80^

For the spasticity outcome, neither pairwise meta‐analysis nor network meta‐analysis showed benefit from the use of NMES, FES, and TMS when compared to conventional rehabilitation, a finding similar to those from previous systematic reviews and meta‐analyses.^81^, ^82^ Although rPMS was the first‐ranked intervention in the network meta‐analysis, it was not significantly different compared to other interventions. Therefore, the treatment of choice for spasticity may be oral or injectable antispasticity drugs.^83^

The treatment rankings from network meta‐analysis of the upper extremity functions and ADL outcomes were different in that the top‐ranked intervention was NMES+rPMS for FMA but NMES+TMS for BI. FMA is a clinician‐reported measurement, but BI is patient reported. Therefore, the results from FMA may be more objective. To explore the discrepancy between these measures in the same patients, a sensitivity analysis was attempted by performing network meta‐analysis of only the studies that reported both outcomes, but NMES+rPMS and NMES+TMS fell into two disconnected loops in the network meta‐analysis. Therefore, we could not assess the comparability between the results of both outcomes. However, the results of a pairwise comparison between NMES+rPMS and NMES based on a single study^10^ showed the same direction of greater improvement in both FMA and BI in the NMES+rPMS group than the NMES group. Thus, NMES+rPMS may have benefits in improving not only upper extremity functions but also ADL.

### Strengths and limitations

This study is the first systematic review and network meta‐analysis to evaluate the effects of rPMS along with other interventions used in poststroke rehabilitation. However, it has some limitations. First, the number of studies for some comparisons, particularly those involving rPMS, was limited, potentially impacting the precision of the estimated treatment effects. Therefore, studies were included only if >50% of participants were stroke patients. To assess the robustness of our findings, a sensitivity analysis was conducted by excluding two studies with assorted patient types: El Nahas et al.^62^ (64% stroke patients, 36% limb spasticity) and Krewer et al.^41^ (95% stroke patients, 5% traumatic brain injury). This exclusion did not alter the intervention ranking, see Appendix 13 in Data S1.

Because the typical indications of rPMS are musculoskeletal and neurogenic pain,^84^ treatment with rPMS is currently new in patients after stroke and not widely used. More high‐quality RCTs are needed to confirm the effects of rPMS in poststroke patients. We also found non‐RCTs^12^, ^85^ that were ineligible in our systematic review and network meta‐analysis but which suggested that adding rPMS to conventional rehabilitation had a beneficial effect on upper extremity function. Moreover, we found an ongoing RCT^86^ of rPMS on upper extremity function that could be added to the meta‐analysis in the future. Second, consistency in some network meta‐analysis models could not be achieved by removing studies with characteristics dissimilar to the others included; inconsistency models were applied instead. Lastly, there was evidence of publication bias in the comparison of NMES versus conventional rehabilitation on the spasticity outcome; readers should interpret or apply these results with caution.

## CONCLUSION

Network meta‐analysis suggests that NMES combined with rPMS may be the most effective intervention for improving upper extremity function after stroke, although few studies have investigated this combination. NMES alone may be a cost‐effective alternative, and NMES plus TMS may offer the greatest improvements in ADL. However, evidence supporting the efficacy of these interventions in reducing spasticity is insufficient. The low certainty of these conclusions, due to inconsistencies in the network meta‐analysis, necessitates further well‐designed RCTs.

## DISCLOSURE

All authors declare that they have no conflicts of interest.

This journal‐based CME activity is designated for 1.0 *AMA PRA Category 1 Credit* ^TM^. Effective January 2024, learners are no longer required to correctly answer a multiple‐choice question to receive CME credit. Completion of an evaluation is required, which can be accessed using this link, https://onlinelearning.aapmr.org/. This activity is FREE to AAPM&R members and available to nonmembers for a nominal fee. CME is available for 3 years after publication date. For assistance with claiming CME for this activity, please contact (847) 737–6000. All financial disclosures and CME information related to this article can be found on the Online Learning Portal (https://onlinelearning.aapmr.org/) prior to accessing the activity.

## Supporting information


**Data S1.** Supporting Information.
