# Supplementary material for: Effects of electrical and magnetic stimulation on upper extremity function after stroke: A systematic review and network meta‐analysis
Source: PM R. 2025 May 21;17(8):978–93. doi: 10.1002/pmrj.13356 (PMC12345400; doi:10.1002/pmrj.13356)
Supplement: Supplementary file 1 — Data S1. Supporting Information. [file PMRJ-17-978-s001.docx]

**Appendix 1:** Search strategies

**A)** Search strategy in MEDLINE via PubMed

| **Domain** | **Subdomain** | **Search number** | **Query** |
| --- | --- | --- | --- |
| **P** | P1 stroke | #1 | Search: stroke |
|  |  | #2 | Search: hemiparesis |
|  |  | #3 | Search: hemiplegia |
|  |  | #4 | Search: “cerebrovascular accident” |
|  |  | #5 | Search: #1 OR #2 OR #3 OR #4 |
| **I** | I1 rPMS | #6 | Search: “repetitive peripheral magnetic stimulation” |
|  |  | #7 | Search: “peripheral magnetic stimulation” |
|  |  | #8 | #6 OR #7 |
|  |  | #9 | Search: “peripheral repetitive magnetic stimulation” |
|  |  | #10 | Search: #7 OR #9 |
|  | I2 TMS | #11 | Search: “transcranial magnetic stimulation” |
|  |  | #12 | Search: “theta burst stimulation” |
|  |  | #13 | Search: “Transcranial Magnetic Stimulation”[MeSH Terms] |
|  |  | #14 | Search: #11 OR #13 |
|  |  | #15 | Search: #11 OR #12 |
|  | I3 ES | #16 | Search: “electrical stimulation” |
|  |  | #17 | Search: “neuromuscular electrical stimulation” |
|  |  | #18 | Search: #16 OR #17 |
|  |  | #19 | Search: “functional electrical stimulation” |
|  |  | #20 | Search: #16 OR #19 |
|  |  | #21 | Search**:** “Electric Stimulation”[MeSH Terms] |
|  |  | #22 | Search: “Electric Stimulation Therapy”[MeSH Terms] |
|  |  | #23 | Search: #21 OR #22 |
|  |  | #24 | Search: #16 OR #21 OR #22 |
|  | I1+2+3 | #25 | Search #10 OR #15 OR #23 |
| **O** | O1 UE function | #26 | Search: “upper limb” |
|  |  | #27 | Search: “upper extremity” |
|  |  | #28 | Search: “upper extremities” |
|  |  | #29 | Search: #27 OR #28 |
|  |  | #30 | Search: “upper extremit*” |
|  |  | #31 | Search: shoulder |
|  |  | #32 | Search: arm |
|  |  | #33 | Search: elbow |
|  |  | #34 | Search: forearm |
|  |  | #35 | Search: wrist |
|  |  | #36 | Search: hand |
|  |  | #37 | Search: finger |
|  |  | #38 | Search: thumb |
|  |  | #39 | Search: digit |
|  |  | #40 | Search: #26 OR #30 OR #31 OR #32 OR #33 OR #34 OR #35 OR #36 OR #37 OR #38 OR #39 |
|  |  | #41 | Search: function |
|  |  | #42 | Search: recovery |
|  |  | #43 | Search: strength |
|  |  | #44 | Search: power |
|  |  | #45 | Search: mobility |

Abbreviations: P, patient; I, intervention; O, outcome; UE, upper extremity.

**A)** Search strategy in MEDLINE via PubMed (Cont.)

| **Domain** | **Subdomain** | **Search number** | **Query** |
| --- | --- | --- | --- |
|  |  | #46 | Search: #41 OR #42 OR #43 OR #44 OR #45 |
|  |  | #47 | Search: #40 AND #46 |
|  |  | #48 | Search: **“Fugl Meyer Score”** |
|  |  | #49 | Search: **“Fugl Meyer”** |
|  |  | #50 | Search: #48 OR #49 |
|  |  | #51 | Search: **“Action research arm test”** |
|  |  | #52 | Search: “Action research arm” |
|  |  | #53 | Search: #51 OR #52 |
|  |  | #54 | Search: **“Wolf motor function test”** |
|  |  | #55 | Search: “Wolf motor function” |
|  |  | #56 | Search: #54 OR #55 |
|  |  | #57 | Search: “Jebsen taylor hand function test” |
|  |  | #58 | Search: “Jebsen taylor hand function” |
|  |  | #59 | Search: #57 OR #58 |
|  |  | #60 | Search: #47 OR #49 OR #52 OR #55 OR #58 |
|  | O2 ADL | #61 | Search: “activities of daily living” |
|  |  | #62 | Search: “activity of daily living” |
|  |  | #63 | Search: #61 OR #62 |
|  |  | #64 | Search: “activities of daily living”[MeSH Terms] |
|  |  | #65 | Search: #61 OR #62 OR #64 |
|  |  | #66 | Search: “daily living activity” |
|  |  | #67 | Search: “daily living activities” |
|  |  | #68 | Search: “daily living activit*” |
|  |  | #69 | Search: #66 OR #67 OR #68 |
|  |  | #70 | Search: “daily activity” |
|  |  | #71 | Search: “daily activities” |
|  |  | #72 | Search: “daily activit*” |
|  |  | #73 | Search: #70 OR #71 OR #72 |
|  |  | #74 | Search: “Barthel index” |
|  |  | #75 | Search: “modified Barthel index” |
|  |  | #76 | Search: #74 OR #75 |
|  |  | #77 | Search: “functional independence measure” |
|  |  | #78 | Search: #65 OR #68 OR #72 OR #74 OR #77 |
|  | O3 spastic | #79 | Search: spastic |
|  |  | #80 | Search: spasticity |
|  |  | #81 | Search: spastic* |
|  |  | #82 | Search: #79 OR #80 OR #81 |
|  |  | #83 | Search: “tardieu scale” |
|  |  | #84 | Search: “modified tardieu scale” |
|  |  | #85 | Search: #83 OR #84 |
|  |  | #86 | Search: “modified ashworth scale” |
|  |  | #87 | Search: #82 OR #83 OR #86 |
|  | O1+2+3 | #88 | Search: #60 OR #78 OR #87 |

Abbreviation: O, outcome.

**A)** Search strategy in MEDLINE via PubMed (Cont.)

| **Domain** | **Subdomain** | **Search number** | **Query** |
| --- | --- | --- | --- |
| **S** | RCT | #89 | Search: “randomized controlled trial” |
|  |  | #90 | Search: “randomised controlled trial” |
|  |  | #91 | Search: #89 OR #90 |
|  |  | #92 | Search: “randomized clinical study” |
|  |  | #93 | Search: “randomised clinical study” |
|  |  | #94 | Search: #92 OR #93 |
|  |  | #95 | Search: “randomized trial” |
|  |  | #96 | Search: “randomised trial” |
|  |  | #97 | Search: #95 OR #96 |
|  |  | #98 | Search: “randomized study” |
|  |  | #99 | Search: “randomised study” |
|  |  | #100 | Search: #98 OR #99 |
|  |  | #101 | Search: #91 OR #94 OR #97 OR #100 |
|  |  | #102 | Search: “Randomized Controlled Trial” [Publication Type] |
|  |  | #103 | Search: #101 OR #102 |
|  |  | #104 | Search: “clinical trial” |
|  |  | #105 | Search: “Clinical Trial” [Publication Type] |
|  |  | #106 | Search: #104 OR #105 |
|  |  | #107 | Search: “cross over trial” |
|  |  | #108 | Search: “cross-over trial” |
|  |  | #109 | Search: “crossover trial” |
|  |  | #110 | Search: #107 OR #109 |
|  |  | #111 | Search: “crossover study” |
|  |  | #112 | Search: “cross over study” |
|  |  | #113 | Search: #111 OR #112 |
|  |  | #114 | Search: “cross over design” |
|  |  | #115 | Search: “crossover design” |
|  |  | #116 | Search: #114 OR #115 |
|  |  | #117 | Search: #110 OR #113 OR #116 |
|  |  | #118 | Search: “Cross-Over Studies”[Mesh] |
|  |  | #119 | Search: #117 OR #118 |
|  |  | #120 | Search: #103 OR #106 OR #119 |
| **P+I** |  |  | Search: #5 AND #25 |
| **P+I+O** |  |  | Search: #5 AND #25 AND #88 |
| **P+I+O+S** |  |  | Search: #5 AND #25 AND #88 AND #120 |

Abbreviations: P, patient; I, intervention; O, outcome; S, study design.

**B)** Search strategy in Scopus

| Domain | Subdomain | Search Number | Query |
| --- | --- | --- | --- |
| P | P1 stroke | #1 | TITLE-ABS-KEY ( stroke ) |
|  |  | #2 | TITLE-ABS-KEY ( “cerebrovascular accident” ) |
|  |  | #3 | TITLE-ABS-KEY ( hemiparesis ) |
|  |  | #4 | TITLE-ABS-KEY ( hemiplegia ) |
|  |  | #5 | #1 OR #2 OR #3 OR #4 |
| I | I1 rPMS | #6 | TITLE-ABS-KEY ( “repetitive peripheral magnetic stimulation” ) |
|  |  | #7 | TITLE-ABS-KEY ( “peripheral magnetic stimulation” ) |
|  |  | #8 | #6 OR #7 |
|  |  | #9 | TITLE-ABS-KEY ( “peripheral repetitive magnetic stimulation” ) |
|  |  | #10 | #7 OR #9 |
|  | I2 TMS | #11 | TITLE-ABS-KEY ( “transcranial magnetic stimulation” ) |
|  |  | #12 | TITLE-ABS-KEY ( “theta burst stimulation” ) |
|  |  | #13 | #11 OR #12 |
|  | I3 ES | #14 | TITLE-ABS-KEY ( “electrical stimulation” ) |
|  |  | #15 | TITLE-ABS-KEY ( “neuromuscular electrical stimulation” ) |
|  |  | #16 | #14 OR #15 |
|  |  | #17 | TITLE-ABS-KEY ( “functional electrical stimulation” ) |
|  |  | #18 | #14 OR #17 |
|  | I1+2+3 | #19 | #10 OR #13 OR #14 |
| O | O1 UE function | #20 | TITLE-ABS-KEY ( “upper limb” ) |
|  |  | #21 | TITLE-ABS-KEY ( “upper extremity” ) |
|  |  | #22 | TITLE-ABS-KEY ( “upper extremities” ) |
|  |  | #23 | TITLE-ABS-KEY ( shoulder ) |
|  |  | #24 | TITLE-ABS-KEY ( arm ) |
|  |  | #25 | TITLE-ABS-KEY ( elbow ) |
|  |  | #26 | TITLE-ABS-KEY ( forearm ) |
|  |  | #27 | TITLE-ABS-KEY ( wrist ) |
|  |  | #28 | TITLE-ABS-KEY ( hand ) |
|  |  | #29 | TITLE-ABS-KEY ( finger ) |
|  |  | #30 | TITLE-ABS-KEY ( thumb ) |
|  |  | #31 | TITLE-ABS-KEY ( digit ) |
|  |  | #32 | #20 OR #21 OR #23 OR #24 OR #25 OR #26 OR #27 OR #28 OR #29 OR #30 OR #31 |
|  |  | #33 | TITLE-ABS-KEY ( function ) |

Abbreviations: P, patient; I, intervention; O, outcome; UE, upper extremity.

**B)** Search strategy in Scopus (Cont.)

| Domain | Subdomain | Search Number | Query |
| --- | --- | --- | --- |
|  |  | #34 | TITLE-ABS-KEY ( recovery ) |
|  |  | #35 | TITLE-ABS-KEY ( strength ) |
|  |  | #36 | TITLE-ABS-KEY ( power ) |
|  |  | #37 | TITLE-ABS-KEY ( mobility ) |
|  |  | #38 | #33 OR #34 OR #35 OR #36 OR #37 |
|  |  | #39 | #32 AND #38 |
|  |  | #40 | TITLE-ABS-KEY ( **“Fugl Meyer Score”** ) |
|  |  | #41 | TITLE-ABS-KEY ( “**Fugl Meyer”** ) |
|  |  | #42 | #40 OR #41 |
|  |  | #43 | TITLE-ABS-KEY ( **“Action research arm test”** ) |
|  |  | #44 | TITLE-ABS-KEY ( **“Action research arm”** ) |
|  |  | #45 | #43 OR #44 |
|  |  | #46 | TITLE-ABS-KEY ( **“Wolf motor function test” )** |
|  |  | #47 | TITLE-ABS-KEY ( **“Wolf motor function” )** |
|  |  | #48 | #46 OR #47 |
|  |  | #49 | TITLE-ABS-KEY ( “Jebsen taylor hand function test” ) |
|  |  | #50 | TITLE-ABS-KEY ( “Jebsen taylor hand function” ) |
|  |  | #51 | #49 OR #50 |
|  |  | #52 | #39 OR #41 OR #44 OR #47 OR #50 |
|  | O2 ADL | #53 | TITLE-ABS-KEY ( “activities of daily living” ) |
|  |  | #54 | TITLE-ABS-KEY ( “activity of daily living” ) |
|  |  | #55 | TITLE-ABS-KEY ( “daily living activity” ) |
|  |  | #56 | TITLE-ABS-KEY ( “daily living activities” ) |
|  |  | #57 | TITLE-ABS-KEY ( “daily activity” ) |
|  |  | #58 | TITLE-ABS-KEY ( “daily activities” ) |
|  |  | #59 | TITLE-ABS-KEY ( “Barthel index” ) |
|  |  | #60 | TITLE-ABS-KEY ( “modified Barthel index” ) |
|  |  | #61 | #59 OR #60 |
|  |  | #62 | TITLE-ABS-KEY ( “functional independence measure” ) |
|  |  | #63 | #53 OR #55 OR #57 OR #59 OR #62 |
|  | O3 spastic | #64 | TITLE-ABS-KEY ( spastic ) |
|  |  | #65 | TITLE-ABS-KEY ( spasticity ) |
|  |  | #66 | TITLE-ABS-KEY ( spastic* ) |
|  |  | #67 | #64 OR #65 OR #66 |
|  |  | #68 | TITLE-ABS-KEY ( “tardieu scale” ) |
|  |  | #69 | TITLE-ABS-KEY ( “modified tardieu scale” ) |
|  |  | #70 | #68 OR #69 |
|  |  | #71 | TITLE-ABS-KEY ( “modified ashworth scale” ) |
|  |  | #72 | #66 OR #68 OR #71 |
|  | O1+2+3 | #73 | #52 OR #63 OR #72 |

Abbreviation: O, outcome.

**B)** Search strategy in Scopus (Cont.)

| Domain | Subdomain | Search Number | Query |
| --- | --- | --- | --- |
|  |  | #71 | TITLE-ABS-KEY ( “modified ashworth scale” ) |
|  |  | #72 | #66 OR #68 OR #71 |
|  | O1+2+3 | #73 | #52 OR #63 OR #72 |
| S | RCT | #74 | TITLE-ABS-KEY ( “randomized controlled trial” ) |
|  |  | #75 | TITLE-ABS-KEY ( “random* control* trial” ) |
|  |  | #76 | #74 OR #75 |
|  |  | #77 | TITLE-ABS-KEY ( “randomized clinical study” ) |
|  |  | #78 | TITLE-ABS-KEY ( “randomized trial” ) |
|  |  | #79 | TITLE-ABS-KEY ( “randomized study” ) |
|  |  | #80 | TITLE-ABS-KEY ( “clinical trial” ) |
|  |  | #81 | TITLE-ABS-KEY ( “cross over trial” ) |
|  |  | #82 | TITLE-ABS-KEY ( “crossover trial” ) |
|  |  | #83 | #81 OR #82 |
|  |  | #84 | TITLE-ABS-KEY ( “crossover study” ) |
|  |  | #85 | TITLE-ABS-KEY ( “cross over study” ) |
|  |  | #86 | #84 OR #85 |
|  |  | #87 | TITLE-ABS-KEY ( “cross over design” ) |
|  |  | #88 | TITLE-ABS-KEY ( “crossover design” ) |
|  |  | #89 | #87 OR #88 |
|  |  | #90 | #75 OR #77 OR #78 OR #79 OR #80 OR #81 OR #82 OR #84 OR #85 OR #87 OR #88 |
| P+I |  |  | #5 AND #19 |
| P+I+O |  |  | #5 AND #19 AND #73 |
| P+I+O+S |  |  | #5 AND #19 AND #58 AND #90 |

Abbreviations: P, patient; I, intervention; O, outcome; S, study design.

**C)** Search strategy in Google Scholar

| **Domain** | **Subdomain** | **Search** | **Query** |
| --- | --- | --- | --- |
| **I** | I1 rPMS | #1 | allintitle: “repetitive peripheral magnetic stimulation” |
|  | I2 TMS | #2 | allintitle: “transcranial magnetic stimulation” |
|  | I3 ES | #3 | allintitle: “neuromuscular electrical stimulation” OR “functional electrical stimulation” |
|  | I1 + 2 + 3 | #4 | allintitle: “repetitive peripheral magnetic stimulation” OR “transcranial magnetic stimulation” OR “neuromuscular electrical stimulation” OR “functional electrical stimulation” |
| **P + I** |  | #5 | allintitle: stroke “repetitive peripheral magnetic stimulation” OR “transcranial magnetic stimulation” OR “neuromuscular electrical stimulation” OR “functional electrical stimulation” |
| **I + O1** | O1 UE function | #6 | allintitle: “upper limb function” “repetitive peripheral magnetic stimulation” OR “transcranial magnetic stimulation” OR “neuromuscular electrical stimulation” OR “functional electrical stimulation” |
|  |  | #7 | allintitle: “repetitive peripheral magnetic stimulation” OR “transcranial magnetic stimulation” OR “neuromuscular electrical stimulation” OR “functional electrical stimulation” “upper extremity function” |
| **I + O2** | O2 ADL | #8 | allintitle: “activities of daily living” “repetitive peripheral magnetic stimulation” OR “transcranial magnetic stimulation” OR “neuromuscular electrical stimulation” OR “functional electrical stimulation” |
|  |  | #9 | allintitle: “repetitive peripheral magnetic stimulation” OR “transcranial magnetic stimulation” OR “neuromuscular electrical stimulation” OR “functional electrical stimulation” “daily living activities” |
|  |  | #10 | allintitle: “repetitive peripheral magnetic stimulation” OR “transcranial magnetic stimulation” OR “neuromuscular electrical stimulation” OR “functional electrical stimulation” “daily activities” |
| **I + O3** | O3 Spastic | #11 | allintitle: spastic “repetitive peripheral magnetic stimulation” OR “transcranial magnetic stimulation” OR “neuromuscular electrical stimulation” OR “functional electrical stimulation” |
|  |  | #12 | allintitle: spasticity “repetitive peripheral magnetic stimulation” OR “transcranial magnetic stimulation” OR “neuromuscular electrical stimulation” OR “functional electrical stimulation” |

Abbreviations: P, patient; I, intervention; O, outcome; S, study design; UE, upper extremity.

**D)** Search strategy in the Cochrane Central Register of Controlled Clinical Trials

| **Domain** | **Subdomain** | **Search Number** | **Query** |
| --- | --- | --- | --- |
| **P** | P1 stroke | #1 | Search: stroke |
|  |  | #2 | Search: hemiparesis |
|  |  | #3 | Search: hemiplegia |
|  |  | #4 | Search: “cerebrovascular accident” |
|  |  | #5 | Search: #1 OR #2 OR #3 OR #4 |
| **I** | I1 rPMS | #6 | Search: “repetitive peripheral magnetic stimulation” |
|  |  | #7 | Search: “peripheral magnetic stimulation” |
|  |  | #8 | #6 OR #7 |
|  |  | #9 | Search: “peripheral repetitive magnetic stimulation” |
|  |  | #10 | Search: #7 OR #9 |
|  | I2 TMS | #11 | Search: “transcranial magnetic stimulation” |
|  |  | #12 | Search: “theta burst stimulation” |
|  |  | #13 | Search: MeSH descriptor: [Transcranial Magnetic Stimulation] explode all trees |
|  |  | #14 | Search: #11 OR #13 |
|  |  | #15 | Search: #11 OR #12 |
|  | I3 ES | #16 | Search: “electrical stimulation” |
|  |  | #17 | Search: “neuromuscular electrical stimulation” |
|  |  | #18 | Search: #16 OR #17 |
|  |  | #19 | Search: “functional electrical stimulation” |
|  |  | #20 | Search: #16 OR #19 |
|  |  | #21 | Search**:** MeSH descriptor: [Electric Stimulation] explode all trees |
|  |  | #22 | Search: MeSH descriptor: [Electric Stimulation Therapy] explode all trees |
|  |  | #23 | Search: #21 OR #22 |
|  |  | #24 | Search: #16 OR #21 OR #22 |
|  | I1+2+3 | #25 | Search #10 OR #15 OR #23 |
| **O** | O1 UE function | #26 | Search: “upper limb” |
|  |  | #27 | Search: “upper extremity” |
|  |  | #28 | Search: “upper extremities” |
|  |  | #29 | Search: #27 OR #28 |
|  |  | #30 | Search: “upper extremit*” |
|  |  | #31 | Search: shoulder |
|  |  | #32 | Search: arm |
|  |  | #33 | Search: elbow |
|  |  | #34 | Search: forearm |
|  |  | #35 | Search: wrist |
|  |  | #36 | Search: hand |
|  |  | #37 | Search: finger |
|  |  | #38 | Search: thumb |
|  |  | #39 | Search: digit |

Abbreviations: P, patient; I, intervention; O, outcome; UE, upper extremity.

**D)** Search strategy in the Cochrane Central Register of Controlled Clinical Trials (Cont.)

| **Domain** | **Subdomain** | **Search Number** | **Query** |
| --- | --- | --- | --- |
|  |  | #40 | Search: #26 OR #27 OR #28 OR #30 OR #31 OR #32 OR #33 OR #34 OR #35 OR #36 OR #37 OR #38 OR #39 |
|  |  | #41 | Search: function |
|  |  | #42 | Search: recovery |
|  |  | #43 | Search: strength |
|  |  | #44 | Search: power |
|  |  | #45 | Search: mobility |
|  |  | #46 | Search: #41 OR #42 OR #43 OR #44 OR #45 |
|  |  | #47 | Search: #40 AND #46 |
|  |  | #48 | Search: “**Fugl Meyer Score”** |
|  |  | #49 | Search: “**Fugl Meyer”** |
|  |  | #50 | Search: #48 OR #49 |
|  |  | #51 | Search: “**Action research arm test”** |
|  |  | #52 | Search: “Action research arm” |
|  |  | #53 | Search: #51 OR #52 |
|  |  | #54 | Search: “**Wolf motor function test”** |
|  |  | #55 | Search: “Wolf motor function” |
|  |  | #56 | Search: #54 OR #55 |
|  |  | #57 | Search: “Jebsen taylor hand function test” |
|  |  | #58 | Search: “Jebsen taylor hand function” |
|  |  | #59 | Search: #57 OR #58 |
|  |  | #60 | Search: #47 OR #49 OR #52 OR #55 OR #58 |
|  | O2 ADL | #61 | Search: “activities of daily living” |
|  |  | #62 | Search: “activity of daily living” |
|  |  | #63 | Search: #61 OR #62 |
|  |  | #64 | Search: MeSH descriptor: [Activities of Daily Living] explode all trees |
|  |  | #65 | Search: #61 OR #62 OR #64 |
|  |  | #66 | Search: “daily living activity” |
|  |  | #67 | Search: “daily living activities” |
|  |  | #68 | Search: “daily living activit*” |
|  |  | #69 | Search: #66 OR #67 |
|  |  | #70 | Search: “daily activity” |
|  |  | #71 | Search: “daily activities” |
|  |  | #72 | Search: “daily activit*” |
|  |  | #73 | Search: #70 OR #71 |
|  |  | #74 | Search: “Barthel index” |
|  |  | #75 | Search: “modified Barthel index” |
|  |  | #76 | Search: #74 OR #75 |
|  |  | #77 | Search: “functional independence measure” |
|  |  | #78 | Search: #65 OR #69 OR #73 OR #76 OR #77 |
|  | O3 spastic | #79 | Search: spastic |
|  |  | #80 | Search: spasticity |
|  |  | #81 | Search: spastic* |

Abbreviation: O, outcome.

**D)** Search strategy in the Cochrane Central Register of Controlled Clinical Trials (Cont.)

| Domain | Subdomain | Search Number | Query |
| --- | --- | --- | --- |
|  |  | #82 | Search: #79 OR #80 OR #81 |
|  |  | #83 | Search: “tardieu scale” |
|  |  | #84 | Search: “modified tardieu scale” |
|  |  | #85 | Search: #83 OR #84 |
|  |  | #86 | Search: “modified ashworth scale” |
|  |  | #87 | Search: #82 OR #83 OR #86 |
|  | O1+2+3 | #88 | Search: #60 OR #78 OR #87 |
| P+I |  |  | Search: #5 AND #25 |
| P+I+O |  |  | Search: #5 AND #25 AND #88 |

Abbreviations: P, patient; I, intervention; O, outcome.

**E)** Search strategy in the Physiotherapy Evidence Database

“peripheral magnetic stimulation” AND stroke

“neuromuscular electrical stimulation” AND stroke

“functional electrical stimulation” AND stroke

“transcranial magnetic stimulation” AND stroke

**Appendix 2:** Data extraction form

**Part I: General information**

1. Date of extraction 🞎🞎 / 🞎🞎 /20🞎🞎 (DD/MM/YYYY A.D.)
2. Reviewer 🞎1. AK 🞎2. MS
3. Study ID 🞎🞎🞎🞎
4. First author ____________________________
5. Corresponding author ____________________________
6. Publication year 🞎🞎🞎🞎
7. E-mail ____________________________

**Part II: Study characteristics**

1. Country ____________________
2. Study design 🞎1. Parallel-RCT 🞎2. Cross-over trial
3. Setting 🞎1. In patient 🞎2. Outpatient 🞎3. Home base
    🞎4. Other (specify) ____________________
4. Longest Follow up time 🞎 _____ minute

🞎 _____ day

🞎 _____ month

🞎 _____ year

**Part III: General characteristic of participants**

| **Characteristic** | 🞎1.rPMS 🞎2.TMS 🞎3.NMES 🞎4.FES 🞎5.TMS+NMES 🞎6.TMS+FES 🞎7.Sham/Rehab 🞎8.others ______ | | 🞎1.rPMS 🞎2.TMS 🞎3.NMES 🞎4.FES 🞎5.TMS+NMES 🞎6.TMS+FES 🞎7.Sham/Rehab 🞎8.others ______ | | 🞎1.rPMS 🞎2.TMS 🞎3.NMES 🞎4.FES 🞎5.TMS+NMES 🞎6.TMS+FES 🞎7.Sham/Rehab 🞎8.others ______ | | 🞎1.rPMS 🞎2.TMS 🞎3.NMES 🞎4.FES 🞎5.TMS+NMES 🞎6.TMS+FES 🞎7.Sham/Rehab 🞎8.others ______ | | **Total** | |
| --- | --- | --- | --- | --- | --- | --- | --- | --- | --- | --- |
|  | Mean | Median | Mean | Median | Mean | Median | Mean | Median | Mean | Median |
| Age (year) |  |  |  |  |  |  |  |  |  |  |
| Gender: male (%) |  | |  | |  | |  | |  | |
| Stroke patient (%) |  | |  | |  | |  | |  | |
| Time since stroke  (unit _____d, m, y) |  |  |  |  |  |  |  |  |  |  |
| BMI (kg/m^2^) |  |  |  |  |  |  |  |  |  |  |
| Smoking (%) |  |  |  |  |  |  |  |  |  |  |
| Ischemic stroke (%) |  | |  | |  | |  | |  | |
| Hemorrhagic stroke (%) |  | |  | |  | |  | |  | |
| Left hemisphere lesion, right hemiparesis (%) |  | |  | |  | |  | |  | |
| Hemineglect (%) |  | |  | |  | |  | |  | |
| Lesion location |  | |  | |  | |  | |  | |
| Cortical (%) |  | |  | |  | |  | |  | |
| Subcortical (%) |  | |  | |  | |  | |  | |
| Cortical/Subcortical (%) |  | |  | |  | |  | |  | |
| Brain stem (%) |  | |  | |  | |  | |  | |

| **Characteristic** | 🞎1.rPMS 🞎2.TMS 🞎3.NMES 🞎4.FES 🞎5.TMS+NMES 🞎6.TMS+FES 🞎7.Sham/Rehab 🞎8.others ______ | | 🞎1.rPMS 🞎2.TMS 🞎3.NMES 🞎4.FES 🞎5.TMS+NMES 🞎6.TMS+FES 🞎7.Sham/Rehab 🞎8.others ______ | | 🞎1.rPMS 🞎2.TMS 🞎3.NMES 🞎4.FES 🞎5.TMS+NMES 🞎6.TMS+FES 🞎7.Sham/Rehab 🞎8.others ______ | | | 🞎1.rPMS 🞎2.TMS 🞎3.NMES 🞎4.FES 🞎5.TMS+NMES 🞎6.TMS+FES 🞎7.Sham/Rehab 🞎8.others ______ | | **Total** | |
| --- | --- | --- | --- | --- | --- | --- | --- | --- | --- | --- | --- |
|  | Mean | Median | Mean | Median | Mean | | Median | Mean | Median | Mean | Median |
| Co-morbidity |  |  |  |  |  |  | |  |  |  |  |
| Depression (%) |  |  |  |  |  |  | |  |  |  |  |
| Coronary heart disease (%) |  |  |  |  |  |  | |  |  |  |  |
| Diabetes-mellitus (%) |  |  |  |  |  |  | |  |  |  |  |
| Hypertension (%) |  |  |  |  |  |  | |  |  |  |  |
| Upper extremity function |  |  |  |  |  |  | |  |  |  |  |
| FMA |  |  |  |  |  |  | |  |  |  |  |
| ARAT |  |  |  |  |  |  | |  |  |  |  |
| WMFT |  |  |  |  |  |  | |  |  |  |  |
| JTT |  |  |  |  |  |  | |  |  |  |  |
| ________________ |  |  |  |  |  |  | |  |  |  |  |
| ADL |  |  |  |  |  |  | |  |  |  |  |
| BI |  |  |  |  |  |  | |  |  |  |  |
| mBI |  |  |  |  |  |  | |  |  |  |  |
| FIM |  |  |  |  |  |  | |  |  |  |  |
| _______________ |  |  |  |  |  |  | |  |  |  |  |
| Spasticity |  |  |  |  |  |  | |  |  |  |  |
| MAS |  |  |  |  |  |  | |  |  |  |  |
| Tardieu Scale |  |  |  |  |  |  | |  |  |  |  |
| Modified Tardieu Scale |  |  |  |  |  |  | |  |  |  |  |
| _______________ |  |  |  |  |  |  | |  |  |  |  |
| Others |  |  |  |  |  |  | |  |  |  |  |
| NIHSS |  |  |  |  |  |  | |  |  |  |  |
| Brunstrom stage |  |  |  |  |  |  | |  |  |  |  |
| Brunstrom stage ≤ 3 (%) |  |  |  |  |  |  | |  |  |  |  |
| MMSE |  |  |  |  |  |  | |  |  |  |  |
| MoCA |  |  |  |  |  |  | |  |  |  |  |
| Stroke impact scale |  |  |  |  |  |  | |  |  |  |  |
| Modified ranking scale |  |  |  |  |  |  | |  |  |  |  |

**Part IV: Interventions**

1. Number of study arm 🞎2 🞎3 🞎4
2. Intervention

| **Intervention** | **Detail** | |
| --- | --- | --- |
| 🞎 1.rPMS | Subtype: 🞎 rPMS 🞎 piTBS  Stimulation site: _______________  Brand: 🞎 Magstim 🞎 Neurosoft 🞎 MagVenture 🞎MettaMedtech  🞎 other, specify__________________  Coil: 🞎 circular 🞎 figure of eight 🞎 other, specify __________ | |
|  | Intensity: 🞎 __________ % of motor threshold  🞎 __________ % of maximum stimulator output  🞎 other, specify_____________  Total pulses: 🞎🞎🞎🞎  Frequency: ___________ Hz  Number of trains: 🞎🞎🞎🞎  On time: __________ sec  Off time: __________ sec  Duration per session: 🞎🞎🞎 minute, 🞎🞎🞎 sec  Number of sessions per day: 🞎🞎, 🞎 day/week  Total sessions: 🞎🞎🞎 | **If piTBS add**  Number of bursts: 🞎🞎🞎  Intertrain interval:  🞎🞎🞎 ms, 🞎🞎🞎 Hz  Interblock interval: 🞎🞎🞎 s |
| 🞎 2.TMS | Subtype: 🞎 HF-rTMS 🞎 LF-rTMS 🞎 cTBS 🞎 iTBS  Stimulation site: 🞎 affected hemisphere 🞎 unaffected hemisphere  Brand: 🞎 Magstim 🞎 CloudTMS 🞎 MagVenture  🞎 other, specify__________________  Coil: 🞎 circular 🞎 figure of eight 🞎 other, specify __________ | |
|  | Intensity: 🞎 __________ % of motor threshold  🞎 __________ % of maximum stimulator output  🞎 other, specify_____________  Total pulses: 🞎🞎🞎🞎  Frequency: ___________ Hz  Number of trains: 🞎🞎🞎🞎  On time: __________ sec  Off time: __________ sec  Duration per session: 🞎🞎🞎 minute, 🞎🞎🞎 sec  Number of sessions per day: 🞎🞎, 🞎 day/week  Total sessions: 🞎🞎🞎 | **If cTBS or iTBS add**  Number of bursts: 🞎🞎🞎  Intertrain interval:  🞎🞎🞎 ms, 🞎🞎🞎 Hz  Interblock interval: 🞎🞎🞎 s |
| 🞎 3.NMES | Channel: 🞎 single 🞎 dual 🞎 multi EMG trigger: 🞎 Yes 🞎 No  Brand: ____________________  Intensity: 🞎 ________ mA, 🞎 ________ mV, 🞎 other, specify_____________  Pulse Frequency: __________ Hz  Pulse width: __________ µsec  On time: __________ sec  Off time: __________ sec  Ramp up: __________ sec  Ramp down: __________sec  Duration per session (min): 🞎🞎🞎minute  Number of sessions per day: 🞎🞎, 🞎 day/week  Total session: 🞎🞎🞎 | |

| **Intervention** | **Detail** | |
| --- | --- | --- |
| 🞎 4.FES | Channel: 🞎 single 🞎 dual 🞎 multi  Functional activity: _________________  Brand: ____________________  Intensity: 🞎 ________ mA, 🞎 ________ mV, 🞎 other, specify_____________  Pulse Frequency: __________ Hz  Pulse width: __________ µsec  On time: __________ sec  Off time: __________ sec  Ramp up: __________ sec  Ramp down: __________sec  Duration per session (min): 🞎🞎🞎 minute  Number of sessions per day: 🞎🞎, 🞎 day/week  Total session: 🞎🞎🞎 | |
| 🞎 5.TMS + NMES | **TMS**  Subtype: 🞎 HF-rTMS 🞎 LF-rTMS 🞎 cTBS 🞎 iTBS  Stimulation site: 🞎 affected hemisphere 🞎 unaffected hemisphere  Brand: 🞎 Magstim 🞎 CloudTMS 🞎 MagVenture  🞎 other, specify__________________  Coil: 🞎 circular 🞎 figure of eight 🞎 other, specify __________ | |
|  | Intensity: 🞎 __________ % of motor threshold  🞎 __________ % of maximum stimulator output  🞎 other, specify_____________  Total pulses: 🞎🞎🞎🞎  Frequency: ___________ Hz  Number of trains: 🞎🞎🞎🞎  On time: __________ sec  Off time: __________ sec  Duration per session: 🞎🞎🞎 minute, 🞎🞎🞎 sec  Number of sessions per day: 🞎🞎, 🞎 day/week  Total sessions: 🞎🞎🞎 | **If cTBS or iTBS add**  Number of bursts: 🞎🞎🞎  Intertrain interval:  🞎🞎🞎 ms, 🞎🞎🞎 Hz  Interblock interval: 🞎🞎🞎s |
|  | **NMES**  Channel: 🞎 single 🞎 dual 🞎 multi EMG trigger: 🞎 Yes 🞎 No  Brand: ____________________  Intensity: 🞎 ________ mA, 🞎 ________ mV, 🞎 other, specify_____________  Pulse Frequency: __________ Hz  Pulse width: __________ µsec  On time: __________ sec  Off time: __________ sec  Ramp up: __________ sec  Ramp down: __________sec  Duration per session (min): 🞎🞎🞎 minute  Number of sessions per day: 🞎🞎, 🞎 day/week  Total session: 🞎🞎🞎 | |

| **Intervention** | **Detail** | |
| --- | --- | --- |
| 🞎 6.TMS + FES | **TMS**  Subtype: 🞎 HF-rTMS 🞎 LF-rTMS 🞎 cTBS 🞎 iTBS  Stimulation site: 🞎 affected hemisphere 🞎 unaffected hemisphere  Brand: 🞎 Magstim 🞎 CloudTMS 🞎 MagVenture  🞎 other, specify__________________  Coil: 🞎 circular 🞎 figure of eight 🞎 other, specify __________ | |
|  | Intensity: 🞎 __________ % of motor threshold  🞎 __________ % of maximum stimulator output  🞎 other, specify_____________  Total pulses: 🞎🞎🞎🞎  Frequency: ___________ Hz  Number of trains: 🞎🞎🞎🞎  On time: __________ sec  Off time: __________ sec  Duration per session: 🞎🞎🞎 minute, 🞎🞎🞎 sec  Number of sessions per day: 🞎🞎, 🞎day/week  Total sessions: 🞎🞎🞎 | **If cTBS or iTBS add**  number of bursts: 🞎🞎🞎  intertrain interval:  🞎🞎🞎ms, 🞎🞎🞎 Hz  interblock interval: 🞎🞎🞎 s |
|  | **FES**  Channel: 🞎 single 🞎 dual 🞎 multi  Functional activity: _________________  Brand: ____________________  Intensity: 🞎 ________ mA, 🞎 ________ mV, 🞎 other, specify_____________  Pulse Frequency: __________ Hz  Pulse width: __________ µsec  On time: __________ sec  Off time: __________ sec  Ramp up: __________ sec  Ramp down: __________sec  Duration per session (min): 🞎🞎🞎 minute  Number of sessions per day: 🞎🞎, 🞎 day/week  Total session: 🞎🞎🞎 | |
| 🞎 7.Sham/ Rehab |  | |
| 🞎 8. Other |  | |

**Part V: Outcomes**

1. **Primary outcome: upper extremity function** 🞎1. Assessed 🞎2. Not assessed

If assessed, please specify

- 1. Number of scores used in the paper: 🞎
  2. Score

🞎1. FMA 🞎2. ARAT 🞎3. WMFT 🞎4. JTT
🞎5. Other, specify _____________________

- 1. Type

🞎1. Mean (SD) 🞎2. Median (IQR, range)
🞎3. Mean difference (95%CI) 🞎4. Median difference
🞎5. Mean Change from baseline 🞎6. Median change from baseline

🞎7. Other, specify __________

- 1. How many assessments after treatment 🞎, Specify: ____________________________________

1. **Secondary outcome: ADL** 🞎1. Assessed 🞎2. Not assessed

If assessed, please specify

- 1. Number of scores used in the paper: 🞎
  2. Score

🞎1. Barthel Index 🞎2. Modified Barthel Index
🞎3. Functional independence Measure (FIM) 🞎4. Other, specify _____________________

- 1. Type

🞎1. Mean (SD) 🞎2. Median (IQR, range)
🞎3. Mean difference (95%CI) 🞎4. Median difference
🞎5. Mean Change from baseline 🞎6. Median change from baseline

🞎7. Other, specify __________

- 1. How many assessments after treatment 🞎, Specify: ____________________________________

1. **Secondary outcome: Spasticity** 🞎1. Assessed 🞎2. Not assessed

If assessed, please specify

- 1. Number of scores used in the paper: 🞎
  2. Score

🞎1. MAS 🞎2. Modified Tardieu scale
🞎3.Tardieu Scale 🞎4. Other, specify _____________________

- 1. Type

🞎1. Mean (SD) 🞎2. Median (IQR, range)
🞎3. Mean difference (95%CI) 🞎4. Median difference
🞎5. Mean Change from baseline 🞎6. Median change from baseline

🞎7. Other, specify __________

- 1. How many assessments after treatment 🞎, Specify: ____________________________________

Part of body: 🞎 total 🞎 elbow 🞎 wrist 🞎 leg 🞎 ankle 🞎 other, specify______

**Part VI: Data for pooling**

**Primary outcome: Upper extremity function** Score: _____________________

| **Upper extremity function** | **Treatment group** | **n** | **Mean**  🞎Actual 🞎Change | **SD** | **Median**  🞎Actual 🞎Change | **Min/P25** | **Max/P75** | **IQR** | **Treatment comparison** | **Mean difference** | **95%CI** | |
| --- | --- | --- | --- | --- | --- | --- | --- | --- | --- | --- | --- | --- |
|  |  |  |  |  |  |  |  |  |  |  | **LL** | **UL** |
| **Time 1, after treatment**  🞎day_________  🞎week_______  🞎month______ | 🞎1.rPMS |  |  |  |  |  |  |  | 🞎1.rPMS vs 7.Sham |  |  |  |
|  | 🞎2.TMS |  |  |  |  |  |  |  | 🞎2.TMS vs 7.Sham |  |  |  |
|  | 🞎3.NMES |  |  |  |  |  |  |  | 🞎3.NMES vs 7.Sham |  |  |  |
|  | 🞎4.FES |  |  |  |  |  |  |  | 🞎4.FES vs 7.Sham |  |  |  |
|  | 🞎5.TMS+NMES |  |  |  |  |  |  |  | 🞎 |  |  |  |
|  | 🞎6.TMS+FES |  |  |  |  |  |  |  | 🞎 |  |  |  |
|  | 🞎7.Sham/Rehab |  |  |  |  |  |  |  | 🞎 |  |  |  |
|  | 🞎8.others ______ |  |  |  |  |  |  |  | 🞎 |  |  |  |
| **Time 2**  🞎day_________  🞎week_______  🞎month______ | 🞎1.rPMS |  |  |  |  |  |  |  | 🞎1.rPMS vs 7.Sham |  |  |  |
|  | 🞎2.TMS |  |  |  |  |  |  |  | 🞎2.TMS vs 7.Sham |  |  |  |
|  | 🞎3.NMES |  |  |  |  |  |  |  | 🞎3.NMES vs 7.Sham |  |  |  |
|  | 🞎4.FES |  |  |  |  |  |  |  | 🞎4.FES vs 7.Sham |  |  |  |
|  | 🞎5.TMS+NMES |  |  |  |  |  |  |  | 🞎 |  |  |  |
|  | 🞎6.TMS+FES |  |  |  |  |  |  |  | 🞎 |  |  |  |
|  | 🞎7.Sham/Rehab |  |  |  |  |  |  |  | 🞎 |  |  |  |
|  | 🞎8.others ______ |  |  |  |  |  |  |  | 🞎 |  |  |  |
| **Time 3**  🞎day_________  🞎week_______  🞎month______ | 🞎1.rPMS |  |  |  |  |  |  |  | 🞎1.rPMS vs 7.Sham |  |  |  |
|  | 🞎2.TMS |  |  |  |  |  |  |  | 🞎2.TMS vs 7.Sham |  |  |  |
|  | 🞎3.NMES |  |  |  |  |  |  |  | 🞎3.NMES vs 7.Sham |  |  |  |
|  | 🞎4.FES |  |  |  |  |  |  |  | 🞎4.FES vs 7.Sham |  |  |  |
|  | 🞎5.TMS+NMES |  |  |  |  |  |  |  | 🞎 |  |  |  |
|  | 🞎6.TMS+FES |  |  |  |  |  |  |  | 🞎 |  |  |  |
|  | 🞎7.Sham/Rehab |  |  |  |  |  |  |  | 🞎 |  |  |  |
|  | 🞎8.others ______ |  |  |  |  |  |  |  | 🞎 |  |  |  |
| **Time 4**  🞎day_________  🞎week_______  🞎month______ | 🞎1.rPMS |  |  |  |  |  |  |  | 🞎1.rPMS vs 7.Sham |  |  |  |
|  | 🞎2.TMS |  |  |  |  |  |  |  | 🞎2.TMS vs 7.Sham |  |  |  |
|  | 🞎3.NMES |  |  |  |  |  |  |  | 🞎3.NMES vs 7.Sham |  |  |  |
|  | 🞎4.FES |  |  |  |  |  |  |  | 🞎4.FES vs 7.Sham |  |  |  |
|  | 🞎5.TMS+NMES |  |  |  |  |  |  |  | 🞎 |  |  |  |
|  | 🞎6.TMS+FES |  |  |  |  |  |  |  | 🞎 |  |  |  |
|  | 🞎7.Sham/Rehab |  |  |  |  |  |  |  | 🞎 |  |  |  |
|  | 🞎8.others ______ |  |  |  |  |  |  |  | 🞎 |  |  |  |
| **Time 5**  🞎day_________  🞎week_______  🞎month______ | 🞎1.rPMS |  |  |  |  |  |  |  | 🞎1.rPMS vs 7.Sham |  |  |  |
|  | 🞎2.TMS |  |  |  |  |  |  |  | 🞎2.TMS vs 7.Sham |  |  |  |
|  | 🞎3.NMES |  |  |  |  |  |  |  | 🞎3.NMES vs 7.Sham |  |  |  |
|  | 🞎4.FES |  |  |  |  |  |  |  | 🞎4.FES vs 7.Sham |  |  |  |
|  | 🞎5.TMS+NMES |  |  |  |  |  |  |  | 🞎 |  |  |  |
|  | 🞎6.TMS+FES |  |  |  |  |  |  |  | 🞎 |  |  |  |
|  | 🞎7.Sham/Rehab |  |  |  |  |  |  |  | 🞎 |  |  |  |
|  | 🞎8.others ______ |  |  |  |  |  |  |  | 🞎 |  |  |  |

**Secondary outcome: ADL** Score: _____________________

| **ADL** | **Treatment group** | **n** | **Mean**  🞎Actual 🞎Change | **SD** | **Median**  🞎Actual 🞎Change | **Min/P25** | **Max/P75** | **IQR** | **Treatment comparison** | **Mean difference** | **95%CI** | |
| --- | --- | --- | --- | --- | --- | --- | --- | --- | --- | --- | --- | --- |
|  |  |  |  |  |  |  |  |  |  |  | **LL** | **UL** |
| **Time 1, after treatment**  🞎day_________  🞎week_______  🞎month______ | 🞎1.rPMS |  |  |  |  |  |  |  | 🞎1.rPMS vs 7.Sham |  |  |  |
|  | 🞎2.TMS |  |  |  |  |  |  |  | 🞎2.TMS vs 7.Sham |  |  |  |
|  | 🞎3.NMES |  |  |  |  |  |  |  | 🞎3.NMES vs 7.Sham |  |  |  |
|  | 🞎4.FES |  |  |  |  |  |  |  | 🞎4.FES vs 7.Sham |  |  |  |
|  | 🞎5.TMS+NMES |  |  |  |  |  |  |  | 🞎 |  |  |  |
|  | 🞎6.TMS+FES |  |  |  |  |  |  |  | 🞎 |  |  |  |
|  | 🞎7.Sham/Rehab |  |  |  |  |  |  |  | 🞎 |  |  |  |
|  | 🞎8.others ______ |  |  |  |  |  |  |  | 🞎 |  |  |  |
| **Time 2**  🞎day_________  🞎week_______  🞎month______ | 🞎1.rPMS |  |  |  |  |  |  |  | 🞎1.rPMS vs 7.Sham |  |  |  |
|  | 🞎2.TMS |  |  |  |  |  |  |  | 🞎2.TMS vs 7.Sham |  |  |  |
|  | 🞎3.NMES |  |  |  |  |  |  |  | 🞎3.NMES vs 7.Sham |  |  |  |
|  | 🞎4.FES |  |  |  |  |  |  |  | 🞎4.FES vs 7.Sham |  |  |  |
|  | 🞎5.TMS+NMES |  |  |  |  |  |  |  | 🞎 |  |  |  |
|  | 🞎6.TMS+FES |  |  |  |  |  |  |  | 🞎 |  |  |  |
|  | 🞎7.Sham/Rehab |  |  |  |  |  |  |  | 🞎 |  |  |  |
|  | 🞎8.others ______ |  |  |  |  |  |  |  | 🞎 |  |  |  |
| **Time 3**  🞎day_________  🞎week_______  🞎month______ | 🞎1.rPMS |  |  |  |  |  |  |  | 🞎1.rPMS vs 7.Sham |  |  |  |
|  | 🞎2.TMS |  |  |  |  |  |  |  | 🞎2.TMS vs 7.Sham |  |  |  |
|  | 🞎3.NMES |  |  |  |  |  |  |  | 🞎3.NMES vs 7.Sham |  |  |  |
|  | 🞎4.FES |  |  |  |  |  |  |  | 🞎4.FES vs 7.Sham |  |  |  |
|  | 🞎5.TMS+NMES |  |  |  |  |  |  |  | 🞎 |  |  |  |
|  | 🞎6.TMS+FES |  |  |  |  |  |  |  | 🞎 |  |  |  |
|  | 🞎7.Sham/Rehab |  |  |  |  |  |  |  | 🞎 |  |  |  |
|  | 🞎8.others ______ |  |  |  |  |  |  |  | 🞎 |  |  |  |
| **Time 4**  🞎day_________  🞎week_______  🞎month______ | 🞎1.rPMS |  |  |  |  |  |  |  | 🞎1.rPMS vs 7.Sham |  |  |  |
|  | 🞎2.TMS |  |  |  |  |  |  |  | 🞎2.TMS vs 7.Sham |  |  |  |
|  | 🞎3.NMES |  |  |  |  |  |  |  | 🞎3.NMES vs 7.Sham |  |  |  |
|  | 🞎4.FES |  |  |  |  |  |  |  | 🞎4.FES vs 7.Sham |  |  |  |
|  | 🞎5.TMS+NMES |  |  |  |  |  |  |  | 🞎 |  |  |  |
|  | 🞎6.TMS+FES |  |  |  |  |  |  |  | 🞎 |  |  |  |
|  | 🞎7.Sham/Rehab |  |  |  |  |  |  |  | 🞎 |  |  |  |
|  | 🞎8.others ______ |  |  |  |  |  |  |  | 🞎 |  |  |  |
| **Time 5**  🞎day_________  🞎week_______  🞎month______ | 🞎1.rPMS |  |  |  |  |  |  |  | 🞎1.rPMS vs 7.Sham |  |  |  |
|  | 🞎2.TMS |  |  |  |  |  |  |  | 🞎2.TMS vs 7.Sham |  |  |  |
|  | 🞎3.NMES |  |  |  |  |  |  |  | 🞎3.NMES vs 7.Sham |  |  |  |
|  | 🞎4.FES |  |  |  |  |  |  |  | 🞎4.FES vs 7.Sham |  |  |  |
|  | 🞎5.TMS+NMES |  |  |  |  |  |  |  | 🞎 |  |  |  |
|  | 🞎6.TMS+FES |  |  |  |  |  |  |  | 🞎 |  |  |  |
|  | 🞎7.Sham/Rehab |  |  |  |  |  |  |  | 🞎 |  |  |  |
|  | 🞎8.others ______ |  |  |  |  |  |  |  | 🞎 |  |  |  |

**Secondary outcome: Spasticity**  Score: _____________________

Part of body: 🞎 total 🞎 elbow 🞎 wrist
 🞎 leg 🞎 ankle 🞎 other, specify _______

| **Spasticity** | **Treatment group** | **n** | **Mean**  🞎Actual 🞎Change | **SD** | **Median**  🞎Actual 🞎Change | **Min/P25** | **Max/P75** | **IQR** | **Treatment comparison** | **Mean difference** | **95%CI** | |
| --- | --- | --- | --- | --- | --- | --- | --- | --- | --- | --- | --- | --- |
|  |  |  |  |  |  |  |  |  |  |  | **LL** | **UL** |
| **Time 1, after treatment**  🞎minute _____  🞎day_________  🞎week_______  🞎month______ | 🞎1.rPMS |  |  |  |  |  |  |  | 🞎1.rPMS vs 7.Sham |  |  |  |
|  | 🞎2.TMS |  |  |  |  |  |  |  | 🞎2.TMS vs 7.Sham |  |  |  |
|  | 🞎3.NMES |  |  |  |  |  |  |  | 🞎3.NMES vs 7.Sham |  |  |  |
|  | 🞎4.FES |  |  |  |  |  |  |  | 🞎4.FES vs 7.Sham |  |  |  |
|  | 🞎5.TMS+NMES |  |  |  |  |  |  |  | 🞎 |  |  |  |
|  | 🞎6.TMS+FES |  |  |  |  |  |  |  | 🞎 |  |  |  |
|  | 🞎7.Sham/Rehab |  |  |  |  |  |  |  | 🞎 |  |  |  |
|  | 🞎8.others ______ |  |  |  |  |  |  |  | 🞎 |  |  |  |
| **Time 2**  🞎minute _____  🞎day_________  🞎week_______  🞎month______ | 🞎1.rPMS |  |  |  |  |  |  |  | 🞎1.rPMS vs 7.Sham |  |  |  |
|  | 🞎2.TMS |  |  |  |  |  |  |  | 🞎2.TMS vs 7.Sham |  |  |  |
|  | 🞎3.NMES |  |  |  |  |  |  |  | 🞎3.NMES vs 7.Sham |  |  |  |
|  | 🞎4.FES |  |  |  |  |  |  |  | 🞎4.FES vs 7.Sham |  |  |  |
|  | 🞎5.TMS+NMES |  |  |  |  |  |  |  | 🞎 |  |  |  |
|  | 🞎6.TMS+FES |  |  |  |  |  |  |  | 🞎 |  |  |  |
|  | 🞎7.Sham/Rehab |  |  |  |  |  |  |  | 🞎 |  |  |  |
|  | 🞎8.others ______ |  |  |  |  |  |  |  | 🞎 |  |  |  |
| **Time 3**  🞎minute _____  🞎day_________  🞎week_______  🞎month______ | 🞎1.rPMS |  |  |  |  |  |  |  | 🞎1.rPMS vs 7.Sham |  |  |  |
|  | 🞎2.TMS |  |  |  |  |  |  |  | 🞎2.TMS vs 7.Sham |  |  |  |
|  | 🞎3.NMES |  |  |  |  |  |  |  | 🞎3.NMES vs 7.Sham |  |  |  |
|  | 🞎4.FES |  |  |  |  |  |  |  | 🞎4.FES vs 7.Sham |  |  |  |
|  | 🞎5.TMS+NMES |  |  |  |  |  |  |  | 🞎 |  |  |  |
|  | 🞎6.TMS+FES |  |  |  |  |  |  |  | 🞎 |  |  |  |
|  | 🞎7.Sham/Rehab |  |  |  |  |  |  |  | 🞎 |  |  |  |
|  | 🞎8.others ______ |  |  |  |  |  |  |  | 🞎 |  |  |  |
| **Time 4**  🞎minute _____  🞎day_________  🞎week_______  🞎month______ | 🞎1.rPMS |  |  |  |  |  |  |  | 🞎1.rPMS vs 7.Sham |  |  |  |
|  | 🞎2.TMS |  |  |  |  |  |  |  | 🞎2.TMS vs 7.Sham |  |  |  |
|  | 🞎3.NMES |  |  |  |  |  |  |  | 🞎3.NMES vs 7.Sham |  |  |  |
|  | 🞎4.FES |  |  |  |  |  |  |  | 🞎4.FES vs 7.Sham |  |  |  |
|  | 🞎5.TMS+NMES |  |  |  |  |  |  |  | 🞎 |  |  |  |
|  | 🞎6.TMS+FES |  |  |  |  |  |  |  | 🞎 |  |  |  |
|  | 🞎7.Sham/Rehab |  |  |  |  |  |  |  | 🞎 |  |  |  |
|  | 🞎8.others ______ |  |  |  |  |  |  |  | 🞎 |  |  |  |
| **Time 5**  🞎minute _____  🞎day_________  🞎week_______  🞎month______ | 🞎1.rPMS |  |  |  |  |  |  |  | 🞎1.rPMS vs 7.Sham |  |  |  |
|  | 🞎2.TMS |  |  |  |  |  |  |  | 🞎2.TMS vs 7.Sham |  |  |  |
|  | 🞎3.NMES |  |  |  |  |  |  |  | 🞎3.NMES vs 7.Sham |  |  |  |
|  | 🞎4.FES |  |  |  |  |  |  |  | 🞎4.FES vs 7.Sham |  |  |  |
|  | 🞎5.TMS+NMES |  |  |  |  |  |  |  | 🞎 |  |  |  |
|  | 🞎6.TMS+FES |  |  |  |  |  |  |  | 🞎 |  |  |  |
|  | 🞎7.Sham/Rehab |  |  |  |  |  |  |  | 🞎 |  |  |  |
|  | 🞎8.others ______ |  |  |  |  |  |  |  | 🞎 |  |  |  |

**Part VI. II: Data for pooling (categorical variable)** Outcome 🞎 upper extremity function 🞎 ADL🞎 Spasticity

If spasticity, please specify part of body:
🞎 total 🞎 elbow 🞎 wrist 🞎 leg 🞎 ankle

🞎 other, specify _________

Score: ________________________________

|  | **Treatment** | **Outcome group** | **n** | **n of each arm** | **Treatment comparison** | **RR** | **95%CI** | |
| --- | --- | --- | --- | --- | --- | --- | --- | --- |
|  |  |  |  |  |  |  | **LL** | **UL** |
| **Time 1**  🞎minute _____  🞎day_________  🞎week_______  🞎month______ | 🞎1.rPMS  🞎2.TMS  🞎3.NMES  🞎4.FES  🞎5.TMS+NMES  🞎6.TMS+FES  🞎7.Sham/Rehab  🞎8.others ______ |  |  |  | 🞎1.rPMS vs 7.Sham  🞎2.TMS vs 7.Sham  🞎3.NMES vs 7.Sham  🞎4.FES vs 7.Sham  🞎___________________ |  |  |  |
|  |  |  |  |  |  |  |  |  |
|  |  |  |  |  |  |  |  |  |
|  |  |  |  |  |  |  |  |  |
|  |  |  |  |  |  |  |  |  |
|  | 🞎1.rPMS  🞎2.TMS  🞎3.NMES  🞎4.FES  🞎5.TMS+NMES  🞎6.TMS+FES  🞎7.Sham/Rehab  🞎8.others ______ |  |  |  | 🞎1.rPMS vs 7.Sham  🞎2.TMS vs 7.Sham  🞎3.NMES vs 7.Sham  🞎4.FES vs 7.Sham  🞎___________________ |  |  |  |
|  |  |  |  |  |  |  |  |  |
|  |  |  |  |  |  |  |  |  |
|  |  |  |  |  |  |  |  |  |
|  |  |  |  |  |  |  |  |  |
| **Time 2**  🞎minute _____  🞎day_________  🞎week_______  🞎month______ | 🞎1.rPMS  🞎2.TMS  🞎3.NMES  🞎4.FES  🞎5.TMS+NMES  🞎6.TMS+FES  🞎7.Sham/Rehab  🞎8.others ______ |  |  |  | 🞎1.rPMS vs 7.Sham  🞎2.TMS vs 7.Sham  🞎3.NMES vs 7.Sham  🞎4.FES vs 7.Sham  🞎___________________ |  |  |  |
|  |  |  |  |  |  |  |  |  |
|  |  |  |  |  |  |  |  |  |
|  |  |  |  |  |  |  |  |  |
|  |  |  |  |  |  |  |  |  |
|  | 🞎1.rPMS  🞎2.TMS  🞎3.NMES  🞎4.FES  🞎5.TMS+NMES  🞎6.TMS+FES  🞎7.Sham/Rehab  🞎8.others ______ |  |  |  | 🞎1.rPMS vs 7.Sham  🞎2.TMS vs 7.Sham  🞎3.NMES vs 7.Sham  🞎4.FES vs 7.Sham  🞎___________________ |  |  |  |
|  |  |  |  |  |  |  |  |  |
|  |  |  |  |  |  |  |  |  |
|  |  |  |  |  |  |  |  |  |
|  |  |  |  |  |  |  |  |  |
| **Time 3**  🞎minute _____  🞎day_________  🞎week_______  🞎month______ | 🞎1.rPMS  🞎2.TMS  🞎3.NMES  🞎4.FES  🞎5.TMS+NMES  🞎6.TMS+FES  🞎7.Sham/Rehab  🞎8.others ______ |  |  |  | 🞎1.rPMS vs 7.Sham  🞎2.TMS vs 7.Sham  🞎3.NMES vs 7.Sham  🞎4.FES vs 7.Sham  🞎___________________ |  |  |  |
|  |  |  |  |  |  |  |  |  |
|  |  |  |  |  |  |  |  |  |
|  |  |  |  |  |  |  |  |  |
|  |  |  |  |  |  |  |  |  |
|  | 🞎1.rPMS  🞎2.TMS  🞎3.NMES  🞎4.FES  🞎5.TMS+NMES  🞎6.TMS+FES  🞎7.Sham/Rehab  🞎8.others ______ |  |  |  | 🞎1.rPMS vs 7.Sham  🞎2.TMS vs 7.Sham  🞎3.NMES vs 7.Sham  🞎4.FES vs 7.Sham  🞎___________________ |  |  |  |
|  |  |  |  |  |  |  |  |  |
|  |  |  |  |  |  |  |  |  |
|  |  |  |  |  |  |  |  |  |
|  |  |  |  |  |  |  |  |  |
| **Time 4**  🞎minute _____  🞎day_________  🞎week_______  🞎month______ | 🞎1.rPMS  🞎2.TMS  🞎3.NMES  🞎4.FES  🞎5.TMS+NMES  🞎6.TMS+FES  🞎7.Sham/Rehab  🞎8.others _____ |  |  |  | 🞎1.rPMS vs 7.Sham  🞎2.TMS vs 7.Sham  🞎3.NMES vs 7.Sham  🞎4.FES vs 7.Sham  🞎___________________ |  |  |  |
|  |  |  |  |  |  |  |  |  |
|  |  |  |  |  |  |  |  |  |
|  |  |  |  |  |  |  |  |  |
|  |  |  |  |  |  |  |  |  |
|  | 🞎1.rPMS  🞎2.TMS  🞎3.NMES  🞎4.FES  🞎5.TMS+NMES  🞎6.TMS+FES  🞎7.Sham/Rehab  🞎8.others ______ |  |  |  | 🞎1.rPMS vs 7.Sham  🞎2.TMS vs 7.Sham  🞎3.NMES vs 7.Sham  🞎4.FES vs 7.Sham  🞎___________________ |  |  |  |
|  |  |  |  |  |  |  |  |  |
|  |  |  |  |  |  |  |  |  |
|  |  |  |  |  |  |  |  |  |
|  |  |  |  |  |  |  |  |  |

**Appendix 3:** Risk of bias assessment form (Revised Cochrane risk-of-bias tool for randomized trials (ROB2))

Study ID: 🞎🞎🞎🞎 Reviewer: 🞎1. AK 🞎2. MS

Responses underlined in green are potential markers for low risk of bias, and responses in red are potential markers for a risk of bias. Where questions relate only to sign posts to other questions, no formatting is used.

| **Domain** | **Question** | **Results** |
| --- | --- | --- |
| 1.Randomization process | 1.1 Was the allocation sequence random? | 🞎 Y/PY  🞎 PN/N  🞎 NI |
|  | 1.2 Was the allocation sequence concealed until participants were enrolled and assigned to interventions? | 🞎 Y/PY  🞎 PN/N  🞎 NI |
|  | 1.3 Did baseline differences between intervention groups suggest a problem with the randomization process? | 🞎 Y/PY  🞎 PN/N  🞎 NI |
|  | **Risk-of-bias judgement** | 🞎 Low  🞎 High  🞎 Some concerns |
| 2.Intended interventions  (*Effect of assignment to intervention*) | 2.1. Were participants aware of their assigned intervention during the trial? | 🞎 Y/PY  🞎 PN/N  🞎 NI |
|  | 2.2. Were carers and people delivering the interventions aware of participants' assigned intervention during the trial? | 🞎 Y/PY  🞎 PN/N  🞎 NI |
|  | 2.3. If Y/PY/NI to 2.1 or 2.2: Were there deviations from the intended intervention that arose because of the trial context? | 🞎 NA  🞎 Y/PY  🞎 PN/N  🞎 NI |
|  | 2.4 If Y/PY to 2.3: Were these deviations likely to have affected the outcome? | 🞎 NA  🞎 Y/PY  🞎 PN/N  🞎 NI |
|  | 2.5. If Y/PY/NI to 2.4: Were these deviations from intended intervention balanced between groups? | 🞎 NA  🞎 Y/PY  🞎 PN/N  🞎 NI |
|  | 2.6 Was an appropriate analysis used to estimate the effect of assignment to intervention? | 🞎 Y/PY  🞎 PN/N  🞎 NI |
|  | 2.7 If N/PN/NI to 2.6: Was there potential for a substantial impact (on the result) of the failure to analyse participants in the group to which they were randomized? | 🞎 NA  🞎 Y/PY  🞎 PN/N  🞎 NI |
|  | **Risk-of-bias judgement** | 🞎 Low  🞎 High  🞎 Some concerns |

| **Domain** | **Question** | **Results** |
| --- | --- | --- |
| 2.Intended interventions  (*Effect of adhering to intervention*) | 2.1. Were participants aware of their assigned intervention during the trial? | 🞎 Y/PY  🞎 PN/N  🞎 NI |
|  | 2.2. Were carers and people delivering the interventions aware of participants' assigned intervention during the trial? | 🞎 Y/PY  🞎 PN/N  🞎 NI |
|  | 2.3. [If applicable:] If Y/PY/NI to 2.1 or 2.2: Were important non-protocol interventions balanced across intervention groups? | 🞎 NA  🞎 Y/PY  🞎 PN/N  🞎 NI |
|  | 2.4. [If applicable:] Were there failures in implementing the intervention that could have affected the outcome? | 🞎 NA  🞎 Y/PY  🞎 PN/N  🞎 NI |
|  | 2.5. [If applicable:] Was there non-adherence to the assigned intervention regimen that could have affected participants’ outcomes? | 🞎 NA  🞎 Y/PY  🞎 PN/N  🞎 NI |
|  | 2.6. If N/PN/NI to 2.3, or Y/PY/NI to 2.4 or 2.5: Was an appropriate analysis used to estimate the effect of adhering to the intervention? | 🞎 NA  🞎 Y/PY  🞎 PN/N  🞎 NI |
|  | **Risk-of-bias judgement** | 🞎 Low  🞎 High  🞎 Some concerns |
| 3.Missing outcome data | 3.1 Were data for this outcome available for all, or nearly all, participants randomized? | 🞎 Y/PY  🞎 PN/N  🞎 NI |
|  | 3.2 If N/PN/NI to 3.1: Is there evidence that the result was not biased by missing outcome data? | 🞎 NA  🞎 Y/PY  🞎 PN/N  🞎 NI |
|  | 3.3 If N/PN to 3.2: Could missingness in the outcome depend on its true value? | 🞎 NA  🞎 Y/PY  🞎 PN/N  🞎 NI |
|  | 3.4 If Y/PY/NI to 3.3: Is it likely that missingness in the outcome depended on its true value? | 🞎 NA  🞎 Y/PY  🞎 PN/N  🞎 NI |
|  | **Risk-of-bias judgement** | 🞎 Low  🞎 High  🞎 Some concerns |

| **Domain** | **Question** | **Results** |
| --- | --- | --- |
| 4.Measurement of the outcome | 4.1 Was the method of measuring the outcome inappropriate? | 🞎 Y/PY  🞎 PN/N  🞎 NI |
|  | 4.2 Could measurement or ascertainment of the outcome have differed between intervention groups? | 🞎 Y/PY  🞎 PN/N  🞎 NI |
|  | 4.3 If N/PN/NI to 4.1 and 4.2: Were outcome assessors aware of the intervention received by study participants? | 🞎 NA  🞎 Y/PY  🞎 PN/N  🞎 NI |
|  | 4.4 If Y/PY/NI to 4.3: Could assessment of the outcome have been influenced by knowledge of intervention received? | 🞎 NA  🞎 Y/PY  🞎 PN/N  🞎 NI |
|  | 4.5 If Y/PY/NI to 4.4: Is it likely that assessment of the outcome was influenced by knowledge of intervention received? | 🞎 NA  🞎 Y/PY  🞎 PN/N  🞎 NI |
|  | **Risk-of-bias judgement** | 🞎 Low  🞎 High  🞎 Some concerns |
| 5.Selection of the reported results | 5.1 Were the data that produced this result analysed in accordance with a pre-specified analysis plan that was finalized before unblinded outcome data were available for analysis? | 🞎 Y/PY  🞎 PN/N  🞎 NI |
|  | Is the numerical result being assessed likely to have been selected, on the basis of the results, from... | |
|  | 5.2. ... multiple eligible outcome measurements (e.g., scales, definitions, time points) within the outcome domain? | 🞎 Y/PY  🞎 PN/N  🞎 NI |
|  | 5.3 ... multiple eligible analyses of the data? | 🞎 Y/PY  🞎 PN/N  🞎 NI |
|  | **Risk-of-bias judgement** | 🞎 Low  🞎 High  🞎 Some concerns |
| **Overall risk of bias** | 🞎 Low  🞎 High  🞎 Some concerns | |

| **Overall risk-of-bias judgement** | **Criteria** |
| --- | --- |
| **Low risk of bias** | The study is judged to be at low risk of bias for **all domains** for this result. |
| **Some concerns** | The study is judged to raise some concerns in **at least one domain** for this result, but not to be at high risk of bias for any domain |
| **High risk of bias** | The study is judged to be **at high risk of bias in at least one domain** for this result. Or The study is judged to have some concerns for multiple domains in a way that substantially lowers confidence in the result. |

**Appendix 4:** Interventions used in each included study

**A)** NMES

| **Author** | **EMG-trigger** | **Brand** | **Site** | **Intensity** | **Frequency (Hz)** | **Pulse wide (µs)** | **On/off time (sec)** | **Ramp up / ramp down (sec)** | **Duration** | **No. of session** |
| --- | --- | --- | --- | --- | --- | --- | --- | --- | --- | --- |
| Jiang. YF^10^ | no | Vital Stim 5951 | supraspinatus, deltoid, triceps | 20-30 mA | no information | no information | no information | no information | 20 min | 14 |
| Kim. HN^90^ | no | Novastim | EPB muscle | 30-70 mA | 35 | 250 | 5/5 | no information | 30 min | 20 |
| Fletcher-Smith. JC^55^ | no | no information | flexor and extensor | no information | 40-60 | 450 | no information | no information | 30 min | 120 |
| Dorsch. S^91^ | yes | no information | 4 muscles group of UE | 10-80 mA | 70 | 100-250 | 10/10 | 1/1 | no information | 20 |
| Sahin. N^92^ | no | no information | wrist extensor | no information | 100 | 100 | 3/9 | no information | no information | 20 |
| Rosewillium. S^59^ | no | Conformite Europenne-marked | wrist and finger extensor | no information | 40 | 300 | 15/15 | 6/6 | 30 min | 60 |
| Lin. Z^40^ | no | Respond select II | supraspinatus, deltoid, wrist extensor | maximum tolerance | 30 | 300 | 5/5 | 1/1 | 30 min | 15 |
| Church. C^93^ | no | no information | supraspinatus, posterior deltoid | gross muscle contraction | 30 | no information | 15/15 | 3/3 | 60 min | 12 |
| Mann. GE^94^ | no | no information | Upper limb | full elbow, wrist, and finger extension | 40 | 300 | 8/8 | 2/2 | 10-30 min | 168 |
| Kimberley. TJ^95^ | no | Danmeter | finger and wrist extensor | finger and wrist extension | 50 | 200 | 5/15 | 1/1 | 6 hours | 10 |
| Powell. J^58^ | no | no information | wrist and finger extensor | full joint extension | 20 | 300 | no information | 1/1.5 | 30 min | 168 |
| Francisco. G^27^ | yes | Automove Stimulator | ECR muscle | no information | 20-100 | no information | 5/5 | no information | 30 min | no information |
| Chae. J^23^ | no | FOCUS | EDC, ECR muscles | 0-60 mA, full wrist, and finger extension | 25-50 | 300 | 10/10 | 2/2 | 60 min | 15 |
| Shin. HK^100^ | yes^27^ | Walking man II | EDC muscle | 10-20 mA | 35 | 200 | 5/4 | 0.1/2 | 30 min | 50 |
| Schick. T^46^ | no | STIWELL | ECR muscle | visible grip motion | 30 | 300 | 6/14 | no information | 30 min | 15 |
| Tosun. A^50^ | no | BTL TENS 15 | wrist extensor and EDC | extension finger and wrist | 50 | 180 | no information | no information | no information | 20 |
| Du. J^26^ | no | RH-ZP-D | ECR, EDC muscles | significant muscle contraction | 50 | 200 | no information | no information | 30 min | 20 |
| Tarri. M^48^ | no | Digitimer | ECR | no information |  |  |  |  | 30 min | 5 |
| Tilkici. M^49^ | no | Optimed Ultra | wrist and finger extensor | no information | 20-50 | 200-500 | 2/2 | no information | 30 min | 15 |

Abbreviations: ECR, extensor carpi radialis; EDC, extensor digitorum communis; EPB, extensor pollicis brevis; UE, upper extremity.

| **Author** | **EMG-trigger** | **Brand** | **Site** | **Activity** | **Intensity** | **Frequency** | **Pulse wide** | **On/off time (sec)** | **Ramp up / ramp down (sec)** | **Duration** | **No. of session** |
| --- | --- | --- | --- | --- | --- | --- | --- | --- | --- | --- | --- |
| Haghighi. FM^31^ | no | no information | no information | no information | no information | 25 Hz | 0-250 µs | no information | no information | 20 min | 10 |
| Niu. CM^44^ | no | no information | bicep, triceps, deltoid, pectoralis major, BCR | reaching movement | no information | 50 Hz | 200 µs | no information | no information | 60-90 min | 5 |
| Kirac-Unal. Z^96^ | yes | no information | ECR, triceps | reach and grasp | 15-30 mA | 35 Hz | 200 µs | 10/15 | no information | 15 min | 20 |
| Karaahmet. OZ^36^ | no | RehaStim | posterior deltoid, bicep, triceps | cycling | visible muscle contraction | 20 Hz | 300 µs | no information | no information | 15 min | 20 |
| Nakipoglu-Yuzer. GF^57^ | no | no information | no information | no information | no information | 30 Hz | 200 µs | 10/10 | 2/2 | 30 min | 20 |
| Jonsdottir. J^35^ | no | MeCFES | no information | reaching | no information | no information | no information | no information | no information | 45 min | 25 |
| Thorsen. R^97^ | no | MeCFES | no information | reaching, grasping | 10 - 20 mA | no information | no information | no information | no information | 20 min | 25 |
| Karakus. D^98^ | no | Samms Professional | wrist and finger extensors | no information | full wrist and finger extension | 36 Hz | 250 µs | 10/12 | 3/3 | 30 min | 10 |
| Mohamed-Faisal. CK^99^ | no | no information | flexor and extensor arm | grasping, releasing | flexor and extensor arm | no information | no information | no information | no information | 20 min | 24 |
| Mangold. S^64^ | no | no information | no information | reach, grasp and release |  | 25 Hz | 0-250 µs |  |  | 45 min | 12 |
| Chan. MK^24^ | no | ADXL202 | EDS, APL | moving a bowl, pushing a basketball, simulate feeding, simulate drinking | to open hand | 40 Hz | 200 µs | 3/- | 3/2 | 20 min | 15 |
| Jian. L^41^ | no | OG GIKEN KR-7 | deltoid, wrist extensor, upper arm extensor muscles | no information | limit to patient tolerance | 1-100 Hz | no information | 1-30/0-60 | 0-10/0-10 | 30 min | 72 |
| Schick. T^46^ | yes | STIWELL | ECR, FDS,Infraspinatus, tricep | reaching, gripping, lateral lifting | visible grip motion | 30 Hz | 300 µs | no information | 1.5/1.5 | 30 min | 15 |
| Khan. F^37^ | no | MegaXP |  | grasp, flexion/extension, pattern movement | 10-50 mA | 35 Hz | 200 µs | no information | no information | 30 min | 12 |

**B)** FES

Abbreviations: APL, abductor pollicis longus; BCR, brachioradialis; ECR, extensor carpi radialis; EDS, extensor digitorum superficialis; FDS, flexor digitorum superficialis.

| **Author** | **Subtype** | **Site** | **Brand** | **Coil** | **Intensity** | **Pulse** | **Frequency (Hz)** | **On/off time (sec)** | **Duration** | **No. of session** |
| --- | --- | --- | --- | --- | --- | --- | --- | --- | --- | --- |
| Luk. KY^42^ | lfTMS | unaffected hemisphere | MagStim | figure-of-eight | 90% motor threshold | 1,200 | 1 | no information | no information | 10 |
| Haghighi. FM^31^ | hfTMS | affected hemisphere | MagStim | figure-of-eight | 90% motor threshold | 2,000 | 20 | 5/50 | no information | 10 |
| Gottlieb. A^29^ | lfTMS | unaffected hemisphere | PowerMag | figure-of-eight | 100% motor threshold | 1,200 | 1 | no information | no information | 10 |
| Sharma. H^54^ | lfTMS | unaffected hemisphere | MagStim | figure-of-eight | 110% motor threshold | 750 | 1 | -/45 | no information | 14 |
| Kim. WS^38^ | lfTMS | unaffected hemisphere | ALTMS | figure-of-eight | 100% motor threshold | 1,800 | 1 | no information | 30 min | 10 |
| Chen. JY^25^ | iTBS | affected hemisphere | MagStim | figure-of-eight | 80% active motor threshold | 600 | 50 | no information | no information | 10 |
| Tretriluxana. J^85^ | lfTMS | unaffected hemisphere | MagStim | figure-of-eight | 90% motor threshold | 1,200 | 1 | no information | no information | 1 |
| Harvey. RL^32^ | lfTMS | unaffected hemisphere | Nexstim | figure-of-eight | 110% motor threshold | 900 | 1 | no information | no information | 18 |
| Wang. HB^52^ | lfTMS | unaffected hemisphere | YRD CCY-1 | figure-of-eight | 90% motor threshold | 1,200 | 1 | no information | 20 min | 24 |
| Ozjeskin. M^61^ | lfTMS | unaffected hemisphere | Nexstim | figure-of-eight | 90% motor threshold | 1,500 | 1 | no information | 25 min | 10 |
| Meng. ZY^56^ | lfTMS | unaffected hemisphere | MagStim | figure-of-eight | 90% motor threshold | 1,800 | 1 | no information | 30 min | 14 |
| Guan. YZ^30^ | hfTMS | affected hemisphere | MagPro | figure-of-eight | 120% motor threshold | 1,000 | 5 | -/2 | no information | no information |
| Askin. A^22^ | lfTMS | unaffected hemisphere | Remed | figure-of-eight | 90% motor threshold | 1,200 | 1 | no information | 20 min | 10 |
| Hosomi. K^33^ | hfTMS | affected hemisphere | MagStim | figure-of-eight | 90% motor threshold | 500 | 5 | 10/50 | no information | 10 |
| Ackerley. SJ^86^ | iTBS | affected hemisphere | MagStim | no information | 90% motor threshold | 600 | no information | no information | no information | 10 |
| Matsuura. A^43^ | lfTMS | unaffected hemisphere | MagStim | figure-of-eight | 100% motor threshold, 43.6%max output | 1,200 | 1 | no information | 20 min | 5 |
| Vaziri. PM^51^ | lfTMS | unaffected hemisphere | no information | figure-of-eight | 60-80% motor threshold | no information | 1 | no information | 20 min | 10 |
| Rose. DK^45^ | lfTMS | unaffected hemisphere | MagStim | figure-of-eight | 100% motor threshold | 1,200 | 1 | no information | no information | 16 |
| Galvao. SC^28^ | lfTMS | unaffected hemisphere | no information | figure-of-eight | 90% motor threshold  Abbreviations: cTBS, continuous theta burst stimulation; hfTMS, high frequency transcranial magnetic stimulation; iTBS, intermittent theta burst stimulation; lfTMS, low frequency transcranial magnetic stimulation. | 1,500 | 1 | no information | no information | 10 |

**C)** TMS

**C)** TMS (cont.)

| **Author** | **Subtype** | **Site** | **Brand** | **Coil** | **Intensity** | **Pulse** | **Frequency (Hz)** | **On/off time (sec)** | **Duration** | **No. of session** |
| --- | --- | --- | --- | --- | --- | --- | --- | --- | --- | --- |
| Hsu. YF^34^ | iTBS | affected hemisphere | MagStim | figure-of-eight | 80% motor threshold | 1,200 | no information | no information | 10 min | 10 |
| Higgins. J^87^ | lfTMS | unaffected hemisphere | MagStim | figure-of-eight | 110% motor threshold | 1,200 | 1 | no information | no information | 8 |
| DiLazzaro. V^88^ | cTBS | affected hemisphere | MagPro | figure-of-eight | 80% motor threshold | 600 | 50 | no information | no information | 10 |
| Seniow. J^47^ | lfTMS | unaffected hemisphere | MagStim | figure-of-eight | 90% motor threshold | 1,800 | 1 | no information | 30 min | 15 |
| Conforto. AB^62^ | lfTMS | unaffected hemisphere | MagPro | figure-of-eight | 90% motor threshold | 1,500 | 1 | no information | 25 min | 10 |
| Sohn. MK^63^ | hfTMS | affected hemisphere | MagPro | figure-of-eight | 100% motor threshold | 1,000 | 10 | 10/50 | no information | 10 |
| Malcolm. MP^89^ | hfTMS | affected hemisphere | MagStim | figure-of-eight | 90% motor threshold | 2,000 | 20 | 2/28 | no information | 10 |
| Chen. X^9^ | lfTMS | unaffected hemisphere | MagVenture | figure-of-eight | no information | 1,500 | 1 | no information | no information | 10 |
| Khan. F^37^ | iTBS+cTBS | unaffected and affected hemisphere | MagStim | figure-of-eight | 60% motor threshold | 1,200 | no information | no information | no information | 12 |
| Tosun. A^50^ | lfTMS | unaffected hemisphere | Remed | figure-of-eight | 90% motor threshold | 1,200 | 1 | no information | 20 min | 10 |
| Du. J^26^ | lfTMS | unaffected hemisphere | CCY-I | no information | 90% motor threshold | 1,200 | 1 | no information | 20 min | 20 |
| Tarri. M^48^ | no information | affected hemisphere | MagVenture | figure-of-eight | no information | no information | no information | no information | 30 min | 5 |
| Blesneag. AV^53^ | lfTMS | unaffected hemisphere | MagVenture | figure-of-eight | 120% motor threshold | 1,200 | 1 | no information | 20 min | no information |

Abbreviations: cTBS, continuous theta burst stimulation; hfTMS, high frequency transcranial magnetic stimulation; iTBS, intermittent theta burst stimulation; lfTMS, low frequency transcranial magnetic stimulation.

**D)** rPMS

| **Author** | **Sub type** | **Site** | **Brand** | **Coil** | **Intensity** | **Pulse** | **Frequency (Hz)** | **On/off time (sec)** | **Duration** | **No. of session** |
| --- | --- | --- | --- | --- | --- | --- | --- | --- | --- | --- |
| Ke. J^11^ | rpms | axillar, popliteal fossa | CCY-IV | figure-of-eight | 40-60% of max output | 1,800 | 20 | 1/19 | 30 min | 10 |
| Jiang. YF^10^ | rpms | triceps, EDC | Magneuro | circular | 15-30% of maximum output | 2,400 | 20 | 0.5/2 | no information | 14 |
| El Nahas.N^60^ | p-iTBS | bicep, wrist flexor muscles | MagVenture | figure-of-eight | supra motor threshold | 600 | no information | no information | no information | 8 |
| Krewer. C^39^ | rpms | extensor and flexor of upper arm and lower arm | P-Stim 160 | figure-of-eight | 110 % motor threshold | 5,000 | 25 | 1/2 | 20 min | 20 |
| Chen. X^9^ | rpms | extensor muscles of upper extremity and shoulder muscles | MagVenture | parabolic | 20-40% maximum output | no information | 30 Hz | no information | no information | 10 |

Abbreviations: EDC, extensor digitorum communis; p-iTBS, peripheral intermittent theta burst stimulation.

**Appendix 5:** Results of risk of bias assessment


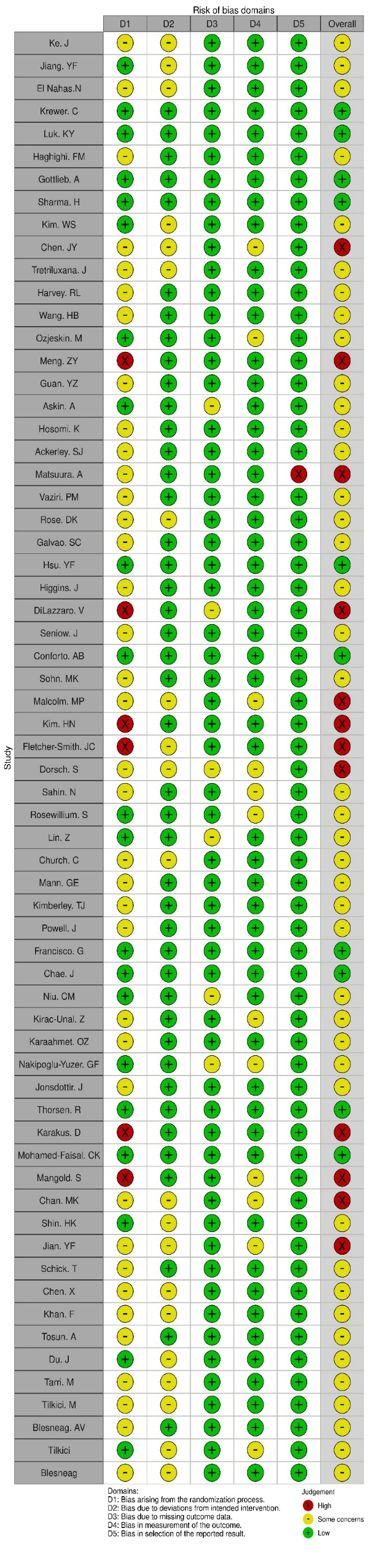

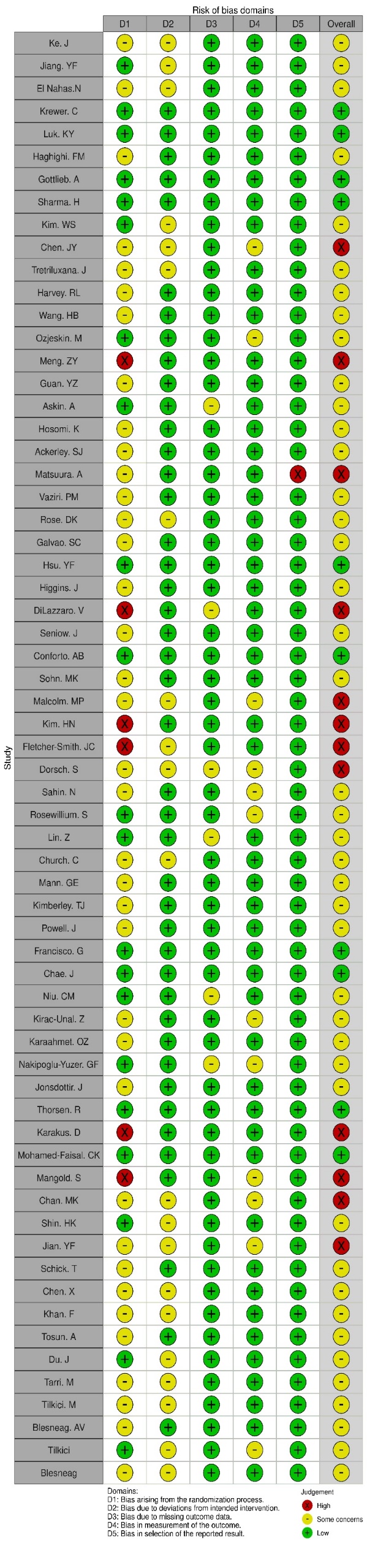

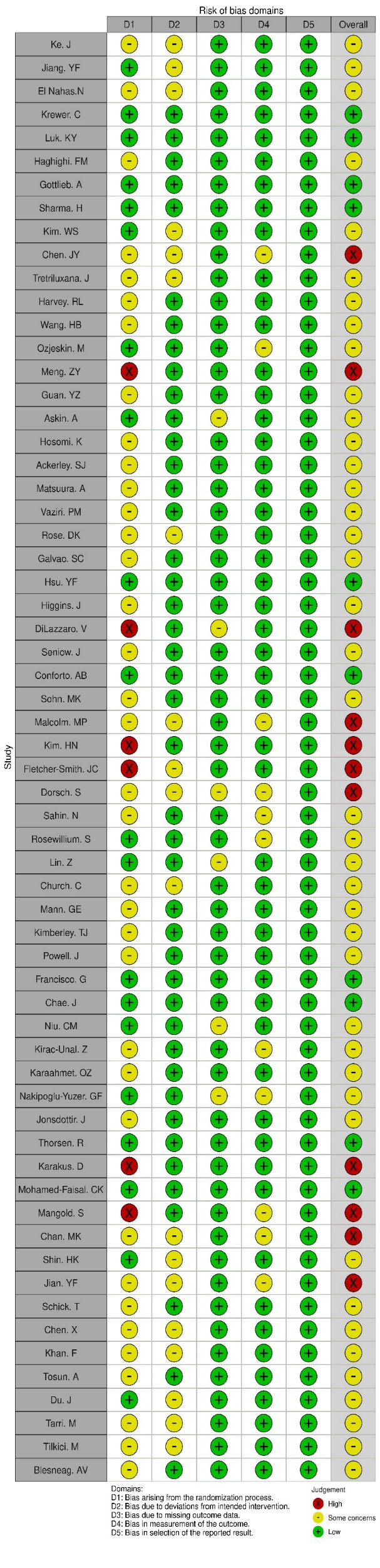


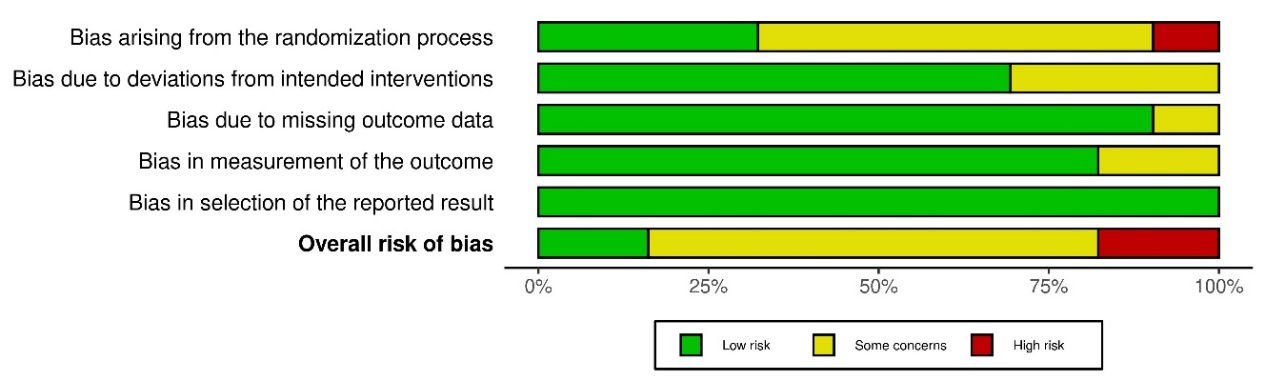


**Appendix 6:** Pooled intervention effects stratified by evaluation time: Pairwise meta-analysis

| 1. **At the end of intervention course** | | | | | | |
| --- | --- | --- | --- | --- | --- | --- |
| **Treatment comparisons** | | **No. of studies** | **USMD** | **95% CI** | **I2 (%)** | **Egger’s test** |
| **Intervention** | **Comparator** |  |  |  |  |  |
| Upper extremity function | | | | | | |
| NMES | Rehab | 5 | 7.28 | 2.68, 11.88 | 83.26 | 0.68 |
| - Acute-subacute stroke | | 3 | 6.33 | 3.03, 9.62 | 0 | - |
| - Chronic stroke | | 2 | 7.47 | -1.22, 16.16 | 95.67 |  |
| - More severe stroke (FMA ≤ 10) | | 3 | 9.06 | 4.30, 13.82 | 69.03 |  |
| - Less severe stroke (FMA > 10) | | 2 | 3.20 | 1.41, 4.98 | 0 |  |
| FES | Rehab | 6 | 5.37 | 1.25, 9.49 | 70.96 | 0.15 |
| - Acute-subacute stroke | | 2 | 5.75 | -5.92, 17.43 | 65.53 | - |
| - Chronic stroke | | 3 | 3.48 | -2.37, 9.32 | 0 |  |
| - More severe stroke (FMA < 25) | | 4 | 5.72 | 0.94, 10.50 | 80.82 |  |
| - Less severe stroke (FMA ≥25) | | 2 | 3.15 | -5.12, 11.42 | 0 |  |
| - Intervention ≤ 20 day | | 4 | 6.68 | 1.45, 11.90 | 41.78 |  |
| - Intervention > 20 day | | 2 | 4.02 | 1.31, 6.73 | 0 |  |
| TMS | Rehab | 19 | 2.97 | 0.30, 5.64 | 73.78 | 0.73 |
| - Acute-subacute stroke | | 9 | 4.86 | 0.92, 8.80 | 70.59 | - |
| - Chronic stroke | | 8 | 1.62 | -0.72, 4.03 | 28.72 |  |
| - More severe stroke (FMA < 25) | | 5 | 4.73 | 0.17, 9.29 | 87.97 |  |
| - Less severe stroke (FMA $\geq$ 25) | | 14 | 1.61 | -0.46, 3.67 | 4.47 |  |
| - Low frequency TMS | | 13 | 2.81 | 1.09, 4.54 | 14.40 |  |
| - High frequency TMS | | 3 | -1.06 | -5.58, 3.45 | 0 |  |
| - iTBS | | 2 | 6.72 | -4.21, 17.65 | 0 |  |
| ADL | | | | | | |
| NMES | Rehab | 3 | -0.21 | -1.86, 1.43 | 0 | 0.72 |
| TMS | Rehab | 5 | 7.94 | 0.44, 15.44 | 79.11 | 0.04 |
| - Acute-subacute stroke | | 3 | 14.89 | 0.45, 29.33 | 86.72 | - |
| - More severe stroke (FMA < 25) | | 2 | 6.22 | -7.29, 19.74 | 76.14 |  |
| - Less severe stroke (FMA $\geq$ 25) | | 3 | 10.24 | -1.48, 21.96 | 84.63 |  |
| - Low frequency TMS | | 3 | 11.80 | -4.93, 28.53 | 85.07 |  |
| Spasticity | | | | | | |
| NMES | Rehab | 4 | -0.13 | -0.64, 0.39 | 83.95 | < 0.001 |
| - Acute-subacute stroke | | 2 | -0.13 | -1.15, 0.90 | 90.89 | - |
| - Chronic stroke | | 2 | -0.19 | -1.19, 0.82 | 86.81 |  |
| FES | Rehab | 3 | 0.08 | -0.31, 0.46 | 0 | 0.22 |
| - Acute-subacute stroke | | 2 | 0.20 | -0.24, 0.63 | 0 | - |
| TMS | Rehab | 8 | 0.00 | -0.35, 0.35 | 72.43 | 0.67 |
| - Acute-subacute stroke | | 3 | -0.11 | -0.49, 0.26 | 5.61 | - |
| - Chronic stroke | | 5 | 0.00 | -0.47, 0.48 | 80.38 |  |
| - More severe stroke (FMA < 25) | | 3 | 0.19 | -0.27, 0.65 | 70.96 |  |
| - Less severe stroke (FMA $\geq$ 25) | | 2 | 0.21 | -0.47, 0.91 | 48.83 |  |
| - Low frequency TMS | | 7 | 0.13 | -0.18, 0.43 | 58.03 |  |
| 1. **Follow-up at 1 – 3 months** | | | | | | |
| Upper extremity function | | | | | | |
| TMS | Rehab | 9 | 3.55 | 1.04, 6.05 | 16.23 | 0.07 |
| - Acute-subacute stroke | | 7 | 3.95 | 1.31, 6.59 | 20.88 | - |
| - Chronic stroke | | 2 | -0.11 | -8.06, 7.84 | 5.98 |  |
| - Low frequency TMS | | 7 | 3.63 | 0.99, 6.27 | 31.35 |  |
| Spasticity | | | | | | |
| TMS | Rehab | 3 | -0.05 | -0.35, 0.24 | 0 | 0.63 |
| - Chronic stroke | | 2 | -0.14 | -0.47, 0.19 | 0 | - |

Abbreviations: FES, functional electrical stimulation; NMES, neuromuscular electrical stimulation; Rehab, conventional rehabilitation; rPMS, repetitive electrical magnetic stimulation; TMS, transcranial magnetic stimulation; iTBS, intermittence theta burst stimulation.

**Appendix 7:** Subgroup analyses of NMA on the upper extremity functions outcome measured with FMA at the end of intervention course

1.
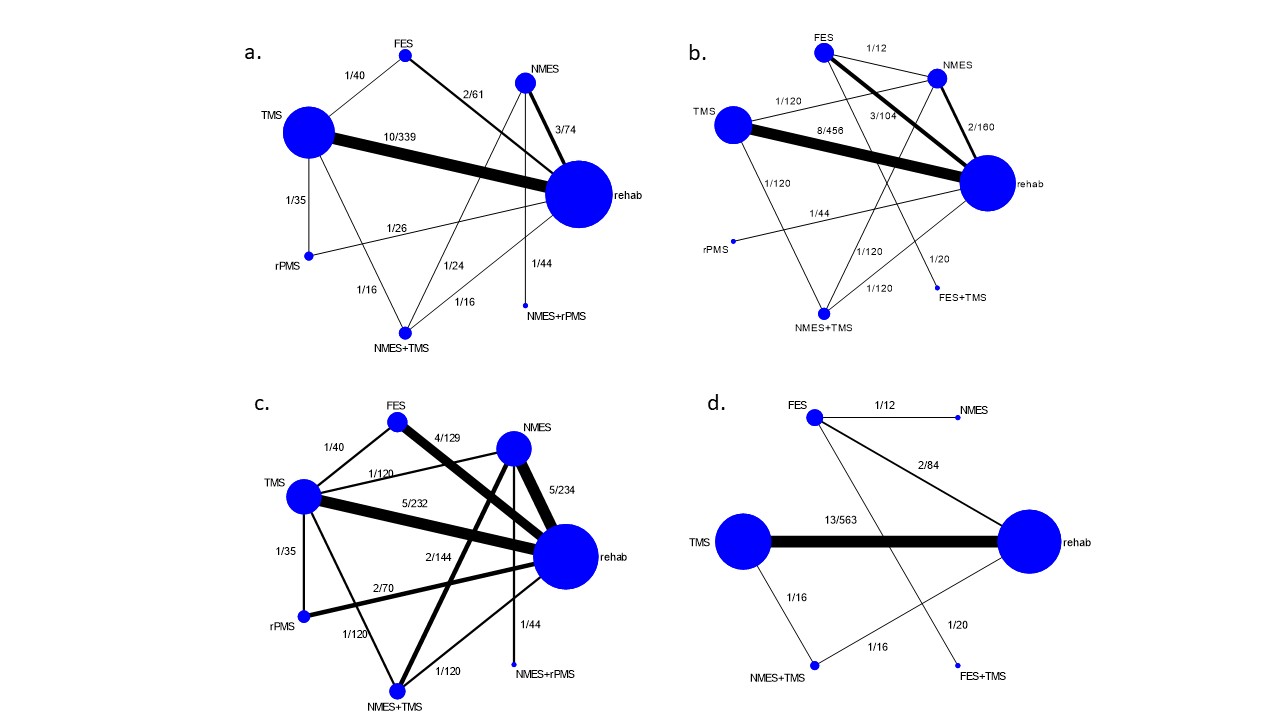
Network maps


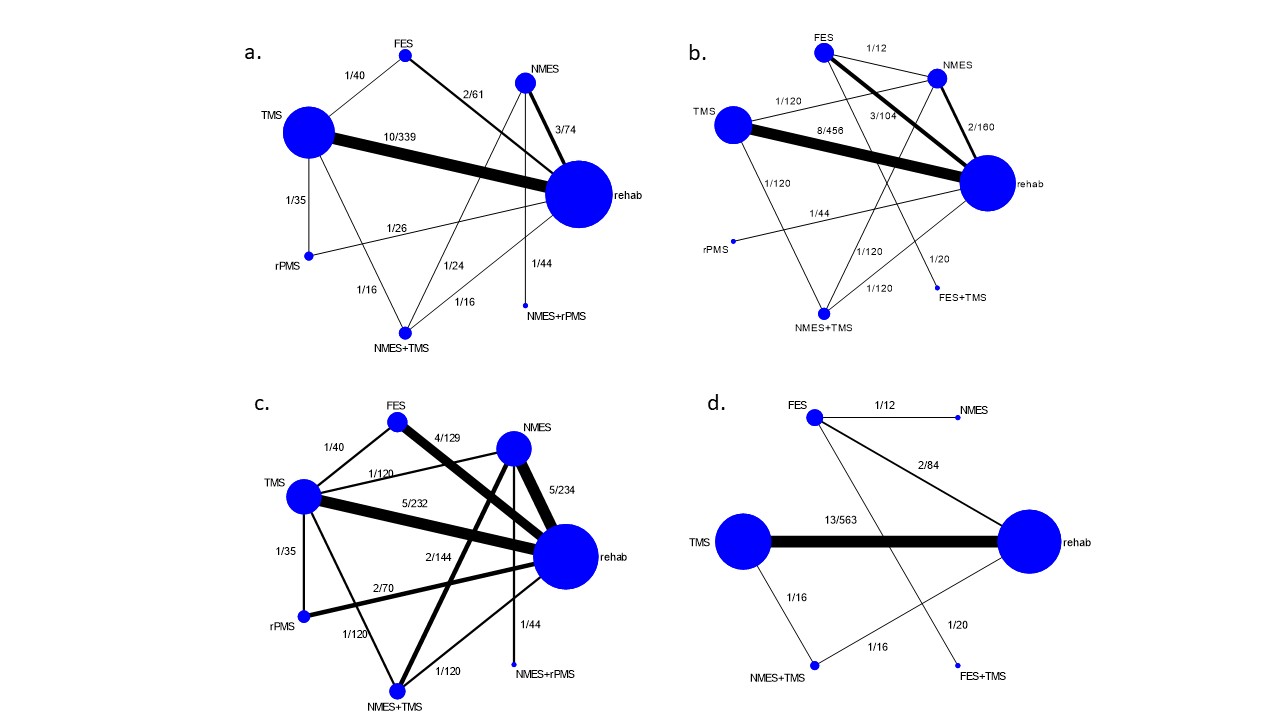


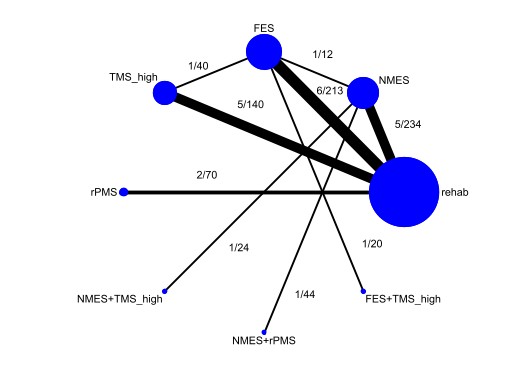

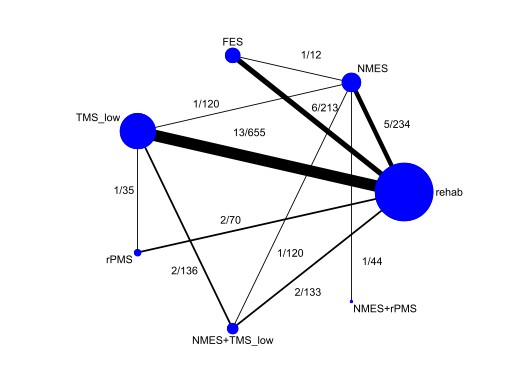

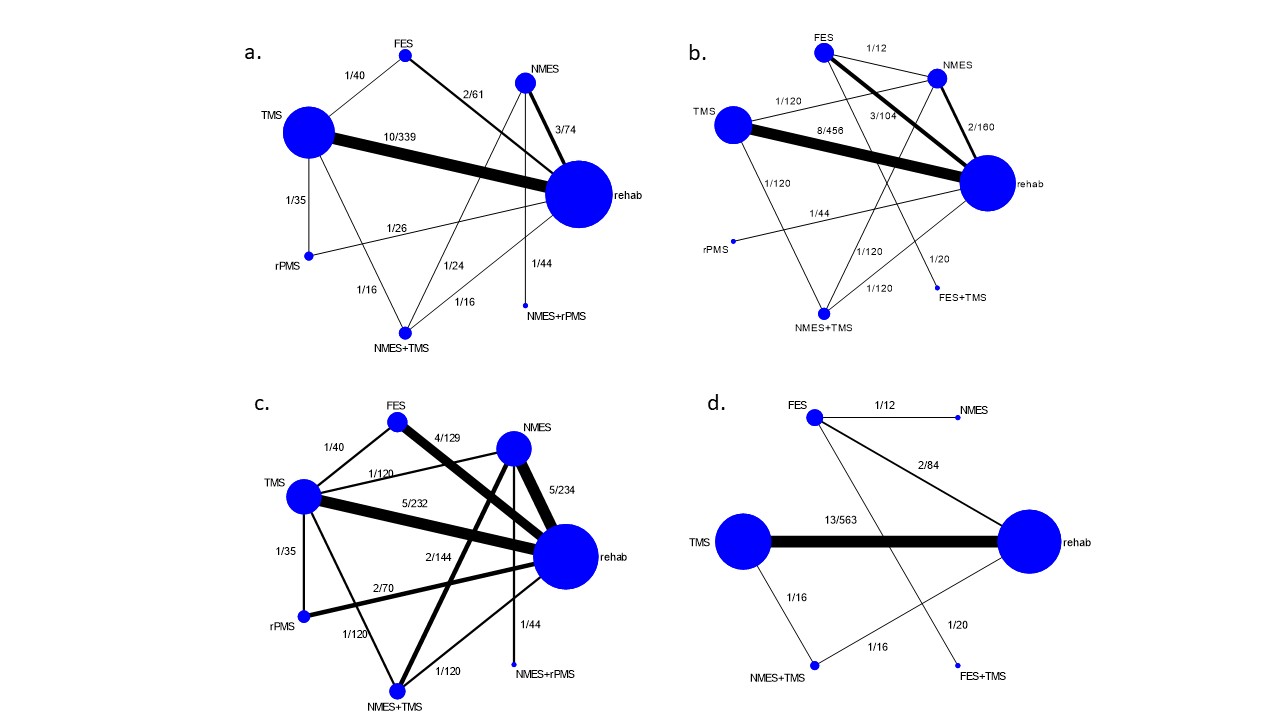


f.

e.

(a.) acute-subacute stroke subgroup, (b.) chronic stroke subgroup, (c.) more severe stroke subgroup, (d.) less severe stroke subgroup, (e.) low-frequency TMS subgroup, and (f.) high-frequency TMS subgroup, with nodes and edges weighted by the number of studies and included patients, respectively

**B)** Multiple treatment comparisons of interventions on upper extremity functions measured with FMA in acute-sub-acute stroke

| **Reference treatment** | **Mean difference** | | | | | | |  |  |
| --- | --- | --- | --- | --- | --- | --- | --- | --- | --- |
|  | **Rehab** | **NMES+rPMS** | **NMES+TMS** | **rPMS** | **TMS** | **FES** | **NMES** |  | |
| Rehab | 29.6, 0.0 | 11.93 (8.27, 15.59) | -3.2 (-19.97, 13.57) | -0.73, (-16.14, 14.68) | 10.60 (8.87, 12.33) | -2.7 (-17.80, 12.40) | 6.33 (3.04, 9.62) | |  |
| NMES+rPMS | -11.93 (-15.59,  -8.27) | 92.8, 64.1 | -15.13 (-32.29, 2.04) | -12.66 (-28.50, 3.18) | -1.33 (-5.38, 2.72) | -14.63 (-30.16, 0.91) | -5.60 (-7.20, -4.00) | |  |
| NMES+TMS | 3.2 (-14.68, 16.14) | 15.13 (-2.04, 32.29) | 25.6, 3.9 | 2.47 (-20.31, 25.25) | 13.80 (-3.06, 30.66) | 0.5 (-22.07, 23.07) | 9.53 (-7.56, 26.62) | |  |
| rPMS | 0.73 (-14.68, 16.14) | 12.66 (-3.18, 28.50) | -2.47 (-25.25, 20.31) | 34.0, 6.6 | 11.33 (-4.18, 26.84) | -1.97 (-23.55, 19.61) | 7.06 (-8.70, 22.82) | |  |
| TMS | -10.60 (-12.33,  -8.87) | 1.33 (-2.72, 5.38) | -13.80 (-30.66, 3.06) | -11.33 (-26.84, 4.18) | 84.4, 22.5 | -14.30 (-28.50, 1.90) | -4.27 (-7.99, -0.55) | |  |
| FES | 2.7 (-12.40, 17.80) | 14.63 (-0.91, 30.16) | -0.5 (-23.07, 22.07) | 1.97 (-19.61, 23.55) | 14.30 (-1.90, 28.50) | 24.7, 2.9 | 9.03 (-6.42, 24.48) | |  |
| NMES | -6.33 (-9.62, -3.04) | 5.60 (4.00, 7.20) | -9.53 (-26.62, 7.56) | -7.06 (-22.82, 8.70) | 4.27 (0.55, 7.99) | -9.03 (-24.48, 6.42) | 58.9, 0.0 | |  |

Abbreviations: FES, functional electrical stimulation; NMES, neuromuscular electrical stimulation; Rehab, conventional rehabilitation; rPMS, repetitive electrical magnetic stimulation; TMS, transcranial magnetic stimulation.

Results in the off-diagonal cells are the mean differences and 95% confidence intervals of the FMA from the network meta-analysis. Each diagonal cell contains the surface under the cumulative ranking and probability of being the best treatment of each intervention.

**C)** Multiple treatment comparisons of interventions on upper extremity functions measured with FMA in chronic stroke

| **Reference treatment** | **Mean difference** | | | | | | |  |  |
| --- | --- | --- | --- | --- | --- | --- | --- | --- | --- |
|  | **Rehab** | **FES+TMS** | **NMES+TMS** | **rPMS** | **TMS** | **FES** | **NMES** |  | |
| Rehab | 13.5, 0.0 | 0.24 (-10.53, 11.01) | 6.10 (3.50, 8.70) | 4.34 (-4.09, 12.77) | 4.07 (1.27, 6.87) | 3.44 (-2.59, 9.47) | 12.00 (8.22, 15.77) | |  |
| FES+TMS | -0.24 (-11.01, 10.53) | 22.8, 5.6 | 5.86 (-5.22, 16.94) | 4.10 (-9.58, 17.78) | 3.83 (-7.30, 14.96) | 3.20 (-5.72, 12.12) | 11.76 (0.35, 23.17) | |  |
| NMES+TMS | -6.10 (-8.70, -3.50) | -5.86 (-16.94, 5.22) | 71.0, 0.5 | -1.76 (-10.58, 7.06) | -2.03 (-4.80, 0.74) | -2.66 (-9.23, 3.91) | 5.90 (1.31, 10.48) | |  |
| rPMS | -4.34 (-12.77, 4.09) | -4.10 (-17.78, 9.58) | 1.76 (-7.06, 10.58) | 51.2, 5.0 | -0.27 (-9.15, 8.61) | -0.90 (-11.27, 9.47) | 7.66 (-1.58, 16.90) | |  |
| TMS | -4.07 (-6.87, -1.27) | -3.83 (-14.96, 7.30) | 2.03 (-0.74, 4.80) | 0.27 (-8.61, 9.15) | 48.5, 0.0 | -0.63 (-7.28, 6.02) | 7.93 (3.23, 12.63) | |  |
| FES | -3.44 (-9.47, 2.59) | -3.20 (-12.12, 5.72) | 2.66 (-3.91, 9.23) | 0.90 (-9.47, 11.27) | 0.63 (-6.02, 7.28) | 44.5, 1.0 | 8.56 (1.45, 15.68) | |  |
| NMES | -12.00 (-15.77,  -8.22) | -11.76 (-23.17, -0.35) | -5.90, (-10.48, -1.31) | -7.66 (-16.90, 1.58) | -7.93 (-12.63, -3.23) | -8.56 (-15.68, -1.45) | 98.5, 91.6 | |  |

Abbreviations: FES, functional electrical stimulation; NMES, neuromuscular electrical stimulation; Rehab, conventional rehabilitation; rPMS, repetitive electrical magnetic stimulation; TMS, transcranial magnetic stimulation.

Results in the off-diagonal cells are the mean differences and 95% confidence intervals of the FMA from the network meta-analysis. Each diagonal cell contains the surface under the cumulative ranking and probability of being the best treatment of each intervention.

**D)** Multiple treatment comparisons of interventions on upper extremity functions measured with FMA in the more severe (FMA baseline < 25) subgroup

| **Reference treatment** | **Mean difference** | | | | | | |  |  |
| --- | --- | --- | --- | --- | --- | --- | --- | --- | --- |
|  | **Rehab** | **NMES+rPMS** | **NMES+TMS** | **rPMS** | **TMS** | **FES** | **NMES** |  | |
| Rehab | 5.0, 0.0 | 14.63 (8.87, 20.40) | 6.10 (1.56, 10.64) | 3.08 (-4.92, 11.07) | 4.07 (-0.59, 8.73) | 3.57 (-0.79, 7.93) | 9.04 (5.51, 12.56) | |  |
| NMES+rPMS | -14.63 (-20.40,  -8.87) | 99.2, 96.4 | -8.53 (-15.87, -1.19) | -11.56 (-21.40,  -1.71) | -10.56 (-17.97,  -3.15) | -11.06 (-18.26,  -3.85) | -5.60 (-10.16, 1.03) | |  |
| NMES+TMS | -6.10 (-10.64, -1.56) | 8.53 (1.19, 15.87) | 57.9, 1.4 | -3.02 (-12.22, 6.17) | -2.03 (-6.67, 2.61) | -2.52 (-8.82, 3.77) | 2.94 (-2.81, 8.68) | |  |
| rPMS | -3.08 (-11.07, 4.92) | 11.56 (1.71, 21.40) | 3.02 (-6.17, 12.22) | 34.0, 0.9 | 0.99 (-8.26, 10.25) | 0.50 (-8.59, 9.58) | 5.96 (-2.77, 14.69) | |  |
| TMS | -4.07 (-8.73, 0.59) | 10.56 (3.15, 17.97) | 2.03 (-2.61, 6.67) | -0.99 (-10.25, 8.26) | 39.4, 0.2 | -0.50 (-6.88, 5.88) | 4.97 (-0.88, 10.81) | |  |
| FES | -3.57 (7.93, 0.79) | 11.06 (3.85, 18.26) | 2.52 (-3.77, 8.82) | -0.50 (-9.58, 8.59) | 0.50 (-5.88, 6.88) | 36.8, 0.2 | 5.46 (-0.11, 11.04) | |  |
| NMES | -9.04 (-12.56, -5.51) | 5.60 (1.03, 10.16) | -2.94 (-8.68, 2.81) | -5.96 (-14.69, 2.77) | -4.97 (-10.81, 0.88) | -5.46 (-11.04, 0.11) | 77.6, 0.9 | |  |

Abbreviations: FES, functional electrical stimulation; NMES, neuromuscular electrical stimulation; Rehab, conventional rehabilitation; rPMS, repetitive electrical magnetic stimulation; TMS, transcranial magnetic stimulation.

Results in the off-diagonal cells are the mean differences and 95% confidence intervals of the FMA from the network meta-analysis. Each diagonal cell contains the surface under the cumulative ranking and probability of being the best treatment of each intervention.

**E)** Multiple treatment comparisons of interventions on upper extremity functions measured with FMA in the less severe (baseline FMA ≥ 25) subgroup

| **Reference treatment** | **Mean difference** | | | | | |  |  |
| --- | --- | --- | --- | --- | --- | --- | --- | --- |
|  | **Rehab** | **FES+TMS** | **NMES+TMS** | **TMS** | **FES** | **NMES** |  | |
| Rehab | 39.6, 0.9 | -0.12 (-12.63, 12.39) | -3.2 (-20.18, 13.78) | 1.54 (-0.75, 3.83) | 3.08 (-5.54, 11.70) | 0.08 (-13.11, 13.27) | |  |
| FES+TMS | 0.12 (-12.39, 20.18) | 45.5, 15.4 | -3.08 (-24.17, 18.01) | 1.66 (-11.06, 14.38) | 3.20 (-5.87, 12.27) | 0.20 (-13.30, 13.71) | |  |
| NMES+TMS | 3.2 (-13.78, 20.18) | 3.08 (-18.01, 24.17) | 34.9, 19.9 | 4.74 (-12.40, 21.87) | 6.28 (-12.76, 25.32) | 3.28 (-18.22, 24.78) | |  |
| TMS | -1.54 (-3.83, 0.75) | -1.66 (-14.38, 11.06) | -4.74 (-21.87, 12.40) | 62.1, 18.7 | 1.54 (-7.38, 10.46) | -1.46 (-14.85, 11.93) | |  |
| FES | -3.08 (-11.70, 5.54) | -3.20 (-12.27, 5.87) | -6.28 (-25.32, 12.76) | -1.54 (-10.46, 7.38) | 71.9, 27.3 | -3.00 (-13.01, 7.01) | |  |
| NMES | -0.08 (-13.27, 13.11) | -0.20 (-13.71, 13.30) | -3.28 (-24.78, 18.22) | 1.46 (-11.93, 14.85) | 3.00 (-7.01, 13.01) | 46.0, 17.8 | |  |

Abbreviations: FES, functional electrical stimulation; NMES, neuromuscular electrical stimulation; Rehab, conventional rehabilitation; rPMS, repetitive electrical magnetic stimulation; TMS, transcranial magnetic stimulation.

Results in the off-diagonal cells are the mean differences and 95% confidence intervals of the FMA from the network meta-analysis. Each diagonal cell contains the surface under the cumulative ranking and probability of being the best treatment of each intervention.

**F)** Multiple treatment comparisons of interventions on upper extremity functions measured with FMA in low-frequency TMS subgroup

| **Reference treatment** | **Mean difference** | | | | | | |  |  |
| --- | --- | --- | --- | --- | --- | --- | --- | --- | --- |
|  | **Rehab** | **NMES+rPMS** | **NMES+TMS_low** | **rPMS** | **TMS_low** | **FES** | **NMES** |  | |
| Rehab | 5.4, 0.0 | 14.60 (8.25, 20.96) | 6.10 (1.01, 11.19) | 3.04 (-5.15, 11.23) | 4.07 (-1.12, 9.26) | 6.00 (2.63, 9.37) | 9.01 (5.23, 12.78) | |  |
| NMES+rPMS | -14.60 (-20.96,  -8.25) | 99.0, 95.6 | -8.50 (-16.64, -0.36) | -11.57 (-21.93,  -1.20) | -10.53 (-18.74, 2.33) | -8.60 (-15.77,  -1.43) | -5.60 (-10.70, -0.49) | |  |
| NMES+TMS_low | -6.10 (-11.19,  -1.01) | 8.50 (0.36, 16.64) | 53.8, 1.6 | -3.06 (-12.71, 6.58) | -2.03 (-7.21, 3.15) | -0.98 (-6.20, 6.01) | 2.91 (3.43, 9.25) | |  |
| rPMS | -3.04 (-11.23, 5.15) | 11.57 (1.20, 21.93) | 3.06 (-6.58, 12.71) | 30.2, 1.2 | 1.03 (-8.67, 10.73) | 2.96 (-5.87, 11.80) | 5.97 (-3.05, 14.99) | |  |
| TMS_low | -4.07 (-9.26, 1.12) | 10.53 (2.33, 18.74) | 2.03 (-3.15, 7.21) | -1.03 (-10.73, 8.67) | 34.2, 0.6 | 1.93 (-4.26, 8.12) | 4.94 (-1.48, 11.36) | |  |
| FES | -6.00 (-9.37,  -2.63) | 8.60 (1.43, 15.77) | 0.98 (-6.01, 6.20) | -2.96 (-11.80, 5.87) | -1.93 (-8.12, 4.26) | 51.5, 0.4 | 3.01 (-2.03, 8.04) | |  |
| NMES | -9.01 (-12.78,  -5.23) | 5.60 (0.49, 10.70) | -2.91 (-9.25, 3.43) | -5.97 (-14.99, 3.05) | -4.94 (-11.36, 1.48) | -3.01 (-8.04, 2.03) | 76.0, 0.6 | |  |

Abbreviations: FES, functional electrical stimulation; NMES, neuromuscular electrical stimulation; Rehab, conventional rehabilitation; rPMS, repetitive electrical magnetic stimulation; TMS_low, low frequency transcranial magnetic stimulation.

Results in the off-diagonal cells are the mean differences and 95% confidence intervals of the FMA from the network meta-analysis. Each diagonal cell contains the surface under the cumulative ranking and probability of being the best treatment of each intervention.

**G)** Multiple treatment comparisons of interventions on upper extremity functions measured with FMA in high-frequency TMS subgroup

| **Reference treatment** | **Mean difference** | | | | | | | |  |  |
| --- | --- | --- | --- | --- | --- | --- | --- | --- | --- | --- |
|  | **Rehab** | **FES+TMS_high** | **NMES+rPMS** | **NMES+TMS_high** | **rPMS** | **TMS_high** | **FES** | **NMES** |  | |
| Rehab | 14.0, 0.0 | 0.15 (-11.13, 11.42) | 12.66 (5.87, 19.44) | 6.37 (-8.65, 21.40) | 2.98 (-5.52, 11.48) | 10.6 (4.70, 16.50) | 3.35 (-1.13, 7.82) | 7.06 (3.64, 10.48) | |  |
| FES+TMS_high | -0.15 (-11.42, 11.33) | 22.9, 1.4 | 12.51 (-0.06, 25.68) | 6.23 (-12.57, 25.02) | 2.84 (-11.27, 16.95) | 10.45 (-2.27, 23.18) | 3.20 (-7.15, 13.55) | 6.92 (-4.88, 18.71) | |  |
| NMES_rPMS | -12.66 (-19.44, -5.87) | -12.51 (-25.68, 0.66) | 90.3, 52.1 | -6.28 (-22.05, 9.48) | -9.68 (-20.56, 1.21) | -2.06 (-11.05, 6.94) | -9.31 (-17.46, -1.16) | -5.59 (-11.46, 0.27) | |  |
| NMES+TMS_high | -6.37 (-21.40, 8.65) | -6.23 (-25.02, 12.57) | 6.28 (-9.48, 22.05) | 55.2, 17.8 | -3.39 (-20.66, 13.88) | 4.28 (-11.92, 20.37) | -3.03 (-18.72, 12.66) | 0.69 (-13.94, 15.32) | |  |
| rPMS | -2.98 (-11.48, 5.52) | -2.84 (-16.95, 11.27) | 9.68 (-1.21, 20.56) | 3.39 (-13.88, 20.66) | 36.1, 1.4 | 7.62 (-2.73, 17.97) | 0.36 (-9.23, 9.96) | 4.08 (-5.09, 13.26) | |  |
| TMS_high | -10.6 (-16.50, -4.70) | -10.45 (-23.18, 2.27) | 2.06 (-6.94, 11.05) | -4.28 (-20.37, 11.92) | -7.62 (-17.97, 2.73) | 82.1, 27.7 | -7.25 (14.66, 0.15) | -3.54 (-10.36, 3.28) | |  |
| FES | -3.35 (-7.82, 1.13) | -3.20 (-13.55, 7.15) | 9.31 (1.16, 17.46) | 3.03 (-12.66, 18.72) | -0.36 (-9.96, 9.23) | 7.25 (-0.15, 14.66) | 38.7, 0.1 | 3.72 (-1.94, 9.38) | |  |
| NMES | -7.06 (-10.48, - 3.64) | -6.92 (-18.71, 4.88) | 5.59 (-0.27, 11.46) | -0.69 (-15.32, 13.94) | -4.08 (-13.26, 5.09) | 3.54 (-3.28, 10.36) | -3.72 (-9.38, 1.94) | 60.8, 0.1 | |  |

Abbreviations: FES, functional electrical stimulation; NMES, neuromuscular electrical stimulation; Rehab, conventional rehabilitation; rPMS, repetitive electrical magnetic stimulation; TMS_high, high frequency transcranial magnetic stimulation.

Results in the off-diagonal cells are the mean differences and 95% confidence intervals of the FMA from the network meta-analysis. Each diagonal cell contains the surface under the cumulative ranking and probability of being the best treatment of each intervention.

**Appendix 8: Consistency assumption**

1. **FMA**

Inconsistency factors estimated by using the loop-specific approach for FMA at the end of intervention course

| **Loop** | **Inconsistency factor (95%CI)** | **Comparison examined** | **Direct estimated, MD(SD)** | **Indirect estimate, MD(SD)** |
| --- | --- | --- | --- | --- |
| NMES – NMES+TMS – Rehab | 6.19 (0.00, 13.98) | NMES – NMES+TMS | 2.56 (2.46) | -3.63 (3.12) |
| TMS – NMES+TMS – Rehab | 6.16 (0.00, 19.81) | TMS – NMES+TMS | -4.83 (4.66) | 1.33 (5.18) |
| TMS – rPMS – Rehab | 5.66 (0.00, 21.17) | TMS – rPMS | 5.17 (6.07) | -0.49 (5.07) |
| NMES – TMS – Rehab | 5.10 (0.00, 16.32) | NMES - TMS | 0.94 (4.88) | -4.16 (3.00) |
| NMES – FES – Rehab | 4.99 (0.00, 18.88) | NMES – FES | 3.00 (6.34) | -1.99 (3.16) |
| FES – TMS – Rehab | 4.42 (0.00, 10.21) | FES – TMS | 0.65 (2.31) | -3.77 (1.85) |
| NMES – TMS – NMES+TMS | 3.21 (0.00, 16.98) | TMS – NMES+TMS | 1.58 (0.92) | -1.63 (6.97) |
| NMES – FES – TMS | 2.71 (0.00, 12.68) | FES – TMS | 0.65 (0.69) | -2.06 (5.04) |

Abbreviations: FES, functional electrical stimulation; NMES, neuromuscular electrical stimulation; Rehab, conventional rehabilitation; rPMS, repetitive electrical magnetic stimulation; TMS, transcranial magnetic stimulation.

1. **BI**

Inconsistency factors estimated by using the loop-specific approach for BI at the end of intervention course

| **Loop** | **Inconsistency factor (95%CI)** | **Comparison examined** | **Direct estimated, MD(SD)** | **Indirect estimate, MD(SD)** |
| --- | --- | --- | --- | --- |
| TMS – NMES+TMS – Rehab | 37.80 (3.69, 71.91) | TMS – NMES+TMS | -11.90 (9.28) | 25.90 (14.72) |
| TMS – rPMS – Rehab | 22.02 (0.00, 68.46) | TMS – rPMS | 9.41 (14.95) | -12.61 (18.38) |
| TMS – FES – Rehab | 4.63 (0.00, 14.81) | TMS – FES | 0.75 (11.07) | -3.88 (9.84) |

Abbreviations: FES, functional electrical stimulation; NMES, neuromuscular electrical stimulation; Rehab, conventional rehabilitation; rPMS, repetitive electrical magnetic stimulation; TMS, transcranial magnetic stimulation.

**Appendix 9:** Final rating of confidence in network meta-analysis according to the framework of CINeMA

**A)** Upper extremity functions with FMA

| **Comparison** | **Number of studies** | **Within-study bias** | **Reporting bias** | **Indirectness** | **Imprecision** | **Heterogeneity** | **Incoherence** | **Confidence rating** | **Reasons for downgrading** |
| --- | --- | --- | --- | --- | --- | --- | --- | --- | --- |
| Mixed Evidence | | | | | | | | | |
| FES:FES+TMS | 1 | Some concerns | Low risk | No concerns | Some concerns | No concerns | Major concerns | Very low | Within-study bias, Imprecision, Incoherence |
| FES:NMES | 1 | Some concerns | Low risk | No concerns | No concerns | No concerns | No concerns | Moderate | Within-study bias |
| FES:Rehab | 6 | Some concerns | Low risk | No concerns | No concerns | Some concerns | No concerns | Low | Within-study bias, Heterogeneity |
| FES:TMS | 1 | Some concerns | Low risk | No concerns | No concerns | No concerns | No concerns | Moderate | Within-study bias |
| NMES:NMES+rPMS | 1 | Some concerns | Low risk | No concerns | Some concerns | No concerns | Major concerns | Very low | Within-study bias, Imprecision, Incoherence |
| NMES:NMES+TMS | 2 | Some concerns | Low risk | No concerns | No concerns | No concerns | Some concerns | Moderate | Within-study bias, Incoherence |
| NMES:Rehab | 5 | Some concerns | Low risk | No concerns | No concerns | Some concerns | No concerns | Low | Within-study bias, Heterogeneity |
| NMES:TMS | 1 | Some concerns | Low risk | No concerns | No concerns | No concerns | No concerns | Moderate | Within-study bias |
| NMES+TMS:Rehab | 2 | Some concerns | Low risk | No concerns | No concerns | Some concerns | No concerns | Low | Within-study bias, Heterogeneity |
| NMES+TMS:TMS | 2 | Some concerns | Low risk | No concerns | No concerns | No concerns | No concerns | Moderate | Within-study bias |

Abbreviations: FES, functional electrical stimulation; NMES, neuromuscular electrical stimulation; Rehab, conventional rehabilitation; rPMS, repetitive electrical magnetic stimulation; TMS, transcranial magnetic stimulation.

**A)** Upper extremity functions with FMA (Cont.)

| **Comparison** | | **Number of studies** | **Within-study bias** | **Reporting bias** | **Indirectness** | **Imprecision** | **Heterogeneity** | **Incoherence** | **Confidence rating** | **Reasons for downgrading** |
| --- | --- | --- | --- | --- | --- | --- | --- | --- | --- | --- |
|  | Mixed Evidence | | | | | | | | | |
| RehabRehab:rPMS | | 2 | Some concerns | Low risk | No concerns | No concerns | Some concerns | No concerns | Low | Within-study bias, Heterogeneity |
| RehabRehab:TMS | | 18 | Some concerns | Low risk | No concerns | No concerns | No concerns | No concerns | Moderate | Within-study bias |
| rPMS:TMS | | 1 | Some concerns | Low risk | No concerns | No concerns | No concerns | No concerns | Moderate | Within-study bias |
|  | Indirect evidence | | | | | | | | | |
| FES:NMES+rPMS | | 0 | Some concerns | Low risk | No concerns | Some concerns | No concerns | Major concerns | Very low | Within-study bias, Imprecision, Incoherence |
| FES:NMES+TMS | | 0 | Some concerns | Low risk | No concerns | No concerns | No concerns | Major concerns | Very low | Within-study bias, Incoherence |
| FES:rPMS | | 0 | Some concerns | Low risk | No concerns | No concerns | No concerns | Major concerns | Very low | Within-study bias, Incoherence |
| FES+TMS:NMES | | 0 | Some concerns | Low risk | No concerns | Some concerns | No concerns | Major concerns | Very low | Within-study bias, Imprecision, Incoherence |
| FES+TMS:NMES+rPMS | | 0 | Some concerns | Low risk | No concerns | Some concerns | No concerns | Major concerns | Very low | Within-study bias, Imprecision, Incoherence |

Abbreviations: FES, functional electrical stimulation; NMES, neuromuscular electrical stimulation; Rehab, conventional rehabilitation; rPMS, repetitive electrical magnetic stimulation; TMS, transcranial magnetic stimulation.

**A)** Upper extremity functions with FMA (Cont.)

| **Comparison** | **Number of studies** | **Within-study bias** | **Reporting bias** | **Indirectness** | **Imprecision** | **Heterogeneity** | **Incoherence** | **Confidence rating** | **Reasons for downgrading** |
| --- | --- | --- | --- | --- | --- | --- | --- | --- | --- |
| Indirect evidence | | | | | | | | | |
| FES+TMS:NMES+TMS | 0 | Some concerns | Low risk | No concerns | Some concerns | Some concerns | Major concerns | Very low | Within-study bias, Imprecision, Heterogeneity, Incoherence |
| FES+TMS:Rehab | 0 | Some concerns | Low risk | No concerns | Some concerns | Some concerns | Major concerns | Very low | Within-study bias, Imprecision, Heterogeneity, Incoherence |
| FES+TMS:rPMS | 0 | Some concerns | Low risk | No concerns | Some concerns | Some concerns | Major concerns | Very low | Within-study bias, Imprecision, Heterogeneity, Incoherence |
| FES+TMS:TMS | 0 | Some concerns | Low risk | No concerns | Some concerns | No concerns | Major concerns | Very low | Within-study bias, Imprecision, Incoherence |
| NMES:rPMS | 0 | Some concerns | Low risk | No concerns | No concerns | No concerns | Major concerns | Very low | Within-study bias, Incoherence |
| NMES+rPMS:NMES+TMS | 0 | Some concerns | Low risk | No concerns | Some concerns | No concerns | Major concerns | Very low | Within-study bias, Imprecision, Incoherence |
| NMES+rPMS:Rehab | 0 | Some concerns | Low risk | No concerns | No concerns | Some concerns | Major concerns | Very low | Within-study bias, Heterogeneity, Incoherence |
| NMES+rPMS:rPMS | 0 | Some concerns | Low risk | No concerns | Some concerns | No concerns | Major concerns | Very low | Within-study bias, Imprecision, Incoherence |
| NMES+rPMS:TMS | 0 | Some concerns | Low risk | No concerns | Some concerns | No concerns | Major concerns | Very low | Within-study bias, Imprecision, Incoherence |
| NMES+TMS:rPMS | 0 | Some concerns | Low risk | No concerns | No concerns | Some concerns | Major concerns | Very low | Within-study bias, Heterogeneity, Incoherence |

Abbreviations: FES, functional electrical stimulation; NMES, neuromuscular electrical stimulation; Rehab, conventional rehabilitation; rPMS, repetitive electrical magnetic stimulation; TMS, transcranial magnetic stimulation.

**B)** ADL with BI

| **Comparison** | **Number of studies** | **Within-study bias** | **Reporting bias** | **Indirectness** | **Imprecision** | **Heterogeneity** | **Incoherence** | **Confidence rating** | **Reasons for downgrading** |
| --- | --- | --- | --- | --- | --- | --- | --- | --- | --- |
| Mixed Evidence | | | | | | | | | |
| FES:Rehab | 2 | Some concerns | Low risk | No concerns | No concerns | No concerns | No concerns | Moderate | Within-study bias |
| FES:TMS | 1 | Some concerns | Low risk | No concerns | No concerns | No concerns | No concerns | Moderate | Within-study bias |
| NMES:NMES+rPMS | 1 | Some concerns | Low risk | No concerns | No concerns | No concerns | Major concerns | Very low | Within-study bias, Incoherence |
| NMES:Rehab | 3 | Some concerns | Low risk | No concerns | No concerns | No concerns | Major concerns | Very low | Within-study bias, Incoherence |
| NMES+TMS:Rehab | 1 | Some concerns | Low risk | No concerns | Some concerns | No concerns | Major concerns | Very low | Within-study bias, Imprecision, Incoherence |
| NMES+TMS:TMS | 1 | Some concerns | Low risk | No concerns | Some concerns | No concerns | Major concerns | Very low | Within-study bias, Imprecision, Incoherence |
| Rehab:rPMS | 1 | Some concerns | Low risk | No concerns | No concerns | Some concerns | Major concerns | Very low | Within-study bias, Heterogeneity, Incoherence |
| Rehab:TMS | 5 | Some concerns | Low risk | No concerns | No concerns | No concerns | No concerns | Moderate | Within-study bias |
| rPMS:TMS | 1 | Some concerns | Low risk | No concerns | No concerns | No concerns | Major concerns | Very low | Within-study bias, Incoherence |

Abbreviations: FES, functional electrical stimulation; NMES, neuromuscular electrical stimulation; Rehab, conventional rehabilitation; rPMS, repetitive electrical magnetic stimulation; TMS, transcranial magnetic stimulation.

**B)** ADL with BI (Cont.)

| **Comparison** | **Number of studies** | **Within-study bias** | **Reporting bias** | **Indirectness** | **Imprecision** | **Heterogeneity** | **Incoherence** | **Confidence rating** | **Reasons for downgrading** |
| --- | --- | --- | --- | --- | --- | --- | --- | --- | --- |
| Indirect evidence | | | | | | | | | |
| FES:NMES | 0 | Some concerns | Low risk | No concerns | No concerns | Some concerns | Major concerns | Very low | Within-study bias, Heterogeneity, Incoherence |
| FES:NMES+rPMS | 0 | Some concerns | Low risk | No concerns | No concerns | No concerns | Major concerns | Very low | Within-study bias, Incoherence |
| FES:NMES+TMS | 0 | Some concerns | Low risk | No concerns | Some concerns | No concerns | Major concerns | Very low | Within-study bias, Imprecision, Incoherence |
| FES:rPMS | 0 | Some concerns | Low risk | No concerns | No concerns | No concerns | Major concerns | Very low | Within-study bias, Incoherence |
| NMES:NMES+TMS | 0 | Some concerns | Low risk | No concerns | Some concerns | Some concerns | Major concerns | Very low | Within-study bias, Imprecision, Heterogeneity, Incoherence |
| NMES:rPMS | 0 | Some concerns | Low risk | No concerns | Some concerns | No concerns | Major concerns | Very low | Within-study bias, Imprecision, Incoherence |
| NMES:TMS | 0 | Some concerns | Low risk | No concerns | No concerns | No concerns | Major concerns | Very low | Within-study bias, Incoherence |
| NMES+rPMS:NMES+TMS | 0 | Some concerns | Low risk | No concerns | Some concerns | No concerns | Major concerns | Very low | Within-study bias, Imprecision, Incoherence |
| NMES+rPMS:Rehab | 0 | Some concerns | Low risk | No concerns | No concerns | No concerns | Major concerns | Very low | Within-study bias, Incoherence |
| NMES+rPMS:rPMS | 0 | Some concerns | Low risk | No concerns | No concerns | Some concerns | Major concerns | Very low | Within-study bias, Heterogeneity, Incoherence |

Abbreviations: FES, functional electrical stimulation; NMES, neuromuscular electrical stimulation; Rehab, conventional rehabilitation; rPMS, repetitive electrical magnetic stimulation; TMS, transcranial magnetic stimulation.

**B)** ADL with BI (Cont.)

| **Comparison** | **Number of studies** | **Within-study bias** | **Reporting bias** | **Indirectness** | **Imprecision** | **Heterogeneity** | **Incoherence** | **Confidence rating** | **Reasons for downgrading** |
| --- | --- | --- | --- | --- | --- | --- | --- | --- | --- |
| Indirevt evidence | | | | | | | | | |
| NMES+rPMS:TMS | 0 | Some concerns | Low risk | No concerns | No concerns | No concerns | Major concerns | Very low | Within-study bias, Incoherence |
| NMES+TMS:rPMS | 0 | Some concerns | Low risk | No concerns | Some concerns | No concerns | Major concerns | Very low | Within-study bias, Imprecision, Incoherence |

Abbreviations: FES, functional electrical stimulation; NMES, neuromuscular electrical stimulation; Rehab, conventional rehabilitation; rPMS, repetitive electrical magnetic stimulation; TMS, transcranial magnetic stimulation.

**C)** Spasticity with MAS

| **Comparison** | **Number of studies** | **Within-study bias** | **Reporting bias** | **Indirectness** | **Imprecision** | **Heterogeneity** | **Incoherence** | **Confidence rating** | **Reasons for downgrading** |
| --- | --- | --- | --- | --- | --- | --- | --- | --- | --- |
| Mixed Evidence | | | | | | | | | |
| FES:Rehab | 3 | Some concerns | Low risk | No concerns | Major concerns | No concerns | No concerns | Very low | Within-study bias, Imprecision |
| NMES:NMES+TMS | 1 | Some concerns | Low risk | No concerns | Some concerns | Some concerns | No concerns | Low | Within-study bias, Imprecision, Heterogeneity |
| NMES:Rehab | 4 | Some concerns | Some concerns | No concerns | Some concerns | Some concerns | No concerns | Very low | Within-study bias, Reporting bias, Imprecision, Heterogeneity |
| NMES:TMS | 1 | Some concerns | Low risk | No concerns | Some concerns | Some concerns | No concerns | Low | Within-study bias, Imprecision, Heterogeneity |
| NMES+TMS:Rehab | 2 | Some concerns | Low risk | No concerns | Some concerns | Some concerns | Major concerns | Very low | Within-study bias, Imprecision, Heterogeneity, Incoherence |
| NMES+TMS:TMS | 2 | Some concerns | Low risk | No concerns | Some concerns | Some concerns | No concerns | Low | Within-study bias, Imprecision, Heterogeneity |
| Rehab:rPMS | 1 | Some concerns | Low risk | No concerns | Some concerns | Some concerns | No concerns | Low | Within-study bias, Imprecision, Heterogeneity |
| Rehab:TMS | 8 | Some concerns | Low risk | No concerns | No concerns | Major concerns | No concerns | Very low | Within-study bias, Heterogeneity |

Abbreviations: FES, functional electrical stimulation; NMES, neuromuscular electrical stimulation; Rehab, conventional rehabilitation; rPMS, repetitive electrical magnetic stimulation; TMS, transcranial magnetic stimulation.

**C)** Spasticity with MAS (Cont.)

| **Comparison** | **Number of studies** | **Within-study bias** | **Reporting bias** | **Indirectness** | **Imprecision** | **Heterogeneity** | **Incoherence** | **Confidence rating** | **Reasons for downgrading** |
| --- | --- | --- | --- | --- | --- | --- | --- | --- | --- |
| Indirect evidence | | | | | | | | | |
| FES:NMES | 0 | Some concerns | Low risk | No concerns | Major concerns | No concerns | No concerns | Very low | Within-study bias, Imprecision |
| FES:NMES+TMS | 0 | Some concerns | Low risk | No concerns | Major concerns | No concerns | No concerns | Very low | Within-study bias, Imprecision |
| FES:rPMS | 0 | Some concerns | Low risk | No concerns | Major concerns | No concerns | No concerns | Very low | Within-study bias, Imprecision |
| FES:TMS | 0 | Some concerns | Low risk | No concerns | Major concerns | No concerns | No concerns | Very low | Within-study bias, Imprecision |
| NMES:rPMS | 0 | Some concerns | Low risk | No concerns | Major concerns | No concerns | No concerns | Very low | Within-study bias, Imprecision |
| NMES+TMS:rPMS | 0 | Some concerns | Low risk | No concerns | Some concerns | Some concerns | No concerns | Low | Within-study bias, Imprecision, Heterogeneity |
| rPMS:TMS | 0 | Some concerns | Low risk | No concerns | Some concerns | Some concerns | No concerns | Low | Within-study bias, Imprecision, Heterogeneity |

Abbreviations: FES, functional electrical stimulation; NMES, neuromuscular electrical stimulation; Rehab, conventional rehabilitation; rPMS, repetitive electrical magnetic stimulation; TMS, transcranial magnetic stimulation.

**Appendix 10:** Subgroup analysis of NMA on the ADL outcome measured with BI at the end of intervention course

1. Network map

Network map of the ADL outcome using BI at the end of the intervention in the acute- sub-acute stroke subgroup, with nodes and edges weighted by the number of studies and included patients, respectively

**B)** Multiple treatment comparisons of interventions on ADL measured with BI in the acute-sub-acute subgroup

| **Reference treatment** |  | | **Mean difference** | | | | | |
| --- | --- | --- | --- | --- | --- | --- | --- | --- |
|  | **Rehab** | **NMES+rPMS** | | **NMES+TMS** | **rPMS** | **TMS** | **FES** | **NMES** |
| Rehab | 10.5, 0.0 | 4.79 (-0.30, 9.87) | | 30.9 (5.17, 56.62) | 21.65 (7.17, 36.11) | 12.24 (6.01, 18.47) | 8.34 (2.37, 14.31) | -0.21 (-1.85, 1.42) |
| NMES+rPMS | -4.79 (-9.87, 0.30) | 36.9, 0.0 | | 26.11 (-0.10, 52.33) | 16.86 (1.52, 32.20) | 7.45 (-0.59, 15.50) | 3.55 (-4.29, 11.40) | -5.00 (-9.82, -0.18) |
| NMES+TMS | -30.9 (-56.62,  -5.17) | -26.11 (-52.33, 0.10) | | 93.3, 74.9 | -9.25 (-38.76, 20.26) | -18.66 (-45.13, 7.80) | -22.56 (-48.96, 3.84) | -31.11 (-56.89,  -5.34) |
| rPMS | -21.65 (-36.11,  -7.17) | -16.86 (-32.20, -1.52) | | 9.25 (-20.26, 38.76) | 84.7, 24.1 | -9.41 (-22.47, 3.65) | -13.31 (-28.96, 2.35) | -21.86 (-36.43,  -7.30) |
| TMS | -12.24 (-18.47,  -6.01) | -7.45 (-15.50, 0.59) | | 18.66 (-7.80, 45.13) | 9.41 (-3.65, 22.47) | 65.9, 0.7 | -3.89 (-12.53, 4.73) | ­­­-12.45 (-18.90,  -6.01) |
| FES | -8.34 (-14.31,  -2.37) | -3.55 (-11.40, 4.29) | | 22.56 (-3.84, 48.96) | 13.31 (-2.35, 28.96) | 3.89 (-4.73, 12.53) | 51.3, 0.3 | -8.55 (-14.75, -2.36) |
| NMES | 0.21 (-1.42, 1.85) | 5.00 (0.18, 9.82) | | 31.11 (5.34, 56.89) | 21.86 (7.30, 36.43) | 12.45 (6.01, 18.90) | 8.55 (2.36, 14.75) | 7.4, 0.0 |

Abbreviations: FES, functional electrical stimulation; NMES, neuromuscular electrical stimulation; Rehab, conventional rehabilitation; rPMS, repetitive electrical magnetic stimulation; TMS, transcranial magnetic stimulation.

Results in the off-diagonal cells are the mean differences and 95% confidence intervals of the BI from the network meta-analysis. Each diagonal cell contains the surface under the cumulative ranking and probability of being the best treatment of each intervention.

**Appendix 11:** Subgroup analyses of NMA on the spasticity outcome measured with MAS at the end of intervention course

**A)** Network maps


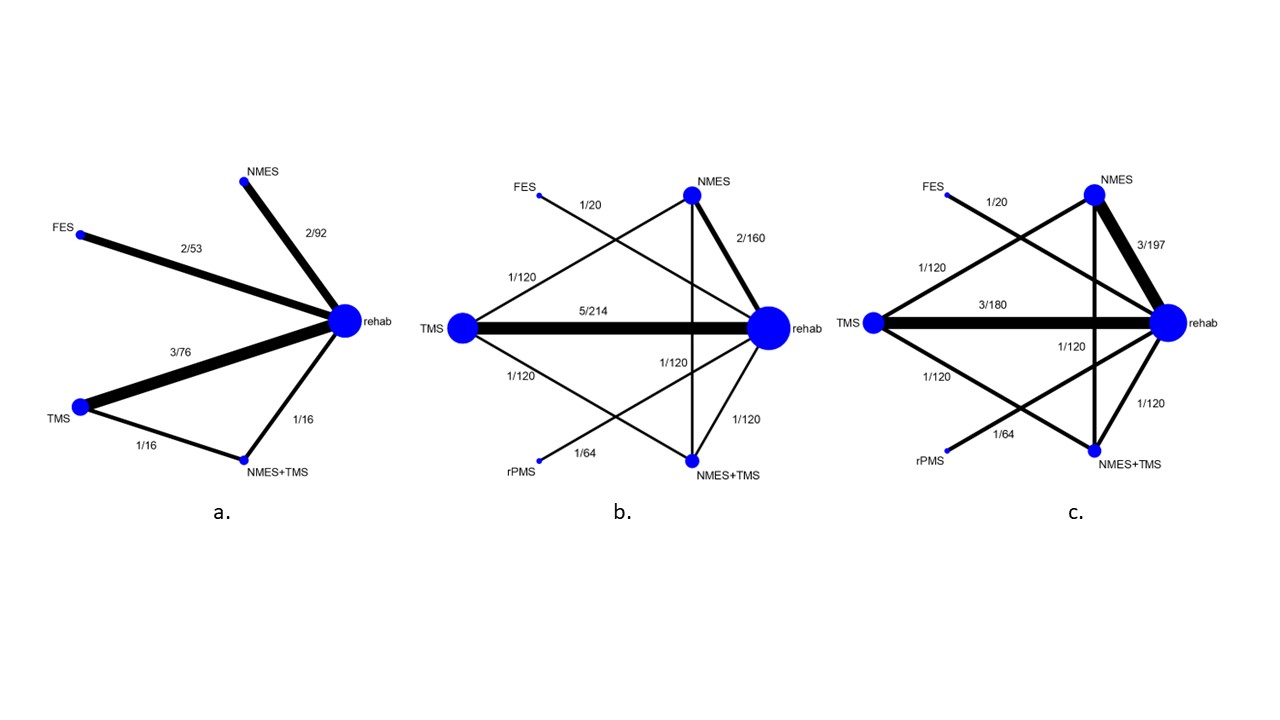


(a.) acute-sub-acute stroke subgroup, (b.) chronic stroke subgroup, and (c.) more severe stroke subgroup, with nodes and edges weighted by the number of studies and included patients, respectively

**B)** Multiple treatment comparisons of interventions on spasticity measured with MAS in the acute-sub-acute subgroup

| **Reference treatment** | **Mean difference** | | | | |
| --- | --- | --- | --- | --- | --- |
|  | **Rehab** | **NMES+TMS** | **TMS** | **FES** | **NMES** |
| Rehab | 44.5, 5.1 | -0.26 (-1.29,0.77) | -0.01 (-0.70,0.67) | 0.10 (-0.74,0.94) | -0.10 (-0.81,0.60) |
| NMES+TMS | 0.26 (-0.77,1.29) | 68.8, 46.0 | 0.24 (-0.79,1.27) | 0.36 (-0.97,1.69) | 0.15 (-1.10,1.40) |
| TMS | 0.01 (-0.67,0.70) | -0.24 (-1.27,0.79) | 46.8, 12.6 | 0.12 (-0.97,1.20) | -0.09 (-1.07,0.89) |
| FES | -0.10 (-0.94,0.74) | -0.36 (-1.69,0.97) | -0.12 (-1.20,0.97) | 34.9, 12.5 | -0.21 (-1.29,0.88) |
| NMES | 0.10 (-0.60,0.81) | -0.15 (-1.40,1.10) | 0.09 (-0.89,1.07) | 0.21 (-0.88,1.29) | 55.0, 23.8 |

Abbreviations: FES, functional electrical stimulation; NMES, neuromuscular electrical stimulation; Rehab, conventional rehabilitation; TMS, transcranial magnetic stimulation.

Results in the off-diagonal cells are the mean differences and 95% confidence intervals of the MAS from the network meta-analysis. Each diagonal cell contains the surface under the cumulative ranking and probability of being the best treatment of each intervention.

**C)** Multiple treatment comparisons of interventions on spasticity measured with MAS in the chronic subgroup

| **Reference treatment** | **Mean difference** | | | | | |
| --- | --- | --- | --- | --- | --- | --- |
|  | **Rehab** | **NMES+TMS** | **rPMS** | **TMS** | **FES** | **NMES** |
| Rehab | 38.0, 0.5 | 0.36 (-0.51,1.22) | -0.51 (-1.52,0.50) | -0.04 (-0.51,0.43) | -0.34 (-1.58,0.90) | -0.26 (-0.96,0.44) |
| NMES+TMS | -0.36 (-1.22,0.51) | 16.0, 0.9 | -0.87 (-2.20,0.47) | -0.40 (-1.28,0.48) | -0.70 (-2.21,0.82) | -0.61 (-1.55,0.32) |
| rPMS | 0.51 (-0.50,1.52) | 0.87 (-0.47,2.20) | 74.2, 42.3 | 0.47 (-0.65,1.58) | 0.17 (-1.43,1.77) | 0.25 (-0.98,1.48) |
| TMS | 0.04 (-0.43,0.51) | 0.40 (-0.48,1.28) | -0.47 (-1.58,0.65) | 43.3, 3.9 | -0.30 (-1.63,1.03) | -0.22 (-0.97,0.53) |
| FES | 0.34 (-0.90,1.58) | 0.70 (-0.82,2.21) | -0.17 (-1.77,1.43) | 0.30 (-1.03,1.63) | 63.4, 32.9 | 0.08 (-1.34,1.51) |
| NMES | 0.26 (-0.44,0.96) | 0.61 (-0.32,1.55) | -0.25 (-1.48,0.98) | 0.22 (-0.53,0.97) | -0.08 (-1.51,1.34) | 65.1, 19.5 |

Abbreviations: FES, functional electrical stimulation; NMES, neuromuscular electrical stimulation; Rehab, conventional rehabilitation; rPMS, repetitive electrical magnetic stimulation; TMS, transcranial magnetic stimulation.

Results in the off-diagonal cells are the mean differences and 95% confidence intervals of the MAS from the network meta-analysis. Each diagonal cell contains the surface under the cumulative ranking and probability of being the best treatment of each intervention.

**D)** Multiple treatment comparisons of interventions on spasticity measured with MAS in the more severe (FMA baseline < 25) subgroup

| **Reference treatment** | **Mean difference** | | | | | |
| --- | --- | --- | --- | --- | --- | --- |
|  | **Rehab** | **NMES+TMS** | **rPMS** | **TMS** | **FES** | **NMES** |
| Rehab | 50.3, 1.6 | 0.50 (-0.28,1.28) | -0.51 (-1.42,0.40) | 0.14 (-0.39,0.68) | -0.34 (-1.50,0.82) | -0.00 (-0.54,0.53) |
| NMES+TMS | -0.50 (-1.28,0.28) | 12.0, 0.5 | -1.01 (-2.21,0.19) | -0.36 (-1.17,0.45) | -0.84 (-2.24,0.56) | -0.50 (-1.31,0.31) |
| rPMS | 0.51 (-0.40,1.42) | 1.01 (-0.19,2.21) | 81.4, 51.4 | 0.65 (-0.41,1.71) | 0.17 (-1.31,1.65) | 0.51 (-0.55,1.56) |
| TMS | -0.14 (-0.68,0.39) | 0.36 (-0.45,1.17) | -0.65 (-1.71,0.41) | 35.1, 2.6 | -0.48 (-1.76,0.80) | -0.14 (-0.79,0.50) |
| FES | 0.34 (-0.82,1.50) | 0.84 (-0.56,2.24) | -0.17 (-1.65,1.31) | 0.48 (-0.80,1.76) | 69.7, 37.4 | 0.34 (-0.94,1.62) |
| NMES | 0.00 (-0.53,0.54) | 0.50 (-0.31,1.31) | -0.51 (-1.56,0.55) | 0.14 (-0.50,0.79) | -0.34 (-1.62,0.94) | 51.4, 6.5 |

Abbreviations: FES, functional electrical stimulation; NMES, neuromuscular electrical stimulation; Rehab, conventional rehabilitation; rPMS, repetitive electrical magnetic stimulation; TMS, transcranial magnetic stimulation.

Results in the off-diagonal cells are the mean differences and 95% confidence intervals of the MAS from the network meta-analysis. Each diagonal cell contains the surface under the cumulative ranking and probability of being the best treatment of each intervention.

**Appendix 12:** Comparison-adjusted funnel plots for network meta-analysis


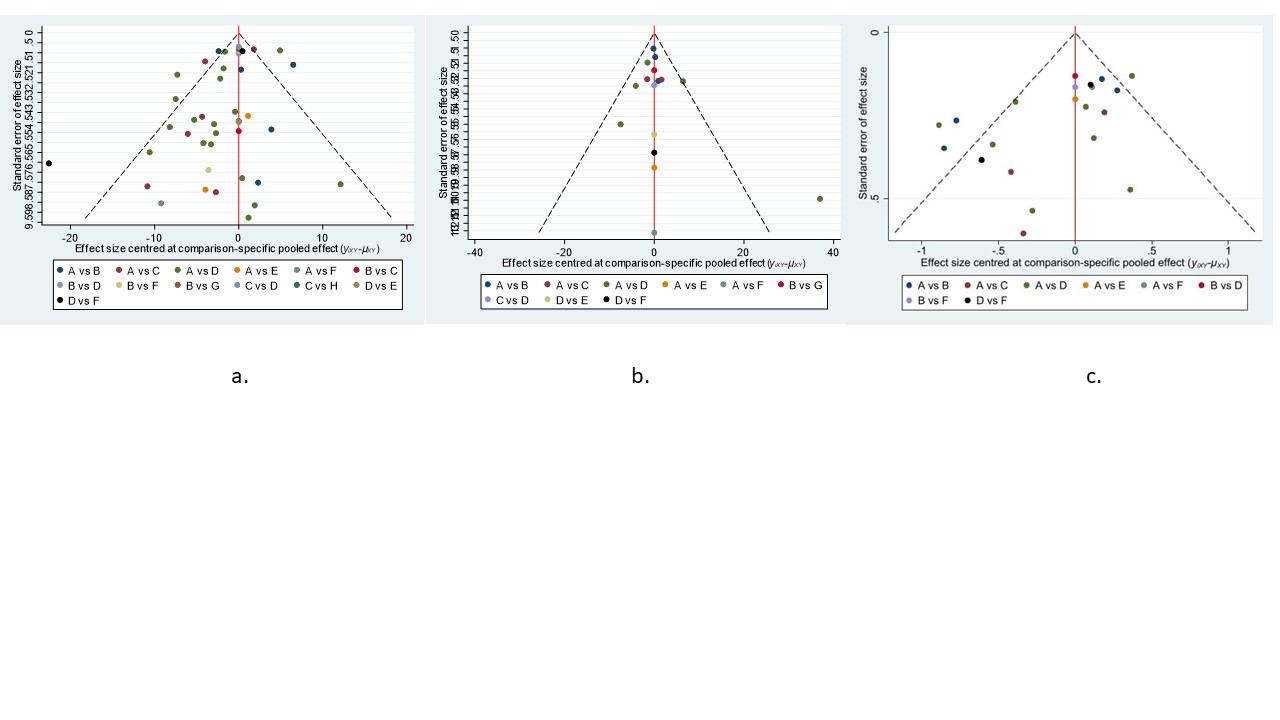


A, Rehab; B, NMES; C, FES; D, TMS; E, rPMS; F, NMES+TMS; G, NMES+rPMS; H, FES+TMS

Abbreviations: FES, functional electrical stimulation; NMES, neuromuscular electrical stimulation; Rehab, conventional rehabilitation; rPMS, repetitive electrical magnetic stimulation; TMS, transcranial magnetic stimulation.

(a.) upper extremity functions outcome using FMA (b.) ADL outcome using BI (c.) spasticity outcome using MAS at the end of intervention

**Appendix 13:** Multiple treatment comparisons of interventions on upper extremity function measured by FMA at the end of intervention courses, sensitivity analysis excludes studies that contain other neurological diseases

| **Reference treatment** | **Unstandardized mean difference** | | | | | | | |
| --- | --- | --- | --- | --- | --- | --- | --- | --- |
|  | **Rehab** | **FES+TMS** | **NMES+rPMS** | **NMES+TMS** | **rPMS** | **TMS** | **FES** | **NMES** |
| **Rehab** | 14.6, 0.0 | 0.40 (-9.51, 10.31) | 14.69 (9.89, 19.48) | 6.10 (2.47, 9 .73) | -0.73 (-16.49, 15.03) | 4.07 (0.30, 7.84) | 3.60 (0.11, 7.09) | 9.09 (5.99, 12.19) |
| **FES+TMS** | -0.40 (-10.31, 9.51) | 25.5, 0.5 | 14.29 (3.29, 25.29) | 5.70 (-4.85, 16.25) | -1.13 (-19.75, 17.49) | 3.66 (-6.93, 14.27) | 3.20 (-6.07, 12.47) | 8.69 (-1.68, 19.06) |
| **NMES+rPMS** | -14.69 (-19.48,  -9.89) | -14.29 (-25.29,  -3.29) | 99.4, 95.8 | -8.59 (-14.60, -2.58) | -15.42 (-31.89,  1.05) | -10.62 (-16.72,  -4.52) | -11.09 (-17.00,  -5.18) | -5.60 (-9.25,  -1.94) |
| **NMES+TMS** | -6.10 (-9.73,  -2.47) | -5.70 (-16.25, 4.85) | 8.59 (2.58, 14.60) | 63.2, 0.1 | -6.83 (-23.00, 9.34) | -2.03 (-5.79, 1.73) | -2.50 (-7.53, 2.53) | 2.99 (-1.78, 7.77) |
| **rPMS** | 0.73 (-15.03, 16.49) | 1.13 (-17.49, 19.75) | 15.42 (-1.05, 31.89) | 6.83 (-9.34, 23.00) | 26.9, 3.6 | 4.80 (-11.81, 21.01) | 4.33 (-11.81, 20.47) | 9.82 (-6.24, 25.88) |
| **TMS** | -4.07 (-7.84,  -0.30) | -3.66 (-14.27, 6.93) | 10.62 (4.52, 16.72) | 2.03 (-1.73, 5.79) | -4.80 (-21.01, 11.41) | 45.7, 0.0 | -0.47 (-5.61, 4.67) | 5.02 (0.14, 9.91) |
| **FES** | -3.60 (-7.09,  -0.11) | -3.20 (-12.47, 6.07) | 11.09 (5.18, 17.00) | 2.50 (-2.53, 7.53) | -4.33 (-20.47, 11.81) | 0.47 (-4.67, 5.61) | 43.3, 0.0 | 5.49 (0.43, 10.14) |
| **NMES** | -9.09 (-12.19,  -5.99) | -8.69 (-19.06, 1.68) | 5.60 (1.94, 9.25) | -2.99 (-7.77, 1.78) | -9.82 (-25.88, 6.24) | -5.02 (-9.91,  -0.14) | -5.49 (-10.14,  -0.43) | 81.4, 0.0 |

Abbreviations: FES, functional electrical stimulation; NMES, neuromuscular electrical stimulation; Rehab, conventional rehabilitation; rPMS, repetitive electrical magnetic stimulation; TMS, transcranial magnetic stimulation.

Results in the off-diagonal cells are the mean difference and 95% confidence intervals of the FMA from the network meta-analysis. Each diagonal cell contains the surface under the cumulative ranking and probability of being the best treatment of each intervention.
